# The Role of Epigenetic Modifications in Human Cancers and the Use of Natural Compounds as Epidrugs: Mechanistic Pathways and Pharmacodynamic Actions

**DOI:** 10.3390/biom12030367

**Published:** 2022-02-25

**Authors:** Abdelhakim Bouyahya, Hamza Mechchate, Loubna Oumeslakht, Ikrame Zeouk, Sara Aboulaghras, Abdelaali Balahbib, Gokhan Zengin, Mohammad Amjad Kamal, Monica Gallo, Domenico Montesano, Nasreddine El Omari

**Affiliations:** 1 Laboratory of Human Pathologies Biology, Department of Biology, Faculty of Sciences, Genomic Center of Human Pathologies, Mohammed V University in Rabat, Rabat 10106, Morocco; 2 Laboratory of Biotechnology, Environment, Agri-Food, and Health (LBEAS), Faculty of Sciences, University Sidi Mohamed Ben Abdellah (USMBA), Fez 1796, Morocco; hamza.mechchate@usmba.ac.ma; 3 Institute of Biological Sciences (ISSB-P), Mohammed VI Polytechnic University (UM6P), Benguerir 43150, Morocco; loubnaoumeslakht@gmail.com; 4 Laboratory of Microbial Biotechnology, Faculty of Sciences and Technology, Sidi Mohamed Ben Abdellah University, Fez 1976, Morocco; ikramezeouk20@gmail.com; 5 Physiology and Physiopathology Team, Department of Biology, Mohammed V University in Rabat, Rabat 10106, Morocco; sara.aboulghras@gmail.com; 6 Laboratory of Biodiversity, Ecology, and Genome, Faculty of Sciences, Mohammed V University in Rabat, Rabat 10106, Morocco; balahbib.abdo@gmail.com; 7 Physiology and Biochemistry Laboratory, Department of Biology, Selcuk University, Campus, Konya 42130, Turkey; gokhanzengin@selcuk.edu.tr; 8 King Fahd Medical Research Center, King Abdulaziz University, P.O. Box 80216, Jeddah 21589, Saudi Arabia; prof.ma.kamal@gmail.com; 9 West China School of Nursing/Institutes for Systems Genetics, Frontiers Science Center for Disease-Related Molecular Network, West China Hospital, Sichuan University, Chengdu 610041, China; 10 Enzymoics, Novel Global Community Educational Foundation, 7 Peterlee Place, Hebersham, NSW 2770, Australia; 11 Department of Molecular Medicine and Medical Biotechnology, University of Naples Federico II, Via Pansini 5, 80131 Naples, Italy; 12 Department of Pharmacy, University of Naples Federico II, Via D. Montesano 49, 80131 Naples, Italy; domenico.montesano@unina.it; 13 Laboratory of Histology, Embryology, and Cytogenetic, Faculty of Medicine and Pharmacy, Mohammed V University in Rabat, Rabat 10100, Morocco; nasrelomari@gmail.com

**Keywords:** epidrugs, cancer, DNMT, HDAC, cancer therapy, pharmacodynamic

## Abstract

Cancer is a complex disease resulting from the genetic and epigenetic disruption of normal cells. The mechanistic understanding of the pathways involved in tumor transformation has implicated a priori predominance of epigenetic perturbations and a posteriori genetic instability. In this work, we aimed to explain the mechanistic involvement of epigenetic pathways in the cancer process, as well as the abilities of natural bioactive compounds isolated from medicinal plants (flavonoids, phenolic acids, stilbenes, and ketones) to specifically target the epigenome of tumor cells. The molecular events leading to transformation, angiogenesis, and dissemination are often complex, stochastic, and take turns. On the other hand, the decisive advances in genomics, epigenomics, transcriptomics, and proteomics have allowed, in recent years, for the mechanistic decryption of the molecular pathways of the cancerization process. This could explain the possibility of specifically targeting this or that mechanism leading to cancerization. With the plasticity and flexibility of epigenetic modifications, some studies have started the pharmacological screening of natural substances against different epigenetic pathways (DNA methylation, histone acetylation, histone methylation, and chromatin remodeling) to restore the cellular memory lost during tumor transformation. These substances can inhibit DNMTs, modify chromatin remodeling, and adjust histone modifications in favor of pre-established cell identity by the differentiation program. Epidrugs are molecules that target the epigenome program and can therefore restore cell memory in cancerous diseases. Natural products isolated from medicinal plants such as flavonoids and phenolic acids have shown their ability to exhibit several actions on epigenetic modifiers, such as the inhibition of DNMT, HMT, and HAT. The mechanisms of these substances are specific and pleiotropic and can sometimes be stochastic, and their use as anticancer epidrugs is currently a remarkable avenue in the fight against human cancers.

## 1. Introduction

Cancer is a major health issue worldwide. It was originally perceived as a genetic disease. However, several studies have shown that cancer is a complex and multifactorial group of diseases developed by the accumulation of genetic and epigenetic alterations. Genetic alterations include chromosomal aberrations, mutations, translocations, amplifications, insertions, and deletions. These alterations can be developed due to exposure to different external, and internal factors, and they may be repaired by DNA repair mechanisms, or they may be accumulated in cells. Their accumulation reflects the organism’s life history and, over time, may interfere with different cellular processes such as cell-cycle control, differentiation, cell death or maintenance of genomic integrity, and induce cancerization. However, these alterations are not the unique cause of cancer; epigenetic alterations are also key factors that are implicated in all cancer development stages. Deregulation of the cell epigenome, including DNA methylation, chromatin remodeling, non-coding RNAs, and histone modifications (methylation, acetylation, etc.) can alter different cellular mechanisms such as transcriptional machinery, gene expression, DNA replication and repair. These deregulations are implicated in every step of cancer development and progression, from the early stages to the advanced stages. They also contribute to all cancer hallmarks, by activating or inhibiting several signaling pathways [1]. Since epigenetic modifications are reversible, several studies demonstrated the importance of using epidrugs to reprogram the epigenome of cancer cells in order to ensure the control of tumor growth, block invasion, and metastasis, and treat tumor resistance to some therapies [2]. In this review, we discuss the different alterations occurring in the cancer genome and epigenome, and we focus on the recently discovered phytochemicals that target the cancer epigenome and outline their mechanisms and implications in cancer treatment.

## 2. Overview on Cancer

**Figure 1:**
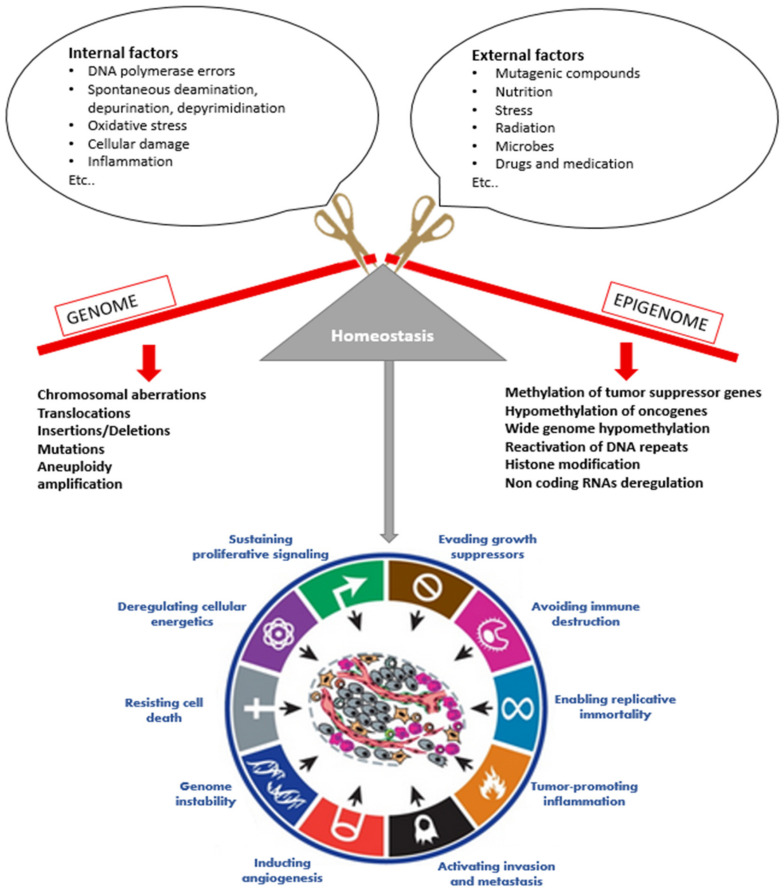
Cancer development through epigenetic modifications and genetic deregulation due to internal and external factors. *External factors (such as oxidative stress, inflammation, and cellular damage) and internal factors (including nutrition, stress, microbes, and drugs) can affect genome and epigenom at different levels. The perturbation of genome (inducing of mutations, translocations, and deletions) and/or epigenom (ectopic methylation and histone modification) can induce homeostasis disruption and therefore cell transformation (cancer)*. Reproduced with permission from Robert C. Bast Jr., Carlo M. Croce William N. Hait, Waun Ki Hong, Donald W. Kufe, Martine Piccart-Gebart, Raphael E. Pollock, Ralph R. Weichselbaum, Hongyang Wang, and James F. Holland. Holland-Frei Cancer Medicine, 9th Edition; published by John Wiley and Sons, 2016.

Cancer is a complex, diverse and multifactorial family of diseases that differs in molecular and phenotypical characteristics. It is characterized by abnormal cell growth caused by multiple changes in gene expression leading to a deregulated balance between cell proliferation and cell death [3,4]. As defined by Hanahan and Weinberg, cancer is characterized by different hallmarks consisting of maintaining proliferative signaling, avoiding growth suppressors, resisting cell death, enabling replicative immortality, inducing angiogenesis, activating invasion and metastasis, deregulating cellular energetics and metabolism, preventing immune destruction, and promoting inflammation and genome instability (Figure 1) [5].

Cancer may be induced by various endogenous and exogenous factors (Figure 1). Endogenous factors include DNA mismatches caused by DNA polymerase errors during replication and genetic instability caused by spontaneous deamination, depurination, and depyrimidination of DNA bases [6]. In addition, oxidative stress is highly implicated in all cancer stages; it may activate a number of signal transduction pathways that transcribe cell growth regulatory genes, and may also induce mutations and DNA damage by releasing reactive oxygen species (ROS) that oxidize DNA bases [7]. Additionally, cellular stress, damage, inflammation, and necrotic cell death induce the release of endogenous damage-associated molecular pattern (DAMP) molecules that activate the immune response. However, the persistence of these molecules induces chronic inflammation associated with carcinogenesis [8]. Chronic inflammation also releases free radicals and aldehydes that induce gene mutations and post-translational modifications of cancer-related proteins [9].

Exogenous factors include exposure to mutagenic compounds, tobacco and alcohol consumption, nutrition, stress, ultraviolet radiations, and reactive oxygen species (ROS) that induce structural changes in DNA by modifying nucleotide bases, inducing DNA strand breakage, inter- and intra-strand crosslinks, and DNA-protein crosslinks [10]. Microbes, including viruses such as human papillomavirus, are associated with cervical cancer, bacteria such as *Helicobacter* are associated with gastric cancer, and parasites such as *Schistosoma* are associated with urinary bladder cancer. Microbe oncoproteins and toxins may also induce inflammation, DNA damage in host cell, and deregulation of host cell signaling pathways that are involved in cell proliferation, apoptosis, differentiation, and immune signaling [11].

Epigenetic modifications constitute another factor that plays a crucial role in carcinogenesis. Indeed, recent progress revealed that the cancer epigenome undergoes an overall change in its programming, including DNA methylation, disruptions in histone post-translational modification patterns, alterations of chromatin remodeling, and deregulation of non-coding RNAs expression. Hence, epigenetic modifications become a promising new therapy for cancer treatment [12].

There are three categories of genes involved in the development of cancer; proto-oncogenes, tumor suppressor genes, and DNA repair genes. Proto-oncogenes are normal genes that encode growth factors, receptors, intracellular transducers, and nuclear proteins. These genes are implicated in numerous processes of cell signaling, cell cycle, cell differentiation, embryogenesis, and cell proliferation. Genetic alterations such as a punctual mutation, a gene amplification, base insertion, a deletion or a translocation in a single allele of these genes, may induce activation of the gene’s transcription (gain of function), and consequently, cell cycle activation, and alteration of cell metabolic pathways. In this case, the genes are called oncogenes [3].

Tumor suppressor genes are involved in the cell cycle, maintenance of genome integrity, meiosis, apoptosis, cell differentiation, signal transcription, and transduction. They are called gatekeepers since they are responsible for controlling cell growth [3]. According to Knudson’s two-hit theory, it is necessary to have two inhibitory mutations on two alleles of a gene to reduce or suppress its function, hence inhibiting the cell cycle and favoring tumor progression [13]. These genes are more susceptible to mutations in germinal cells rather than somatic cells, since the probability of having two mutations in the same locus of a gene in the same cell is low. Furthermore, they are commonly associated with family cancer syndromes such as retinoblastoma, where a first mutation (first hit) occurs in the first allele of the retinoblastoma (Rb) gene in germinal cells, and a second mutation (second hit) is acquired in the second allele of the Rb gene in somatic cells and is responsible for the loss of function of the Rb protein. Since Rb plays a crucial role in the control of cell cycle checkpoint at the G1 phase, a loss of its function induces a retinal tumor [3].

DNA repair genes, also called caretakers or guardians of genome integrity, are genes that detect and repair DNA damage. Mutations in these genes induce genetic instability that causes deletions, duplications, translocations, aneuploidies, and other chromosomal aberrations. Function loss of these genes is associated with different cancers such as familial breast cancer. This one result is due to a mutation in the DNA repair gene BRCA1 (Breast cancer gene 1) or BRCA2 (BRCA1); a loss of function of this gene induces an alteration of DNA repair and hence the development of cancer [14].

Carcinogenesis can be divided into three stages: initiation, promotion, and progression. Initiation is characterized by epigenetic modifications and genetic alterations. These alterations require cell division for their fixation and transmission to daughter cells. At this stage, cells are morphologically indistinguishable from other cells, and they remain quiescent in the organism [15]. During the promotion stage, the initiated cells grow clonally and develop into altered clones of cells characterized by a loss of growth control and immune evasion, and therefore to clinically detectable tumors [3]. Progression is the irreversible stage in which premalignant lesions develop into malignant cell populations or invasive cancer, due to the action of promoting agents that allow the propagation of cellular damage and the expansion of high heterogenic cellular clones. The high proportion of damaged cells leads to the production of more genetic alterations. In fact, genetic instability is one of the essential cancer hallmarks. Consequently, cells develop various capacities for metastasis, invasion, immune evasion, metabolism alteration, and resistance to death [3].

Epigenetic modification plays an important role in cancer development since it regulates the chromatin structure and gene expression, and determines how cells can respond to environmental signals in a coordinated way. The deregulation of epigenetic mechanisms due to external (e.g., toxins, nutrition, stress etc.) or internal (e.g., genetic, aging, etc.) factors can change the structure and stability of the genome, lead to mutagenesis, deregulate the expression of genes that are implicated in the maintenance of the cell cycle and alter chromatin remodeling at all cancer stages. Since epigenetic aberrations, unlike genetic mutations, are potentially reversible, reprogramming cancer epigenomes has become one of the promising target therapies for both cancer treatments and reversibility of drug resistance [16].

## 3. Epigenetic Modifications

**Figure 2:**
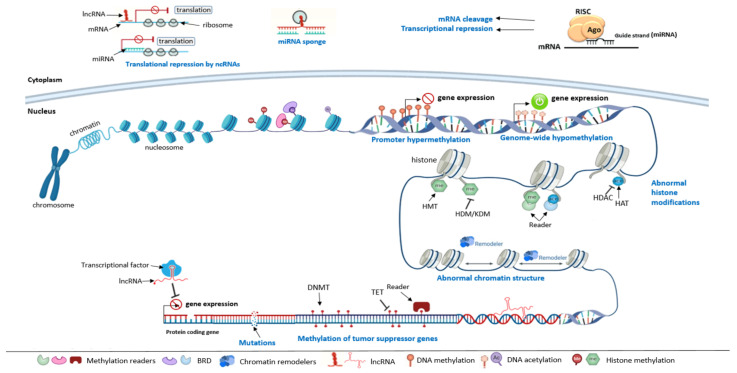
Epigenetic modifications. *Several epigenetic modifications can inhibit and/or activate gene expression. **First mechanism**: DNA methylation (inhibited gene expression) is catalyzed by DNMT enzymes and recycled by TET enzymes. This chemical modification is marked at the first time by de novo DNMT during cell differentiation and maintained by DNMT of maintenance during cell division. TET enzymes recycle DNA methylation by their capacity of demethylation. **Second mechanism**: Histone modifications (inhibit and/or activate gene expression) are mediated by several enzymes and molecular complexes, including HMT, HDM/KDM, HDAC, and HAT. **Third mechanism**: Remodelage of chromatin which is regulated by nucleosome positioning. Fourth mechanism: mRNA and ncRNA which control gene expression and repression via the upregulation of RNA translation.* Abbreviations: DNMT: DNA methyltransferases; HMT: Histone Methyl Transferase; HAT: histone acetyl transferase; HDM: histone demethylases; KDM: Histone Lysine Demethylases; HDAK: Histone Deacetylases.

Epigenetic modifications refer to the transmissible changes in gene expression and chromatin structure that occur without a physical change in the DNA sequence. These changes are heritable and reversible and are crucial for the maintenance of cellular identity and the regulation of vital cellular processes. Epigenetic modifications involve changes in chromatin structure, the methylation status of DNA fragments, and chemical transformations of histone chromosomal proteins (acetylation, methylation, ADP-ribosylation, ubiquitination, and phosphorylation), and the regulation of non-coding RNAs (ncRNAs). Among the altered ncRNAs, we can find long non-coding RNAs (lncRNAs) that are implicated in chromatin remodeling, transcriptional and post-transcriptional regulation [17], microRNAs (miRNAs) that mediate the post-transcriptional regulation of genes, the availability of a set of transcription factors, and the modification of the functional properties of proteins after the last stage of translation [14] (Figure 2).

Epigenetic modifications play different developmental and physiological roles in living organisms. In fact, it is responsible for cellular differentiation, mono-allelic expression of genes subjected to parental imprinting, and inactivation of the X chromosome in women. It regulates different cellular functions such as transcription, post-transcription, translation, RNA splicing, and nuclear export [14]. It also regulates the organizational states of chromatin and DNA packaging in the nucleus [14]. In addition, it modulates the functional and phenotypical changes as well as the gene expression from one cell type to another during the development stages through the establishment of specific brands, which explain the variety of gene expression in different cell types and tissues despite having the same genetic code. Furthermore, once differentiation is established, the epigenetic markers are stable enough to maintain cell identity during cell division. This maintenance is achieved in part by the maintenance enzyme, DNMT1, which maintains the DNA methylation on the hemi-methylated strands during mitosis [18]. However, epigenetic events are sufficiently reversible to allow state transitions.

Epigenetic modifications also play a crucial role in sex differences and sexual dimorphism in the brain. During uterine development, exposure to gonadal hormones changes the epigenetic marks and determines sexual dimorphism, brain differentiation, and sexual behavior in individuals. Based on gonadal hormones released during development, DNA methylation was shown to be one of the most observed sexually dimorphic epigenetic marks, suppresses masculinizing genes and maintains brain feminization. For instance, newborn female rats have higher levels of DNA methyltransferase Dnmt3a in the amygdala compared to males, and when exposed to testosterone during the perinatal period, Dnmt3a expression decreases and females exhibit a masculine behavior in adulthood [19]. Furthermore, X-inactivation and epigenetic marks may also contribute to gender differences in cancer risk and may also explain the male predominance across some cancer types. For example, six of the 783 non-pseudo-autosomal regions (PAR) X-chromosome genes (*ATRX*, *CNKSR2*, *DDX3X*, *KDM5C*, *KDM6*, and *MAGEC3*) are considered tumor suppressors and are frequently mutated in male tumors. In contrast, females that have a second copy of the X chromosome can compensate for any loss of function via X chromosome inactivation escape, which explains the reduced cancer incidence in females compared to males [20].

The X inactivation chromosome (XIC) is the mechanism that ensures dose compensation between female (XX) and male (XY) mammals. It is controlled and regulated by different epigenetic mechanisms, including histone remodeling, chromatin packaging, and DNA methylation. During early female development, one of the two X chromosomes in each cell is inactivated and females contain a mosaic of two cell populations, one expressing the paternal X chromosome and the other expressing the maternal X chromosome [17]. Inactivation begins at the X chromosome inactivation center (*Xic*: X-Inactivation Center) and is ensured by several regulators mainly the two long non-coding RNAs (lncRNAs) *Xist* and its antisense *Tsix* that are located in *Xic*. After receiving the signal that initiates random X-inactivation, *Xist* is transcribed from Xi (inactive X chromosome); it covers the entire Xi and recruits chromatin remodelers and epigenetic proteins to establish transcriptional silencing. In contrast, *Tsix* is silenced on the Xi and expressed on the Xa (active X chromosome); it negatively regulates the transcription of *Xist* on Xa. Xist RNA levels may influence the type of X inactivation [21]. Recent studies have shown that this mRNA is not only involved in maintaining a silent state of the inactive X chromosome, but its proper expression is critical in the genesis of tumors. Indeed, by performing Knock-Out of *Xist* in mice, authors demonstrated a massive development of blood cancers in females. These tumors are associated with genetic and epigenetic instability due to the reactivation of the inactive X chromosome [22].

Moreover, several studies have demonstrated that epigenetic alterations are one of the main mechanisms underlying many human diseases, including cancer, that experiences significant deregulation of all epigenetic components, which both affect protein expression regulation and the modification of their functions. This deregulation determines the epigenetic signature of cancer [23].

Epigenetic modifications are highly impacted by environmental factors such as dietary factors. Nowadays, the massive observed change in food habits and lifestyle may directly or indirectly modify our epigenome and raise the risk of having diet-related diseases and cancers. Several studies have shown that some food compounds may have a causative role in cancer development, such as heterocyclic amines (HCAs) produced by meat cooking; they are considered to be genotoxic compounds capable of inducing colon cancer [24]. However, other studies have shown that some compounds may also play a preventive role through epigenetic modifications, including DNA methylation and chromatin remodeling. For instance, certain dietary polyphenols such as epigallocatechin, resveratrol, genestein, and curcumin have been shown to prevent cancer development by remodeling chromatin and gene expression. Lycopene, hesperidin, carotenoids, ascorbic acid, some types of soluble fibers, polyunsaturated fatty acids, etc., have a protective role in cancer development. In addition, zinc, selenium, and folate have anticancer properties since they are involved in the DNA repair process. Furthermore, the consumption of vitamins and phytochemical compounds (vitamin D, folate, methionine, lycopene, genistein, resveratrol, caffeic acid, hesperidin) can upregulate DNA methylation in cancer cells [25]. Therefore, it is always recommended to have regular physical activity and healthy eating habits, including the consumption of fruits and vegetables, whole grains, legumes, nuts, seeds, and low-fat dairy products [26]. However, the risk of developing cancer varies among individuals due to the differences in their genome, epigenome, proteome, transcriptome, and metabolome, which contribute to the large fluctuation in tumor hazard among people [25].

Different enzymes and chromatin-associated proteins govern and control the epigenetic modifications, and are classified into three groups: writers, readers, and erasers. Writers refer to enzymes that transfer chemical groups to DNA or histone tails, including DNA and histone methyltransferases, acetyltransferases, kinases, and ubiquitinates. Erasers are enzymes that remove these chemical groups and include deacetylases, phosphatases, demethylases, and deubiquitinases. Readers are proteins that recognize the modified DNA or histone, such as bromodomain-containing proteins, methyl-lysine and methyl-arginine-binding domain-containing proteins, and PDH-containing proteins [27].

## 4. Implication of Epigenetic Perturbations in Cancer Diseases

### 4.1. DNA Methylation and Cancer

Different epigenetic modifications of DNA are responsible for gene regulation. Among them is DNA methylation, which induces the repression of gene expression. It can occur predominantly in repetitive genomic regions comprising satellite DNA and parasitic elements such as long interspersed transposable elements (LINES), short interspersed transposable elements (SINES), and endogenous retroviruses [28]. It can also occur in genes, including promoters, intra and intergenic enhancers, exons and introns. Additionally, it can occur in intergenic DNA and sequences that contain a high frequency of CG dinucleotide repeats called CpG islands (CGIs). It is estimated that about 60% of human genes contain promoters with CGIs, and the majority of these are unmethylated [29]. The methylation of CGIs induces transcriptional regression, and is strongly controlled by various concerted and/or inducible mechanisms [30].

**Figure 3:**
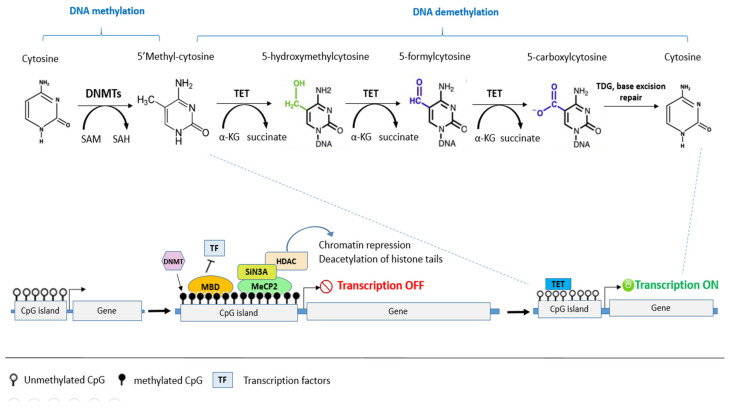
DNA methylation and demethylation. *Methylation of CpG islands promotor region can recruit repressive MBD and impair transcription factor binding. Moreover, methylation of CpG can recruit MeCP2 and therefore SIN3A which can recruit HDAC enzymes that deacetylase histones and therefore condense chromatin (heterochromatin state). In contrary, the demethylation of CpG islands is mediated by TET enzymes creating therefore an euchromatin state (reaction of transcription).* Abbreviations: DNMT: DNA methyltransferases; MBD: Methyl-CpG-Binding Domain; TF: Transcriptional Factors; MeCP2: Methyl CpG Binding Protein 2; SiN3A: SIN3: Transcription Regulator Family Member A; HDAC: Histone Deacetylases; TET ten-Eleven Translocation.

DNA methylation of the CGIs located in gene promoters induces gene silencing through the addition of a methyl group (CH3) by DNA methyltransferases (DNMTs) using a methyl donor S-adenosylmethionine (SAM) to the 5′ carbon of cytosine nucleotides adjacent to guanines (CpG dinucleotides), to form 5-methylcytosine (5mC) (Figure 3). Methylation is mediated by a family group of DNMTs composed of DNMT1, DNMT3A, and DNMT3B [29]. DNMT1 is the most expressed DNMT in normal cell tissues [31], and is responsible for the methylation of the unmethylated strand of the hemi-methylated DNA during replication and ensures the passage of epigenetic information between cell generations, whereas DNMT3A and DNMT3B catalyze de novo methylation [32].

Methylation has two modes of transcription repression: firstly, by masking the DNA and inhibiting the binding of transcription factors, and secondly by recruiting methyl-CpG binding proteins (MBPs) such as methyl binding protein (MBD1, MBD2, MBD3) and methyl CpG binding protein 2 (MeCP2) that can bind to methylated CpG sites, and induce gene silencing and chromatin repression by recruiting chromatin remodelers [32]. For example, MeCP2 binds to methylated CpG sites in DNA, then recruits a corepressor complex containing mSin3 and histone deacetylases (HDAC1/2). HDACs modulate chromatin structure, remove the acetyl group from acetylated lysines of histone proteins, and repress gene expression [33] (Figure 3).

Methylation plays an important role in physiological phenomena such as regulation during early development, inactivation of X chromosome in females, cell and tissue differentiation, chromosome stability, repression of transposable elements, and genomic imprinting. However, inappropriate methylation can also inhibit the expression of genes essential for the control of cell cycle and genome integrity, such as tumor suppressor genes and DNA repair genes, and it may also increase the probability of mutational events within genes that can lead to multiple human diseases including cancer [34]. Cancer cells have been shown to generally have a lower overall genome methylation rate than normal cells, with a radically altered methylation pattern [30], and the hypermethylation of normally unmethylated promoter CGIs or the global hypomethylation of DNA may be considered a hallmark of cancer [29].

Methylation abnormalities related to tumor transformation can be deduced via several mechanisms. Firstly, the methylated cytosines can spontaneously undergo and/or trigger deamination that transforms them into thymine, thus causing thymine mismatch with guanine, and eventual transition from cytosine to thymine, which generates punctual mutations due to these phenomena [30]. The second mechanism is the methylation of CpG islands located at the promoters of tumor suppressor genes or DNA repair genes. This methylation allows the recruitment of MBD proteins that act mainly on the organization of chromatin, thereby inhibiting the expression of these genes [35,36]. The third mechanism is related to the parental imprint. Genes subjected to mono-allelic expression of parent-specific origin may have their expression disturbed, which may contribute to tumor transformation. The last mechanism is related to the demethylation of repetitive and transposable elements, which induce genome instability and promote recombination in these regions during cell division. Indeed, these mobile elements are often subject to epigenetic modification, and when they are very mobile, they transfer with them the epigenetic marks within the genome [37]. Furthermore, it was demonstrated that DNA methylation of genes involved in apoptosis (e.g., p53, p73, Fas/Apo1/CD95, DAPK, Caspase-8) could be an important mechanism by which tumor cells prevent apoptosis. Hence, restoring the gene expression of these silenced genes could be a good strategy to reinstate apoptotic pathways and treat cancer [38].

The first gene methylation study was carried out on retinoblastoma tumors and showed that the retinoblastoma gene (*RB* gene), which is a tumor suppressor gene that regulates the cell cycle, is inactivated by hypermethylation [39]. Other studies have reported promoter hypermethylation in different breast cancer genes that are implicated in DNA repair (e.g., *BRCA1*), apoptosis (e.g., *BCL2*), metastasis (e.g., *TWIST*), regulation of cell transcription (e.g., *HOXA5*), cell adhesion (e.g., *CDH1*), and hormone-mediated cell signaling (e.g., *ERα/β*) [40]. Furthermore, different genes are hypermethylated in cancers such as *CDKN2A*, *hMLH1*, and *APC* in colorectal cancer (CRC); *p16INKa*, *RB1*, and *VHL1* in renal cell carcinoma [29]; *TET2*, *DNMT3B*, *IDH1*, *BRAF*, and *MYC* in prostate cancer [41]; and *MGMT* in colon, lung, lymphoid, and other tumors [42]. Additionally, it was confirmed that DNMT1 is a key factor in the aggressivity of triple-negative breast cancer (TNBC). Indeed, it induces hypermethylation in the promoter regions of estrogen receptor (ER), tumor suppressor genes, microRNAs, and epithelial markers involved in suppressing epithelial-mesenchymal transition, which induces metastasis and cancer proliferation [43]. In addition, overexpression of *DNMT1* or *DNMT3a* can induce a global DNA hypermethylation and silencing of DNA repair genes such as *hMSH2*, *ERCC1*, *XRCC1*, and *hOGG1* genes which induce an alteration of DNA repair and lead to genome instability [44].

DNA demethylation is regulated by the ten-eleven translocations (TET) family of proteins (TET1, TET2, and TET3) through removing the methyl group from 5-mC over different oxidation steps using α-KG cofactor. TETs oxidize 5-mC and catalyze the conversion of 5-mC to 5-hydroxymethylcytosine (5-hmC). This is a relatively stable intermediate substrate and is less prone to further oxidation by TET proteins than 5-Mc. Its oxidation can lead to two products; 5-formylcytosine (5-fC) and 5-carboxylcytosine (5-caC). These two molecules can be excised by thymine-DNA glycosylase (TDG) and eventually be repaired to an unmodified cytosine (Figure 3). Overexpression of *TET1* or *TET2* can cause a global decrease in 5-mC [45], and mutation affecting the TET2 enzyme are critical in tumor development and particularly in leukemia [46,47].

It was reported that mutations affecting isocitrate dehydrogenase IDH1/2 induce a loss of their normal catalytic activity, which is the production of α-KG by the decarboxylating isocitrate, and a gain of function by catalyzing the reduction of α-KG into 2-hydroxyglutarate (2-HG), which inhibits the activity of α-KG-dependent histone demethylases and TET 5-methylcytosine hydroxylases. This disturbance of marks gives rise to epigenetic instability that characterizes many cancers such as glioma and leukemia [48,49]. In addition to its inhibitory effects, 2-HG has shown major pleiotropic effects such as inhibiting of histone demethylases and generating of hypoxic environment that lead to tumor transformation by loss of cellular identity and oxidative stress [50]. Furthermore, it was demonstrated that mutations in genes affecting DNA methylation (e.g., *DNMT3A*) and demethylation (e.g., *TET2*) often cause the silencing of target genes and are found mostly in AML cases [51].

Hypomethylation induces chromosome instability and gene activation. It can also activate the aberrant expression of some proto-oncogenes and the reactivation of transposable elements (TE), retroviruses and DNA repeats (e.g., Alu sequences, LINE, satellite DNA, centromeric and epicentromeric tandem repeats) [52]. For instance, it was reported that there is a global DNA hypomethylation (GDHO) in epithelial ovarian cancer that induces increased chromosomal instability and altered copy number, which is associated with poor prognosis [53]. This GDHO has further been observed in leukocytes and has proved a potential association of glioma risk [54]. DNA hypomethylation can also play a tumor suppressor role (Figure 3). For example, hypomethylation of TE (LINE1 and endogenous retroviruses “ERV”) has been reported to induce activation of their expression and antiviral signaling which enhances the immune checkpoint blockade response in kidney cancer cell lines [55]. Additionally, hypomethylation was found in oncogene promoters such as MN/CA9 in human renal cell carcinoma [56], and multidrug resistance 1 (MDR1) in invasive ductal breast carcinoma [12]. It has also been found in IL-10 CGI, a cytokine that contributes to the oncogenic activation and inactivation of tumor suppressor genes in gastric cancer [57].

It was also demonstrated that epigenetic modifications can lead to genetic damage. For instance, promoter hypermethylation of DNA mismatch repair gene *hMLH1*, DNA alkyl-repair gene O(6)-methylguanine DNA methyltransferase (*MGMT*), detoxifier glutathione S-transferase P1 (*GSTP1*), and *BRCA1* may induce microsatellite instability, G to A transitions, steroid-related adducts, and double-strand breaks in DNA [58].

### 4.2. mRNA Methylation and Cancer

The first transcriptional modification discovered in higher eukaryotic mRNAs and the most abundant in mammalian mRNA is the N^6^ methyladenosine (m^6^A), which was observed in the adenines located at the sequences GAC or AAC [59], 3′ untranslated regions (3′UTRs) implicated in RNA stability, subcellular localization and translation regulation. Additionally, it was observed in evolutionarily conserved regions and particularly near the stop codons, and in the consensus sequence RRACH (R = G or A; H = A, C or U) [41,60]. This m^6^A methylation induces various mRNA modifications such as alternative splicing, translation, translocation, and degradation. In humans, methylation is established by two m^6^A methyltransferases (writers) including methyltransferase-like protein 3 (METTL3) and METTL14 [61]. METTL14 is an RNA-binding protein that forms a stable heterodimer with METTL3 and strengthens the catalytic activity of METTL3. METTL3 induces methylation of RNAs in the nucleus by adding a methyl group (CH3) on RNA’s adenine using a methyl donor S-adenosylmethionine (SAM). Wilms tumor 1-associated protein (WTAP) is a protein that interacts with both METTL3 and METTL14 and allows their localization in nuclear speckles enriched with pre-mRNA processing factors [62]. Other regulatory factors can bind to the catalytic complex such as vir-like m^6^A methyltransferase associated (VIRMA or KIAA1429) that induces methylation near the stop codon and in 3′UTR; RNA-binding motif protein 15/15B (RBM15/RBM15B) that induces gene silencing of lncRNA xist implicated in X inactivation; and zinc finger CCCH domain-containing protein 13 (ZC3H3) that regulates m^6^A methylation in embryonic stem cells [41]. M^6^A can be removed by m^6^A demethylases (erasers) such as fat-mass and obesity-associated protein (FTO) that reduces m^6^A levels and α-ketoglutarate-dependent dioxygenase alkB homolog 5 (ALKBH5) that removes the m^6^A of the nuclear RNAs and hence modulates the nuclear RNA export, metabolism and gene expression [41]. The regulation of RNA methylation is controlled by different reader proteins such as the YT521-B homology (YTH) domain family, heterogeneous nuclear ribonucleoproteins (HNRNPs), and insulin-like growth factor 2 mRNA-binding proteins (IGF2BPs) [41]. Several YTH proteins have been identified, such as YTHDF2 that promotes mRNA degradation and YTHDF1 that enhances mRNA translation [41]. Some of these proteins are thought to be associated with several cancers. Indeed, it has been shown that YTHDF2 is associated with breast cancer since it can recognize the m^6^A sites of the BNIP3 mRNA that encodes for an apoptotic protein which induces cell death, and causes its degradation, hence the promotion of tumor growth. Otherwise, in hepatocellular carcinoma (HCC), YTHDF2 acts as a tumor suppressor and promotes the degradation of epidermal growth factor receptor (EGFR) mRNA, and negatively modulates its stability [63]. In addition, it was reported that hypoxia; which is a common phenomenon in the majority of malignant tumors that deprives cells of oxygen and promotes vascularization, metastasis, and metabolism deregulation; induces hypoxia inducible factor (*HIF*) expression [64]. This reduces *YTHDF2* expression in HCC cells and therefore decreases the degradation of m^6^A-containing IL-11 and serpin family E member 2 (SERPINE2) mRNAs which are crucial factors in inflammation-mediated malignancy and vascular remodeling, and therefore enhances inflammation, angiogenesis, and metastasis [65].

### 4.3. Histone Modification and Cancer

Histones are structural proteins that interact with DNA to create a miracle topology and allow DNA packaging in the nucleus as chromatin. This chromatin is arranged in repeating units called nucleosomes, each one is composed of an octamer of histone proteins formed by two copies of the four histone proteins (H2A, H2B, H3, and H4) surrounded by a 147 bp of DNA, and connected by a short DNA linker [66]. A linker histone H1 binds to the entry/exit sites of DNA on the nucleosomal core to consolidate the nucleosome binding with DNA and stabilize the architecture of higher-order chromatin [67]. This complex is the target of many types of regulation that ensure the release state of DNA as actively transcribed euchromatin, or the compacted state of DNA as transcriptionally inactive heterochromatin. It controls the accessibility of DNA and therefore regulates its transcription, replication, recombination, and repair [66].

Histone modification can occur in the N-terminal tails as well as in the core domain in response to various intrinsic and extrinsic changes that may occur throughout the life cycle. Histone tails are enriched with basic lys/arg and hydroxyl group-containing Ser/Thr/Tyr residues. They are more accessible and undergo many covalent posttranscriptional modifications (PTM), including acetylation and methylation of lysines (K) and arginines (R), phosphorylation of serines (S) and threonines (T), ubiquitylation, and sumoylation of lysines [45]. These PTMs modulate the interactions between DNA and histones; modify the acceptability of DNA towards transcription factors and restriction enzymes, and influence nucleosome positioning and stability [68]. PTMs on the histone core are less accessible since they are introduced on the histones before assembly into nucleosomes, as with H3K56ac and H4K91ac. However, chromatin remodelers can mediate the addition or removal of these core PTMs [68]. Moreover, histone modifications also include citrullination, ADP-ribosylation, deamination, formylation, O-GlcNAcylation, propionylation, butyrylation, crotonylation, and proline isomerization at over 60 amino acid residues [45].

#### 4.3.1. Histone Acetylation and Cancer

**Figure 4:**
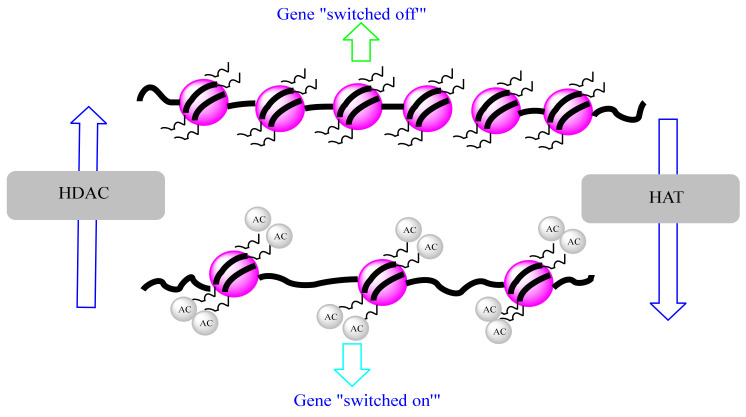
Histone modifications. *Gene expression can be is switched between repression and expression according to histone modifications. HDAC (histone deacetylase) induces a repression state (chromatin condensed), while HAT (histone transferase) induces an expression state (chromatin decondensed)*.

Histone acetylation and deacetyation are a dynamic process that is regulated by two family groups of enzymes: histone acetyl transferases (HAT) and histone deacetylases (HDAC) (Figure 4). HATs are divided into three groups, GCN5-related N-acetyl transferases (GNAT), MYST, and p300/CREB binding protein (CBP) [69]. They catalyze the transfer of an acetyl group from acetyl-CoA cofactor to the ε-amino group of lysine residues at histone proteins to neutralize their basic charge. Consequently, histone tails reduce their affinity to DNA, and chromatin becomes relaxed, accessible to transcription factors, and transcriptionally active [69]. In contrast, acetylated histone-lysine residues are recognized by Bromodomain and extra terminal domain (BET) proteins, including BRD2, BRD3, and BRD4 that recruit HDAC proteins. These deacetylases remove the acetyl group from histone lysine and reestablish the positive charges of histone lysine residues, increase the interaction between histones and DNA, and induce DNA compaction and transcriptional repression [70]. HDAC family comprises 18 enzymes that can be grouped into four classes: class I comprises HDAC 1, 2, 3, and 8 that are located in the nucleus; class II found in both the nucleus and cytoplasm and is divided into class-IIa that contains HDAC 4, 5, 7, and 9, and class-IIb that includes HDACs 6 and 10; class III comprised of nicotinamide adenine dinucleotide-dependent SIRT [sirtuin] enzymes (SIRT1, SIRT2, SIRT3, SIRT4, SIRT5, SIRT6, and SIRT7), and class IV involving HDAC 11 [71]. HATs and HDACs not only target histone proteins, but they can also interact with non-histone proteins, including transcription factors and proteins involved in DNA repair, cellular signaling, metabolism, cytoskeletal dynamics, apoptosis, nuclear import, and protein folding [72]. HDACs are implicated in various functions other than gene expression control, including protein stability, protein translocation, enzymatic activity, protein-protein interaction, and DNA binding affinity via acetylation of non-histone proteins. They can also regulate gene transcription by deacetylating other epigenetic proteins such as DNMTs and HATs [45].

Both HAT and HDAC play important roles in maintaining chromatin accessibility and regulating gene expression. However, an alteration in this regulation may lead to the emergence of different diseases such as cancer. Different studies have shown that these enzymes are associated with cancer promotion and progression, and many inhibitory drugs (HATi and HDACi) were used as therapies to inhibit the activity of these enzymes and to treat cancer. Mutations in HAT genes are implicated in many different cancers, whereas that of HDACs seems to have been implicated so far in some leukemia cancers [73]. HDAC1 has been reported to be overexpressed in prostate cancer cells [74], and it is also responsible for the upregulation of the long non-coding RNAs BC01600 and AF116637 which induce the promotion of cell proliferation in gastric cancer [75]. HDAC2 is overexpressed in human lung cancer cells and deregulates the expression of apoptotic and cell cycle proteins [76]. It is also overexpressed in breast cancer [77,78] and human gastric carcinomas [79]. In addition, HDACs can deacetylate non-histone proteins involved in different cellular processes such as differentiation (e.g., MyoD, MEF2), autophagy (e.g., Atg5, Atg7, Atg8), apoptosis (p53), DNA damage repair (e.g., WRN), and immune responses (e.g., STAT3). Therefore, aberrant deacetylation of these proteins may be implicated in cancer development. It has also been shown that genes involved in DNA demethylation (e.g., *DNMT3A*, *TET2, IDH1*, and *IDH2*) or histone methylation and demethylation (e.g., *EZH2*, *MLL*, and *DOT1L*) are frequently mutated in primary and secondary AML. Furthermore, some histone demethylases, such as LSD1, are frequently overexpressed in AML [70].

#### 4.3.2. Histone Methylation and Cancer

As noted above, histone methylation consists of three components; writers (Histone Methyl Transferases: HMTs), readers (Histone Methylation Recognizing Proteins), and erasers (Histone Demethylases: HDMs). It can occur in arginine (R) and lysine (K) and is catalyzed by HMTs (KMTs or RMTs), that transfer the methyl group from S-adenosyl-1-methionine (SAM) to R and K residues. K residues can be mono-, di- or tri-methylated, while R residues can only be mono- or di-methylated [45]. Histone methylation can promote or inhibit gene expression. For instance, methylation of histone H3K4, H3K36, and H3K79 is associated with transcriptional activation, while di- and tri-methylation of H3K9 and H3K27 are associated with transcriptional repression [70]. Histone demethylation is catalyzed by two HDMs; amine oxidase type lysine-specific demethylases (LSDs or KDM) that remove methyl group only from mono- and di-methylated lysines, and JumonjiC (JMJC)-domain-containing histone demethylases that demethylate the three-methyl states in lysine and arginine residues [70].

Alteration of histone methylation can induce a perturbation in genes expression, which can lead to the development of various diseases such as cancer. For instance, mutations within *EZH2*, a histone methyltransferase that mediates repression of gene transcription via *H3K27*, have been reported to be associated with diffuse large B-cell lymphoma (DLBCL) [80].

### 4.4. Chromatin Remodeling

Chromatin remodeling is a vital process that is evolutionarily conserved from yeast to humans [81]. It ensures genome packaging and unpackaging and regulates accessibility to DNA regulatory elements that control chromosomal processes (enhancers, promoters, replication origins) to regulate gene transcription, DNA replication, repair and recombination. This process implicates different remodelers. Four families of chromatin remodeling complexes were identified, including switching defective/sucrose non-fermenting (SWI/SNF) family, imitation switch (ISWI) family, chromodomain, helicase, DNA binding (CHD) family, and Inositol requiring 80 (INO80) family. All of these families use the energy of ATP hydrolysis to change chromatin structure and regulate transcription machinery proteins by reorganizing nucleosomes. They all have the same properties, including nucleosome affinity, domains that recognize covalent histone modifications, similar DNA-dependent ATPase domain, domains and/or proteins that regulate the ATPase domain, and domains and/or proteins that interact with other chromatins or transcription factors [82]. Each ATPase family catalyzes distinct functions, including eviction or change of the histone octamer or subunits, creation of DNA loops on nucleosome surface, and incremental nucleosome sliding on DNA in cis [83]. Mutations in these families of remodelers can lead to abnormal cell development that can induce cancer.

The SWI/SNF family remodelers modify chromatin accessibility through chromatin repositioning, nucleosome sliding and ejection, and histone dimer eviction (H2A/H2B dimers) from nucleosome structure. They play crucial roles in several biological processes, including DNA differentiation, proliferation, and repair. They also control transcription activation or repression through exposing or concealing binding sites of gene promoters or enhancers [84]. SWI/SNF family remodelers are mutated in more than 20% of human cancers. For example, the BAF chromatin-remodeling complexes, which include BRG1 (SMARCA4) and BAF250a (ARID1A) that regulate transcription through the control of chromatin structure and the placement of polycomb repressive complex 2 (PRC2) across the genome are mutated in different cancer types. *Brg1* has been reported to be mutated in prostate, lung, and breast cancer cell lines, and is considered to be a tumor suppressor gene in certain cancer types [82]. A deletion of this gene induces anaphase bridge formation and a G_2_/M phase arrest. BAF complexes interact with and activate endogenous topoisomerase IIα (TOP2A) via BAF250a to prevent DNA entanglement during mitosis. Mutations within its subunits lead to inactivation of TOP2A, which induces genome instability and tumorigenesis [85].

The ISWI family remodelers catalyze the sliding of nucleosomes and optimize their spacing to promote chromatin assembly and limit chromatin accessibility and gene expression. They also play various functions, including the spacing of nucleosomes after DNA replication, coregulation of transcription and RNA polymerase elongation, and regulation of DNA damage repair [83]. There are seven mammalian ISWI complexes that have been described and include ACF, NURF, NoRC, WICH, RSF, CHRAC, and CERF. Each contains an ATPase subunit SMARCA5 (SNF2H) or SMARCA1 (SNF2L), and one or more accessory subunits [86]. Different ISWI complexes are deregulated in cancers. For instance, BPTF, a core and largest subunit of NURF complex, was highly expressed in HCC and was correlated with advanced malignancy and poor prognosis in HCC patients. It plays a key role in the regulation of the expression of human telomerase reverse transcriptase (*hTERT*) which provides telomere synthesis, cell immortality, tumor growth, and metastasis [63]. It is also overexpressed in non-small-cell lung cancer (NSCLC) and induces cell growth and survival through the transcriptional activation of vascular endothelial growth factor (VEGF) that activates various signaling pathways including mitogen-activated protein kinase (MAPK) and PI3K [87]. In addition, BPFT has been reported to cooperate with P50 NF-κB to regulate the expression of COX2 that promotes lung cancer development [88]. Furthermore, it inhibits the antitumor activity of natural killer (NK) cells through activating heparanase expression, which reduces cell surface heparan sulphate proteoglycans (HSPGs) and natural cytotoxicity receptor (NCR) co-ligands that are essential for NK cells [89].

CHD family remodelers contain different subgroups due to the diversity of their chromodomain types. They are implicated in all remodeling processes, including nucleosome assembly, chromatin accessibility, and editing (incorporation of histone H3.3) [84]. They may play a transcriptional activation role by siding or ejecting nucleosomes, or a transcriptional repression role such as vertebrate Mi-2/NuRD that contains HDAC1/2 and MBD proteins and induces chromatin repression [82]. Moreover, one of the most characterized complexes of this family is NURD, which contains both ATP-dependent chromatin remodeling, including the chromodomain-helicase-DNA-binding protein 3 (CHD3) and CHD4 and histone deacetylase activities (HDAC1/2) playing enzymatic functions. It also includes other non-enzymatic subunits such as MBD2, MBD3, metastasis-associated gene 1 (*MTA1*), *MTA2*, *MTA3*, and retinoblastoma binding protein 4 and 7 (RBBP4/7) [90]. These non-enzymatic subunits confer the functional specificity of the NuRD complex. For instance, MBD3 is enriched at active promoters, while MBD2 is enriched at exon sequences of active genes and it can bind to 5mC DNA and promote gene repression through its remodeling and histone deacetylase activities [91]. Different subunits were reported to be associated with various cancer types. Indeed, MAT1 has been reported to promote breast cancer progression and metastasis by altering signaling pathways [92], including the activation of *STAT3* transcription [93] and repression of *SMAD7* transcription to ensure the TGFβ signaling [94].

The INO80 family remodelers perform editing functions, including catalysis of the exchange of histones from the nucleosome structure and the promotion of nucleosome repositioning. They regulate gene transcription [95], DNA repair, checkpoint regulation, replication and genome integrity [96], and are implicated in telomere regulation and chromosome segregation. INO80 remodelers can be divided into two classes; INO80-C (canonical) comprising RUVBL1, RUVBL2, MCRS1, YY1 subunits and ensure chromatin opening and accessibility with H3K27ac P300; and INO80 ATPase (non-canonical) providing chromatin repression [97]. INO80 dysfunction may deregulate DNA synthesis, gene expression and DNA repair, which may lead to genome instability and tumorigenesis. In fact, INO80 has been reported to be able to establish and maintain an open chromatin state at enhancers to promote oncogene expression and tumor growth in NSCLC [98]. It also activates the super-enhancer-mediated oncogenic transcription that enhances oncogenes expression and tumor growth in melanoma [99]. INO80 has also been found to be overexpressed in cervical cancer and has shown the ability to promote carcinogenesis through binding and activating the Nanog transcription start site (TSS) and improving the development of cervical cancer cells [100]. Furthermore, other subunits of this family were associated with different cancers. For instance, *RUBVL2* is overexpressed in HCC. It promotes carcinogenesis through activating the heat shock protein 90 (HSP90)-Cell Division Cycle 37 (CDC37), AKT serine/threonine kinase (AKT), and (ERK/MAPK) pathways [101]. Additionally, *EED*, *ARID1A*, *ING5*, *CBX3*, *CBX7*, and *MTA1* chromatin remodeling genes are involved in the epigenetic mechanisms of the gastrointestinal carcinogenesis (GC) and have key roles in disease initiation and progression and may also be promising markers for GC screening [102].

### 4.5. RNAs Modification and Cancer

The human genome is composed of coding sequences that represent the minority of the total genome size and non-coding sequences that present the majority of DNA sequences and are considered junk DNA. These include pseudogenes, transposon elements, repeated non-coding sequences (e.g., telomeres), regulatory elements (e.g., promoters, enhancers, silencers, insulators), non-coding genes, etc. Only about 1–2% of genetic transcripts can encode proteins, and over about 98% of genetic transcripts encode non-coding RNAs that play an important role in regulating gene expression [103]. Several ncRNAs have been described such as micro RNA (miRNA), transcribed ultraconcerved regions (T-UCR), small nucleolar RNA (sno-RNA), PIWI-interacting RNA (piRNA), large intergenic non-coding RNA (lincRNA), and long non-coding RNA (lncRNA) [104]. However, miRNAs and lncRNAs are the most studied.

#### 4.5.1. MicroRNAs and Cancer

MicroRNAs (miRNAs) are small non-coding RNAs of about 18–25 nucleotides (nt) in length. They are located in non-coding genomic regions and intronic or exonic regions of protein-coding genes [105]. They are also frequently located at fragile sites, as well as in minimal regions of amplification, minimal regions of loss of heterozygosity, or common breakpoint regions [106]. They regulate gene post-transcription and induce translational repression and mRNA degradation. They can also target promoter sequences and activate gene expression [107]. Moreover, miRNA molecules are highly conserved and have a crucial role in many biological processes including proliferation, differentiation, cell cycle regulation, apoptosis, development, stress response, etc. [105]. They are transcribed in the nucleus by RNA polymerase II or III to primary miRNAs (pi-miRNAs). These pi-miRNAs are then polyadenylated, capped, and cleaved in the nucleus by a complex involving drosha ribonuclease and DiGeorge syndrome critical region gene 8 (DGGR8), forming a hairpin RNAs known as a precursor miRNA (pre-miRNA). Pre-miRNAs are exported to the cytoplasm by exporting-5 and cleaved by a ribonuclease Dicer into an RNA duplex containing two miRNA strands. The first strand is degraded and the second one is stably associated with the RNA induced silencing complex (RISC) and binds to the 3′-untranslated region (3′-UTR) of the targeted messenger RNA (mRNA) through sequence complementarity and forms a duplex. The RISC complex can then represses mRNA expression through mRNA destabilization, degradation, cleavage, and mRNA translational inhibition [108].

These miRNAs are implicated in epigenetic machinery and can regulate gene expression by recognizing specific sites in gene promoters or by recruiting other epigenetic regulators. However, they can also be epigenetically modified. The activation or repression of their expression is modulated by different epigenetic mechanisms, including histone modifications (e.g., histone acetylation or deacetylation), DNA methylation (e.g., hypo or hypermethylation of miRNA gene promoter), etc. Deregulation of this complex system can result in various pathologies, including cancer. miRNAs may act as a tumor suppressor and their downregulation or silencing by epigenetic modifications (CpG island hypermethylation, or chromatin remodeling) can cause cancer development and increased malignancy. They can also act as oncogenes and promote cell proliferation, invasion, and metastasis [105]. The miRNAs (miR)-29 family (29a, 29b, and 29c), which have an intriguing complementarity with the 3’-UTRs of DNMT-3a and -3B, have been shown to be downregulated in lung cancer, which led to elevated expression levels of DNMT-3a and -3B associated with a poor prognosis. However, the enforced expression of miR-29s was shown to down-modulate expression levels of DNMT3A and -3B in lung cancer, which reduces global DNA methylation, restores expression of tumor suppressor genes such as Fragile histidine triad diadenosine triphosphatase (FHIT) and WW domain-containing oxidoreductase (WWOX), and inhibits tumorigenicity (in vitro and in vivo) [109]. Furthermore, miR-21, an important regulator of tumor growth, migration, and invasion, has been reported to be overexpressed in a wide variety of solid tumors, namely human hepatocellular cancer (HCC), breast, colon, lung, pancreas, prostate, stomach, and cholangiocarcinoma cell lines [110]. In addition, it was demonstrated that a high miR-21 expression in HCC represses the expression of phosphatase and tensin homolog (PTEN) tumor suppressor and increases the activity of AKT and mammalian target of rapamycin (mTOR) kinase pathways, which promotes cell proliferation and survival. However, inhibition of miR-21 in HCC decreases tumor proliferation, migration, and invasion by repressing the expression of PTEN and its downstream effects. Moreover, miR-21 represses the expression of the programmed cell death 4 (PDCD4) tumor suppressor protein in breast cancer cells [111]. Other miRNAs are implicated in the activation of cancer development such as miR-19 and miR-501-5p through activating wingless (WNT)/β-catenin signaling pathway, and miR-483-5p, miR-196b-5p and miR-494-3p through activating the cyclin D1, STAT3, and Notch1 signaling pathways. Contrarily, some miRNAs inhibit cancer development such as miR-195-5P and miR-34 by inhibiting the Notch1 pathway, miR-99a through inhibiting the Mtor pathway, miR-519d and miR-128 by activating caspases and inducing apoptosis [112].

#### 4.5.2. Long Non-Coding RNAs and Cancer

Long non-coding RNAs (lncRNAs) are transcripts about 200 nt to 100 kilobases pair (kbp) long that do not encode proteins [113]. The majority are synthesized by RNA polymerase II under the control of the transcriptional activators of the SWI/SNF complex or by RNA polymerase III. They interact with DNA, RNA molecules or proteins and they account for the majority of the genome transcripts. LncRNAs are poorly annotated; however, several studies have demonstrated that they play a crucial role in various physiological processes such as development, differentiation, and proliferation. They are also implicated in chromatin remodeling, transcriptional and post-transcriptional regulation, splicing regulation, X chromosome inactivation, and genomic imprinting [113,114]. Deregulation of lncRNAs may induce various human diseases, including cancer.

LncRNAs can mediate epigenetic modification by recruiting chromatin-remodeling complexes at a specific chromatin locus. A model example of this process is the XIST locus that controls the X chromosome inactivation as described above [115]. There is also the Hox transcript antisense RNA (HOTAIR), a lncRNA that is encoded by the human homeobox C (HOXC) locus. It inhibits transcription in trans of across 40kb of the HOXD locus by binding and regulating the function of chromatin remodeling PRC2 which is comprised of H3K27 histone methyl transferase, enhancer of zeste homolog 2 (EZH2) and core components Suz12 and EED, hence inducing repression of the chromatin state [116]. lncRNAs are also considered as cofactors that modify the activity of transcription factors or as molecular ligands that can recruit specific RNA-binding proteins to gene promoters [113]. For instance, when DNA is damaged, the long ncRNAs associated with the cyclin D1 (CCND1) gene promoter are stimulated and they activate the function of RNA binding protein TLS (translocated in liposarcoma) that causes silencing CCND1 expression by inhibiting the HAT functions of CREB binding protein (CBP) and p300 and hence inhibit the cell cycle [117]. Furthermore, they regulate mRNA post-transcriptional processes, in particular their translational repression and their degradation by base pairing [118].

LncRNAs play a significant role in cancer development and progression and they are implicated in all cancer hallmarks [1]. They may act as tumor suppressors or oncogenes and deregulation of their expression can launch cancerization processes. It was reported that lung cancer-associated transcript 1 (LUCAT1) is significantly related to the development and progression of esophageal squamous cell carcinoma (ESCC) and is implicated in the regulation of DNMT1 expression and ubiquitination by UHRF1 (Ubiquitin-like with PHD and Ring Finger domains 1) an epigenetic regulator of DNMT1. Therefore, it favors the expression of DNMT1 that represses the expression of tumor suppressor genes and leads to the development and progression of ESCC [119].

Furthermore, the metastasis-associated lung adenocarcinoma transcript 1 (MALAT1) has been reported to be associated with metastatic potential, and it was found to be upregulated in many cancers. It enhances cell motility of lung adenocarcinoma cells through regulating the motility-related genes such as collagen triple helix repeat containing 1 (CTHRC1), chaperonin-containing tailless complex polypeptide, subunit 4 (CCT4), hyualuronan-mediated motility receptor (HMMR), and regulator of differentiation 1 (ROD1) via transcriptional and/or post-transcriptional regulation [120]. It also promotes epithelial ovarian cancer cell survival and progression by acting as a sponge for miRNAs [121]. It acts as a sponge for miR-22 and counteracts its inhibitory effect on c-myc-mediated epithelial-mesenchymal transition [122].

Maternally expressed gene 3 (MEG3), an lncRNA that is associated with tumorigenesis, is considered a tumor suppressor since it induces cell growth arrest and promotes cell apoptosis. However, it was found that hypermethylation of its promoter or its differentially methylated regions (DMRs) induces a loss of its expression and activity, and hence, promotes tumorigenesis and cell proliferation in gastric cancer [123]. Taurine-upregulated gene 1 (TUG1) is upregulated in bladder urothelial carcinoma and it promotes proliferation and migration of ESCC cells [124,125]. ANRIL promotes the proliferation and migration of prostate cancer cells through the regulation let-7a/TGF-β1/Smad signaling pathway that regulates the proliferation, migration, and epithelial-mesenchymal transition (EMT) of prostate cancer [126]. KCNQ1OT1 promotes CRC cell proliferation by increasing aerobic glycosis through binding and stabilizing the glycolytic enzyme hexokinase 2 (HK2). This mechanism is known as the Warburg effect, which allows for increased glucose uptake, and rapid production of ATP to accelerate cells growth and proliferation [127]. Other lncRNAs were reported to be associated with different cancers such as LINC00152 (osteosarcoma, gastric cancer, NSCLC, etc.) [128,129,130], RP11-89K21.1 and RP11-357H14.17 (endometrial carcinoma) [131], ADAMTS9-AS2 (liver cancer) [128], SUMO1P3 (glioma) [132], and OIP5-AS1 (ovarian cancer) [128].

## 5. Natural Compounds as Epidrugs against Cancer

### 5.1. Flavonoids Targeting Epigenetic Pathways in Cancer

Flavonoids present a remarkable epidrugs candidate against different cancer cell lines. Table 1 summarises investigated studies about anticancer effects of different flavonoids with epigenetic modifications (Table 1).

#### 5.1.1. Anthocyanidins

Anthocyanidins are the sugar-free equivalents of anthocyanins, which are common plant pigments. They are based on the flavylium cation, an oxonium ion with different classes substituting for the hydrogen atoms. As a feature of pH, they commonly shift color from red to purple, blue, and bluish green [133].

#### 5.1.2. Anthocyanins

To reveal the causal demethylation effects observed in colorectal cancers treated with black raspberries, Zhang et al. [134] conducted a study specifically using anthocyanins. After three days of treatment, anthocyanins suppressed the activity and protein expression of DNMT1 and DNMT3B in HCT116, Caco2, and SW480 cell lines. Indeed, the promoters of CDKN2A, and SFRP2, SFRP5, and WIF1, upstream of the Wnt pathway, were demethylated by anthocyanins. The mRNA expression of some of these genes increased while mRNA expression of β-catenin and c-Myc, downstream of the Wnt pathway, and cell proliferation decreased. Moreover, anthocyanins were taken up in HCT116 cells and differentially localized with DNMT1 and DNMT3B in the same cells visualized using confocal laser scanning microscopy. Although DNMT3B has been reported to be regulated by c-Myc in mouse lymphoma, DNMT3B did not bind to c-Myc in HCT116 cells. These results suggest that anthocyanins are responsible for the demethylation effects of whole black raspberries in colorectal cancers.

#### 5.1.3. Cyanidin

Cyanidin, a plant pigment, is present in many food sources such as corn as cyanidin 3-glucoside and its acylated derivatives [135]. Its consumption was reported to reduce myocardial damage during ischemia-reperfusion and against the cardiotoxic effects induced by Doxorubicin. It is a potent chemopreventive agent [136]. Paluszczak et al. [137] demonstrated the role of cyanidin on the activity and expression of DNMTs in the human breast cancer MCF7 cell line, as well as its impact on DNA and histone H3 methylation. This is because cyanidin inhibited DNA methyltransferase without affecting the methylation pattern or expression of RASSF1A, GSTP1, or HIN1 or the methylation of histone. This suggests that non-nucleoside agents are probably not effective epigenetic modulators. However, according to this study, a long-term exposure to these chemicals in diet might potentially lead to an effect, which can be sufficient for cancer chemoprevention.

#### 5.1.4. Pelargonidin

Pelargonidin, an anthocyanidin reported to be present in *Prunus domestica* Africana (the Peruvian cherry) and has various documented biological activities [138]. Its epigenetic potential was investigated [139,140].

Li et al. [140] investigated the impact of pelargonidin on cellular transformation caused by the tumor promoter 12-O-tetradecanoylphorbol-13-acetate in mouse skin epidermal JB6 (JB6 P+) cells (TPA). Pelargonidin therapy reduced colony development and strongly inhibited the viability of JB6 P+ cells, overexpressing the ARE-luciferase reporter in HepG2-C8 cells and activating the antioxidant response element (ARE)-luciferase. Pelargonidin decreased the protein levels of genes encoding DNMTs and HDACs. Importantly, this compound reduced DNA methylation in the nuclear factor erythroid 2-related factor 2 (Nrf2) promoter area of JB6 P+ cells, thereby increasing the expression of Nrf2 downstream target genes such as NAD(P)H/quinone oxidoreductase 1 (NQO1) and heme oxygenase-1 (HO-1) [140]. In addition, pelargonidin inhibited TPA-induced cell transformation. According to the findings of this study, activation of the Nrf2-ARE signaling pathway and its cytoprotective function are two plausible molecular mechanisms for pelargonidin [140].

Karthi et al. [139] looked into the molecular mechanism of pelargonidin’s activity on cell cycle regulators CDK1, CDK4, and CDK6, as well as the substrate-binding domains of DNMT1 and DNMT3A, which control DNA methylation epigenetic signals. Docking study, binding free energy calculations, and molecular dynamic simulations were all in agreement with the experimental findings, indicating that pelargonidin has a particular relationship with CDK1. Pelargonidin can also inhibit DNA identification and catalytic binding by DNMT1 and DNMT3A in this sense. Pelargonidin functional groups were mapped using the HOMO-LUMO method. The study indicates that this compound can be used as a multitarget agent for cancer therapy, according to pharmacological descriptor predictions [139].

#### 5.1.5. Delphinidin

Delphinidin is a pigmented vegetable and fruit anthocyanidin found in abundance in pigmented vegetables and fruits, especially blueberries, and is known for its antioxidant and anti-inflammatory effects [141]. Delphinidin has been shown to have antioxidant, anti-inflammatory, and antimutagenic effects, and has been shown to inhibit breast cancer cell invasion. On in vitro and in vivo models, delphinidin has been shown to have antiangiogenic properties [142].

Based on the role of redox imbalance in the pathogenesis of melanoma and nonmelanoma skin cancer, Kuo et al. [143] were interested in flavonoids (anthocyanidins), which have been shown to activate Nrf2 antioxidant responsive element (ARE) pathway as an intrinsic defense mechanism against oxidative stress. Delphinidin, one of the most powerful and abundant anthocyanidins found in berries, suppressed 12-O-tetradecanoylphorbol13-acetate (TPA)–induced neoplastic cell transformation in mouse epidermal JB6 P+ cells by 69.4 to 99.4%. The demethylation of 15 CpG sites in the mouse Nrf2 promoter region between nt 1226 and 863 from the transcription start site was linked to the activation of the Nrf2-ARE pathway. The reduction in protein expression of DNMT1, DNMT3a, and class I/II histone deacetylases was consistent with the lower CpG methylation ratio in the Nrf2 promoter area (HDACs). Overall, these findings demonstrate that delphinidin, an epigenetic demethylating agent of the Nrf2 promoter, may activate the Nrf2-ARE pathway, suggesting that it might be used as a skin cancer chemopreventive agent.

**Table 1:** Flavonoids as epidrugs.

Molecule (Origin)	Used Model	Key Results and Conclusion (⇒)	Refs.
**Anthocyanidins**			
Anthocyanins (Raspberry)	Colorectal T116, Caco2, and SW480 cells	- Suppressed the activity and protein expression of DNMT1 and DNMT3B in HCT116, Caco-2, and SW480 cells - Demethylated the promoters of CDKN2A, and SFRP2, SFRP5, and WIF1, upstream of Wnt pathway - Increase expression of mRNA of certain genes - Decreased mRNA expression of β-catenin and c-Myc and cell proliferation - Increased apoptosis ⇒ Anthocyanins are responsible, at least in part, for the demethylation effects of whole black raspberries in colorectal cancer	[134]
Cyanidin (Not reported)	MCF7 breast cancer cells	- Inhibited the DNMT activity - No effect on the methylation pattern or the expression of *RASSF1A*, *GSTP1* or *HIN-1* in MCF7 cells ⇒ Cyanidin is unlikely to be effective epigenetic modulator	[137]
Delphinidin (Purchased)	Mouse epidermal JB6 P+ cell line Human hepatocellular HepG2-C8 cell line	- Inhibited 12-O-tetradecanoylphorbol- 13-acetate (TPA)–induced neoplastic cell transformation by 69.4 to 99.4% - Increased ARE- driven luciferase activity - Elevated mRNA and protein expression of Nrf2 downstream genes, like heme oxygenase-1 (Ho-1) - Activated the Nrf2-ARE pathway - Decreased CpG methylation in the Nrf2 promoter region - Downregulated the protein expression of DNMTs and HDACs ⇒ Delphinidin can activate the Nrf2-ARE pathway, which can be applied as a potential skin cancer chemopreventive agent	[143]
Pelargonidin (Purchased)	Mouse skin epidermal JB6 (JB6 P+) cells HepG2-C8 cells	- Decreased colony formation and suppressed cell viability of JB6 P+ cells - Reduced the protein levels of genes encoding DNMTs and HDACs - Decreased the DNA methylation in the Nrf2 promoter region of JB6 P+ cells - Increased Nrf2 downstream target genes expression ⇒ Pelargonidin exhibits its activity through activation of the Nrf2-ARE signaling pathway and its cytoprotective effect	[140]
Pelargonidin (Not reported)		- Specific interaction with cyclin-dependent kinase-1 (CDK1) - Five hydrogen bond interactions between DNMT1 and pelargonidin - Five hydrogen bond interactions between the ADD domain of DNMT3A and pelargonidin - Good interaction with DNMT1 - Inhibited the recognition of an unmethylated site ⇒ Pelargonidin can serve as a multitarget inhibitor for cancer treatment	[139]
**Biflavonoids**			
Amentoflavone (Selaginella tamariscina)	Glioma cell line: U87, LV229, U251, LN18 and U373	- Downregulated the miR-124-3p expression in glioma tissues - Decreased cell viability and triggered apoptosis in both glioma cell lines in a dose-dependent manner - Induced the apoptosis and inhibited glycolysis in the glioma cells by upregulating miR-124-3p - Upregulated the miR-124-3p by repressing DNMT1 through Sp1 ⇒ Amentoflavone could induce apoptosis and inhibit glycolysis in glioma cells via miR-124-3p	[144]
**Flavans**			
Kazinol Q (Purchased)	LNCaP prostate and MCF-7 and MDAMB-231 breast cancer	- Inhibited the recombinant DNMT1 - Reactivated the expression of a DNA methylation-silenced gene, E-cadherin, in MDA-MB-231 cells - Suppressed the proliferation of MCF-7 breast and LNCaP prostate cancer cells through apoptosis induction - Inhibited DNMT activity by competing with cytosine binding ⇒ Kazinol Q has a remarkable capacity to cause ROS, which, when combined with DNMT1 inhibition, could explain the role of kazinol Q on cancer cell proliferation and apoptosis.	[145]
**Flavanols**			
Catechin, Epicatechin, (-)-epigallocatechin-3-O-gallate (Purchased)	Human breast cancer cell lines (MCF-7 and MDA-MB-231)	- Inhibited SssI DNMT- and DNMT1-mediated DNA methylation in a concentration-dependent manner - Inhibited enzymatic DNA methylation in vitro by increasing the formation of S-adenosyl-_L_-homocysteine (a potent noncompetitive inhibitor of DNMTs) during the catechol-*O*-methyltransferase-mediated *O*-methylation ⇒ Inhibition of DNA methylation through two mechanisms: direct inhibition of the DNMTs, plus indirect inhibition of the enzymes through increased formation of SAH (a potent noncompetitive inhibitor of DNMTs)	[146]
(-)-Epigallocatechin-3-gallate (Not reported)	Female SKH-1 hairless mice	- Treatment with EGCG in hydrophilic cream resulted in high protection against photo-carcinogenesis when determined in terms of tumor incidence, tumor multiplicity, and tumor size in a SKH-1 hairless mouse model - Inhibited malignant transformation of UVB-induced papillomas to carcinomas - Inhibited the UVB-induced global DNA hypomethylation pattern ⇒ Hydrophilic cream as vehicle for topical application of EGCG is a promising candidate for future cancer therapies	[147]
(-)-Epigallocatechin-3-gallate (Purchased)	Urinary bladder transitional cell carcinoma (T24 cells), prostate adenocarcinoma (PC3 cells), and colorectal adenocarcinoma (HT29 cells)	- No measurable demethylating activity - Minor reductions in LINE repetitive element methylation levels (5–10%) in HT29 cells ⇒ 5-aza-2V-deoxycytidine (5-Aza-CdR) is far more effective in DNA methylation inhibition as well as in reactivating genes, compared with non-nucleoside inhibitors (EGCG)	[148]
(-)-Epigallocatechin-3-gallate (Not reported)	Esophageal squamous cell carcinoma cell lines, KYSE 510, 150 and 450	- Reversed the CpG hypermethylation status and restored mRNA expression of the *O*^6^-methylguanine-DNA methyltransferase (*MGMT*) gene in ESCC ⇒ DNA hypermethylation of MGMT (gene silencing in the development of ESCC), and this epigenetic event may be prevented or reversed by these polyphenols for the prevention of carcinogenesis.	[149]
(-)-Epigallocatechin-3-gallate (Purchased)	Human breast cancer cell lines (MCF-7 and MDA-MB-231)	- Inhibited SssI DNMT- and DNMT1-mediated DNA methylation in a concentration-dependent manner with a potent inhibition (IC_50_ values ranging from 0.21 to 0.47 μM) - The gallic acid moiety of EGCG had a crucial role in its high-affinity, direct inhibitory interaction with the catalytic site of the human DNMT1 ⇒ EGCG is a more potent inhibitor to SssI DNMT- and DNMT1-mediated DNA methylation	[146]
(-)-Epigallocatechin-3-gallate (Not reported)	Wild-type and APC 1309 knock-out mice	- Decreased methylation rate by 4% in the wild-type mice and 5% in the knock-out mice ⇒ EGCG may be a good candidate material for cancer prevention, anti-aging or cancer treatment without adverse effects	[150]
(-)-Epigallocatechin-3-gallate (Purchased)	TK6, Jurkat, and KG-1 leukemia cell models	- EGCG exhibited significant cytotoxicity in human cancer cell lines - EGCG was genotoxic, as evidenced by the induction of micronuclei ⇒ 5-aza-CR, 5-aza-CdR, zebularine, and RG108 caused concentration-dependent demethylation of genomic DNA, whereas EGCG failed to induce significant effects	[2]
(-)-Epigallocatechin-3-gallate (Purchased)	MCF-7 breast cancer and HL60 leukemia cells	- Reduced cellular proliferation and induced apoptosis in both MCF-7 and HL60 cells in vitro - Decreased *hTERT* (human telomerase reverse transcriptase) mRNA expression only in MCF-7 cells - Decreased *hTERT* promoter methylation and ablated histone H3 Lys9 acetylation in a time-dependent manner in MCF-7 cells ⇒ EGCG is effective in causing cell death in both MCF-7 and HL60 cancer cell lines through epigenetic modulation	[151]
(-)-Epigallocatechin-3-gallate (Purchased)	Human oral squamous cell carcinoma cell lines HSC3, HSC4, SCC9, SCC25 and human cervical cancer cell line HeLa	- Reversed the hypermethylation status of the *RECK* gene - Enhanced the expression level of *RECK* mRNA - Inhibited the matrix metalloproteinases (MMPs) levels (MMP-2 and MMP-9) in cells - Suppressed the cancer cell-invasive ability by decreasing the number of invasive foci as well as invasion depth in three-dimensional collagen invasion model ⇒ EGCG plays a key role in suppressing cell invasion through multiple mechanisms, possibly by demethylation effect on MMP inhibitors such as RECK	[152]
(-)-Epigallocatechin-3-gallate (Purchased)	SCC-13, A431 and HaCaT skin cancer cells	- Reduced the Bmi-1 and Ezh2 level in SCC-13 cells, which was associated with reduced cell survival - Reduced the histone H3 lysine 27 trimethylation - Reduced the expression of key proteins that enhance progression through the cell cycle - Increased the expression of proteins that inhibit cell cycle progression (p21 and p27) - Increased the Bax level and suppressed the Bcl-xL expression ⇒ EGCG reduces skin tumor cell survival by influencing PcG-mediated epigenetic regulatory mechanisms	[153]
(-)-Epigallocatechin-3-gallate (Purchased)	Human lung carcinoma cell lines H1703, H460 and A549 and colorectal cancer cell line HCT116	- Promoter demethylation of WIF-1 and restoration of WIF-1 expression in H460 and A549 cell lines - Decreased the cytosolic β- catenin protein level - Inhibited the Tcf/Lef reporter activity ⇒ Potential therapeutic use of EGCG for the reversal of WIF-1 promoter methylation	[154]
(-)-Epigallocatechin-3-gallate (Not reported)	-	- Inhibited the methyltransferase activity (DNMT1) (with an IC_50_ = 70 μM) - Inhibited the restriction enzyme *HhaI* (concentration range 10 μM–1 nM) ⇒ EGCG is one of the most prominent natural products associated with DNA methylation inhibition	[155]
(-)-Epigallocatechin-3-gallate (Purchased)	Breast cancer MCF-7 and MDA-MB-231 cells and normal control MCF10A cells	- Treatments with EGCG and a prodrug of EGCG (pEGCG) dose- and time-dependently inhibited the proliferation of human breast cancer cells - EGCG + pEGCG inhibited the transcription of hTERT through epigenetic mechanisms in estrogen receptor (ER)-positive MCF-7 and ER-negative MDA-MB-231 cells - Inhibited the DNMT and histone acetyltransferase (HAT) activities - Remodeled the chromatin structures of the hTERT promoter by decreasing the level of acetyl-H3, acetyl-H3K9, and acetyl-H4 to the hTERT promoter - Induced chromatin alterations that facilitate the binding of many hTERT repressors such as MAD1 and E2F-1 to the hTERT regulatory region ⇒ Pro-EGCG, a more stable form of EGCG, could be an effective chemopreventive candidate to breast cancer through epigenetic modulation of telomerase	[156]
(-)-Epigallocatechin-3-gallate (Purchased)	Human skin cancer A431 and squamous cell carcinoma (SCC) 13	- Decreased global DNA methylation levels in A431 cells in a dose-dependent manner - Decreased the levels of DNMT activity, mRNA, and protein levels of DNMT1, DNMT3a, and DNMT3b - Decreased the HDAC activity - Increased the levels of acetylated lysine 9 and 14 on histone H3 (H3-Lys 9 and 14) and acetylated lysine 5, 12 and 16 on histone H4 - Re-expressed the mRNA and proteins of silenced tumor suppressor genes, *p16^INK4a^* and Cip1/p21 ⇒ EGCG may contribute to the chemoprevention of skin cancer as epigenetic therapy	[157]
(-)-Epigallocatechin-3-gallate (Purchased)	HCT116, HEK293 and MRC5 cells	- Decreased the *hTERT* mRNA levels in HCT116 cells - Induced the upregulation of p53 and p21, and downregulation of DNMT1 - Demethylated several genomic sites including CCCTC binding factor (CTCF) and specificity protein 1 (SP1) binding sites, which are important regulatory elements of *hTERT* expression - Increased the reactive oxygen species (ROS) level which could induce the downregulation of DNMT1 expression with the upregulation of p53 and p21, resulting in *hTERT*-mediated cancer cell death ⇒ Preferential death of cancer cells by EGCG could be caused by the cancer-specific induction of reactive oxygen species (ROS) and epigenetic modulation of expression of apoptosis-related genes, such as *hTERT*	[158]
(-)-Epigallocatechin-3-gallate (Purchased)	Human T lymphocyte leukemic Jurkat cells	- Downregulated the ubiquitin-like containing PHD and ring finger 1 (UHRF1) and DNMT1 expression in Jurkat cells, with subsequent upregulation of *p73* and *p16^INK4A^* genes - EGCG-induced UHRF1 downregulation is ROS-dependent - Decreased binding of UHRF1 to the *p16^INK4A^* promoter - Over-expression of UHRF1 counteracted EGCG-induced cell cycle arrest and apoptosis - Over-expression of wild-type UHRF1 (In the presence of EGCG) decreased *p16^INK4A^*, p73a and p73b, whereas UHRF1 SRA (set and ring associated) mutants were inefficient except for R491A mutant which remained active ⇒ EGCG regulates UHRF1 expression, as well as that of DNMT1	[159]
(-)-Epigallocatechin-3-gallate (Purchased)	SKOV3-ip1 and SKOV3TR-ip2 cells Ovarian cancer cell lines	- EGCG potentiates the inhibiting effect of SFN on ovarian cancer cells - EGCG + SFN arrested cells in both G2/M and S phase - EGCG + SFN increased apoptosis in SKOV3TR-ip2 cells, while reducing the expression of *hTERT* ⇒ EGCG and SFN combination treatment can inhibit ovarian cancer cells by creating DNA damage through decreasing hTERT and Bcl-2 expression.	[160]
(-)-Epigallocatechin-3-gallate (Purchased)	Human colon cancer cell lines HT-29 and HCT 116	- Reduced HDAC and DNMT protein expression - Decreased the DNMT3B transcript in HCT 116 cells - Downregulated the DNMT transcripts in HT-29 cells - Decreased DNMT3A protein in the methylation-sensitive HCT 116 cell line in a time- and dose-dependent manner - Decreased HDAC2 and HDAC3 expression - Decreased association between UHRF1 and DNMT3 for both HCT 116 and HT-29 cell lines - Decreased association between UHRF1 and HDAC3 in only the HCT 116 cell line ⇒ EGCG, in combination with other DNMT and HDAC inhibitors, could be beneficial to treat colon cancer	[161]
(-)-Epigallocatechin-3-gallate (Purchased)	Human breast carcinoma cell lines MCF7 and MDA MB 231	- Decreased the transcript levels of all the DNMTs investigated (DNMT1, DNMT3a, and DNMT3b) - Decreased the protein levels of DNMT1, HDAC1, and MeCP2 ⇒ EGCG has the potential to reverse epigenetic changes	[162]
(-)-Epigallocatechin-3-gallate (Purchased)	Human colon adenocarcinoma cell line HT29 cells	- Inhibited the HDAC activity in intact HT29 cells - Decreased the HDAC1 protein level ⇒ EGCG possess promising HDAC-inhibitory properties, contributing to epigenetic alterations in colon tumor cells	[163]
(-)-Epigallocatechin-3-gallate (Purchased)	Human Burkitt’s Lymphoma CA46 cells	- EGCG inhibited CA46 cell proliferation - EGCG (6 μg/mL) + TSA (15 ng/mL) reduced CA46 cell proliferation from 24 to 96 h - EGCG + TSA decreased *p16^INK4A^* gene methylation, which coincided with increased *p16^INK4A^* mRNA and protein expression - EGCG + TSA reactivated *p16^INK4A^* gene expression in part through reducing promoter methylation, which may decrease CA46 cell proliferation ⇒ EGCG and TSA synergistically reactivate *p16^INK4a^* gene expression, in part through reducing promoter methylation, which may decrease CA46 cell proliferation	[164]
(-)-Epigallocatechin-3-gallate (Purchased)	Human established PDA cell lines BxPc-3 and MIA-PaCa2 and human hTERT-HPNE immortalized pancreatic duct cells CRL-1097	- Inhibited the colony formation - EGCG + quercetin inhibited viability, migration, expression of MMP-2 and -9, ALDH1 activity, colony, and spheroid formation and induced the apoptosis - EGCG + quercetin induced the expression of miR-let7-a in cancer cells ⇒ EGCG and quercetin complement each other in the inhibition of PDA progression by induction of miR-let7-a and inhibition of K-ras	[165]
(-)-Epigallocatechin-3-gallate (Not reported)	Breast cancer cell lines MCF-7 and MDA-MB-231	- EGCG mediated epigenetic induction of TIMP-3 (tissue inhibitor of matrix metalloproteinase-3) levels and play a key role in suppressing invasiveness and gelatinolytic activity of MMP-2 and MMP-9 in breast cancer cells - Induced TIMP-3 mRNA and protein levels - Reduced the enhancer of zeste homolog 2 (EZH2) and class I HDAC protein levels ⇒ EGCG treatment decreased EZH2 catalyzed trimethylation of Histone H3 at Lysine 27 (H3K27me3) with a corresponding increase in the deposition of transcriptionally-active acetylated Histone H3 at Lysine 9/18 (H3K9/18 Ac), specifically at the TIMP-3 promoter	[166]
(-)-Epigallocatechin-3-gallate (Purchased)	RKO (CRL-2577), HCT-116 (CCL-247) and HT-29 (HTB-38) colorectal cancer cells	- Combinatorial effects of EGCG and NaB: - Induced apoptosis and cell cycle arrest in RKO, HCT-116, and HT-29 colorectal cancer cells - Suppressed RKO CRC cell proliferation - Arrested RKO, HCT-116 CRC cells predominantly in the G2/M phase and HT-29 CRC cells in the G1 phase - Increased p21, NF-κB-p65, HDAC1; and decreased DNMT1 and survivin in RKO CRC cells - Inhibited the HDAC1, DNMT1 and survivin in all the three CRC cells tested - Inhibited the DNMT3A and DNMT3B in total protein assessed in RKO CRC cells - Induced the p21 through a p53-dependent mechanism in RKO CRC cells - Affected the global DNA methylation ⇒ At low and physiologically achievable concentrations, combinatorial EGCG and NaB are effective in promoting apoptosis, inducing cell cycle arrest and DNA-damage in CRC cells	[167]
(-)-Epigallocatechin-3-gallate (Purchased)	CAL-27 human oral squamous cell carcinoma cell line	- Inhibited the cell growth in a concentration-dependent manner - Of the 84 genes altered in response to EGCG treatment, 57 were hypermethylated and 24 were hypomethylated - The main pathways involved were metabolic, mitogen-activated protein kinase (MAPK), wnt, and cell cycle pathways ⇒ EGCG can affect the methylation status and gene expression in the CAL-27 cell line	[168]
(-)-Epigallocatechin-3-gallate (Purchased)	Human cervical carcinoma cell line HeLa	- Inhibited the activity of DNMTs - Reduced the mRNA transcription level of DNMT3B - Decreased the HDAC activity time-dependently - No significant changes in the expression of HDAC1 - Zn ion was taken to be the substrate binding site of HDAC1 - Co-factor binding residue Glu-605 was defined as the substrate-binding cavity for DNMT3B - The docking results produced 34 clusters of ligand EGCG around the modeled catalytic domain of DNMT3B - The docking results produced 31 clusters of ligand EGCG around the complete protein HDAC1 - Restored the expression of RARβ, CDH1, and DAPK1 genes via the reversal of 5’ cytosine guanine dinucleotide (CpG) island methylation and inhibition of epigenetic modulators (DNMTs and HDACs) ⇒ EGCG may have a significant impact on the development of novel epigenetic-based therapy	[169]
(-)-Epigallocatechin-3-gallate (Purchased)	Human breast cancer cell lines (MCF-7, MDA-MB 231) and non-tumorigenic MCF-10A breast epithelial cells	- Inhibited the growth of breast cancer cells by co-treatment with 5-aza 20′ dC and EGCG - Increased the changes in DNA methylation and histone modifications ⇒ Potential growth inhibition of breast cancer cells by 5-aza 2′ dC and EGCG combination treatment, in part mediated by epigenetic mechanism	[170]
(-)-Epigallocatechin-3-gallate (Purchased)	A549 and DDP-resistant A549/DDP human lung adenocarcinoma cell	- In vitro EGCG + cisplatin (DDP) treatment caused: proliferation inhibition, cell cycle arrest in G1 phase, increase in apoptosis in A549/DDP cells, inhibition of DNMT and HDAC activities, reversal of hypermethylated status, and downregulated expression of GAS1, TIMP4, ICAM1, and WISP2 gene in A549/DDP cells - In vivo EGCG + DDP pre-treatment caused: - Inhibition of tumors, decrease in methylation levels of GAS1, TIMP4, ICAM, and WISP2 and increase in their expression levels ⇒ EGCG pretreatment resensitized cells to DDP, along with the demethylation and restoration of expression of candidate genes	[171]
(-)-Epigallocatechin-3-gallate (Not reported)	Human APL NB4 and HL-60 cells	- Inhibited the APL cell proliferation and apoptosis - Elevated the expression of genes associated with cell cycle arrest and differentiation (*p27*, *PCAF*, *C/EBPα*, and *C/EBPε*) - Downregulated the epigenetic modifiers DNMT1, HDAC1, HDAC2, and G9a - Downregulated the polycomb repressive complex 2 (PRC2) core components in gene and protein level - Enhanced the hyperacetylated H4 and acetylated H3K14 histones binding to the promoter regions of *p27*, *PCAF*, *C/EBPα*, and *C/EBPε* - Reduced the binding effect to PRC2 core component genes *EZH2*, *SUZ12*, and *EED* ⇒ EGCG, as cell proliferation inhibitor and epigenetic modifier, might be useful for APL treatment	[172]
(-)-Epigallocatechin-3-gallate (Purchased)	ERα (+) MCF-7 and ERα (-) MDA-MB-157, MDA-MB-231, and HCC1806 breast cancer	- Affected the expression of oncogenic miR-221/222 and tumor suppressors (p27 and PTEN) and to estrogen receptor alpha (ERα) - Decreased the activity of DNMTs - Enriched the AcH3 within the promoter of p27 and *PTEN* ⇒ EGCG has a role in limiting the growth and proliferation of breast cancer cells	[173]
(-)-Epigallocatechin-3-gallate (Purchased)	MCF7 and MDA-MB-231 breast cancer cells	- EGCG + clofarabine synergistically: - Inhibited the growth and induced apoptosis of breast cancer cells - Induced the retinoic acid receptor beta (RARB) hypomethylation - Increased the *RARB*, *PTEN*, and *CDKN1A* transcript levels ⇒ ClF-based combinations with EGCG promote cancer cell death and reactivate DNA methylation-silenced tumor suppressor genes in breast cancer cells with different invasive potential	[174]
(-)-Epigallocatechin-3-gallate (Purchased)	Human cervical cancer cell line Hela	- EGCG in combination with eugenol + amrogentin: - Inhibited cellular proliferation and colony formation - Induced apoptosis - Downregulated the cyclinD1 and upregulated the cell cycle inhibitors LIMD1, RBSP3, and p16 at G1/S phase of cell cycle - Induced promoter hypomethylation of LimD1 and P16 genes as a result of reduced expression of DNMT1 ⇒ EGCG, in combination with eugenol–amarogentin, demonstrates a better chemotherapeutic effect on the Hela cell line, which might be due to the epigenetic modification, and in particular DNA hypomethylation through downregulation of DNMT1	[175]
(-)-Epigallocatechin-3-gallate (Not reported)	Myeloid leukemia NB4 and K562 cells	- Inhibited the proliferation and survival of both cell lines - Induced the apoptosis of NB4 cells only - Induced the cell cycle arrest in G0/G1 phase - Increased the levels of ATM, HMGA2, phosphorylated ATM, and SA-β-galactosidase staining (indicated that EGCG caused cellular senescence) - Induced beneficial epigenetic modulation of epigenetic players DNMT1, HP1α, H3K9me3, EZH2, and SUZ12 in NB4 cells ⇒ EGCG is a promising epigenetic modulator for acute promyelocytic leukemia therapy and as a potential cellular senescence inducer in both acute and chronic myeloid leukemia treatment	[176]
(-)-Epigallocatechin-3-gallate (Not reported)	Human prostate cancer DUPRO and LNCaP cells	- EGCG (20 μM) mediated the epigenetic reactivation of TIMP-3 (metalloproteinase inhibitor 3) - Induced TIMP-3 mRNA and protein expression - Decreased the expression of both enhancers of zeste homolog 2 (*EZH2*) and its catalytic product trimethylation of histone H3 - Increased the histone H3K9/18 acetylation - Reduced class I HDAC activity and *EZH2* and H3K27me3 levels - Reduced MMP-2/MMP-9 gelatinolytic activity and abrogated invasion and migration capabilities ⇒ TIMP-3 induction, as a key epigenetic event modulated by green tea (EGCG) in restoring the MMP:TIMP balance suppresses prostate cancer progression	[177]
(-)-Epigallocatechin-3-gallate (Purchased)	Human breast cancer cell lines MCF-7 (cat. no. HTB-22) and MDA-MB 231	- Enhanced the *SCUBE2* gene, elevated E-cadherin, and decreased vimentin expression, leading to significant suppression of cell migration and invasion - Reversed the *SCUBE2* methylation - Decreased the *SCUBE2* methylation status by reducing DNMT expression and activity ⇒ *SCUBE2* methylation can be reversed by EGCG treatment	[178]
(-)-Epigallocatechin-3-gallate (Not reported)	Human microvascular endothelial cells (HMEC-1) and human umbilical vein endothelial cells (HUVECs)	- Increased histone acetylation (H3K9/14ac, H3ac), and methylation of both active (H3K4me3) and repressive (H3K9me3) chromatin marks - Inhibited the HDAC activity in cellular and cell-free models - Reduced the expression of heterochromatin binding proteins (HP1α, HP1γ) - Affected the expression and activity of epigenome modulators (HDAC5 and 7, p300, CREBP, LSD1 or KMT2A) ⇒ EGCG promotes chromatin relaxation in human endothelial cells and presents a broad epigenetic potential affecting expression and activity of epigenome modulators including HDAC5 and 7, p300, CREBP, LSD1 or KMT2A	[179]
(-)-Epigallocatechin-3-gallate (Purchased)	ERα (+) MCF-7 and ERα (–) MDA-MB-157, MDA-MB- 231, and HCC1806 breast cancer cells	- Decreased the expression of cIAP2 (cellular inhibitor of apoptosis 2) while increasing the expression of pro-apoptotic caspase 7 - Induced changes in histone modifications, resulted in an increase in apoptosis - EGCG + SAHA (Suberoylanilide hydroxamic acid) limited TNBC cell migration across a fibronectin matrix ⇒ SAHA and EGCG reduce the metastatic potential of TNBC by inducing the apoptotic pathway	[180]
**Flavanones**			
Hesperetin (Not reported)	NCI-60 cell line	- Acted as a promising inhibitor that targets ABL1, DNMT3B, and MLH1 due to the similarity of binding properties with its native ligand - Hesperetin + the erbB receptor inhibitors (monoclonal antibody and tyrosine kinase inhibitor) targeted the same mRNA expression ⇒ Combinatorial therapy with hesperetin targeting ABL1, DNMT3B, and MLH1 may be effective in circumventing chemoresistance in breast cancer	[181]
Hesperidin (Purchased)	Human prostate carcinoma cells DU145	- Inhibited the cell proliferation - Increased the level of DSBs - Increased the frequency of p53 binding protein (53BP1) foci - Decreased the level of 5-methyl-20-deoxycytidine (5mdC) ⇒ Hesperidin may induce epigenetic changes	[182]
Hesperidin (Not reported)	HL60 human leukaemia cancer Diethylnitrosamine-induced hepatocarcinogen	- Induced cytotoxic effect in a dose-dependent manner - Exerted a significant hypomethylating effect on the LINE-1 sequence and on the ALU- M2 repetitive sequences in HL60 tumor cells ⇒ Hesperidin could be proposed as a candidate molecule in chemoprevention for epigenetic therapy purposes	[183]
Naringenin (Not reported)	Mesangial cell (MMCs)	- Inhibited the Mesangial cell MMCs proliferation - Arrested cells in phase G2 - Down-expressed let-7a in both diabetic nephropathy rats and MMCs - Affected the expressions of collagen IV and fibronectin through upregulating let-7a in MMCs - Inhibited the TGF-*β*1/smads signaling activation by upregulating let-7a ⇒ Naringenin ameliorates kidney injuries by regulating let-7a/TGFBR1 signaling	[184]
Naringenin (Purchased)	Human colon adenocarcinoma (CaCo-2)	- Induced cell cytotoxicity - Decreased the expression levels of *miR-17-3p* and *miR-25-5p* for both miRNAs - Exerted an antioxidant activity through epigenetic regulation operated by miRNAs ⇒ Naringenin could exert its antioxidant activity through epigenetic regulation operated by miRNAs, while anti-inflammatory activity is regulated by other miRNAs and/or mechanisms	[185]
**Flavanonols**			
Taxifolin (Purchased)	Human hepatoma HepG2 cells	- Induced the antioxidant response element (ARE) on luciferase activity in HepG2-C8 cells - Induced the expression of Nrf2 and its downstream target genes in JB6 P+ cells by CpG demethylation - Reduced the methylation level of the first 15 CpGs sites in the Nrf2 promoter - Inhibited the expression levels of DNMT and HDAC proteins ⇒ Taxifolin may exhibit a skin cancer preventive effect by activating Nrf2 via an epigenetic pathway	[186]
**Flavones**			
3,6-dihydroxyflavone (3,6-DHF) (Purchased)	Human breast epithelial MCF-10A cells	- Inhibited the carcinogens-induced breast carcinogenic transformation - Downregulated miR-34a and upregulated miR-21 in breast carcinogenesis - Inhibited the hypermethylation of the miR-34a promoter - Decreased the DNMT activity in a dose-dependent manner - Lowered the H3K9-14ac on the miR-21 promoter - Repressed the PI3K/Akt/mTOR signaling pathway in breast carcinogenesis in vitro and in vivo ⇒ 3,6-DHF upregulates miR-34a via inhibiting DNMT1 and hypermethylation, whereas it downregulates miR-21 by modulating histone modification, and consequently suppresses the PI3K/Akt/mTOR signaling pathway in breast carcinogenesis	[187]
3,6-dihydroxyflavone (3,6-DHF) (Purchased)	MCF-10A and MDA-MB-231 cells Mammary gland and tumor samples (in vivo)	- Promoted the expression of TET1 during carcinogen-induced breast carcinogenesis in MCF10A cells and in rats - Increased TET1 and 5hmC levels in MDA-MB-231 cells - Increased the TET1 expression by inhibiting DNMT1 and DNA hypermethylation, and consequently upregulated the miR-34a in breast carcinogenesis ⇒ 3,6-DHF effectively increases TET1 expression by inhibiting DNMT1 and DNA hypermethylation, and consequently upregulates miR-34a in breast carcinogenesis	[188]
Apigenin (Purchased)	Human prostate cancer LNCaP and DU145 cells and transformed human prostate epithelial RWPE-1 cells	- Intercalation as the dominant binding mode, with specific binding to a GC-rich sequence in the DNA duplex - Apigenin was tethered at both ends inside the catalytic pocket of DNMT and EZH2 by means of hydrogen bonding (virtual screening) - Decreased the DNMT enzyme activity - Reversed the hypermethylation of cytosine bases in the DNA - Prevented the cytosine methylation in the GC- rich promoter sequence incubated with the M.SssI enzyme - Decreased the HMT activity - Decreased the EZH2 protein expression and trimethylation of H3K27 ⇒ Apigenin anticancer effect is mediated by altering epigenetic processes involved in the development of cancer	[189]
Apigenin (Purchased)	Human prostate cancer cell lines 22Rv1 and PC-3	- Inhibited the class I HDACs in prostate cancer cells - Inhibited the HDAC enzyme activity, specifically HDAC1 and HDAC3 - Induced global histone H3 and H4 acetylation, as well as localized hyperacetylation of histone H3 on the p21/waf1 promoter - Increased p21/waf1 and bax protein and mRNA expression - Reduced tumor growth, HDAC activity, HDAC1, and HDAC3 protein expression (in vivo) - Increased the expression of p21/waf1 (in vivo) - Decreased the expression of bcl2 with concomitant increase in bax, shifting the bax/bcl2 ratio in favor of apoptosis ⇒ Apigenin inhibits class I HDACs, particularly HDAC1 and HDAC3, and its exposure results in the reversal of aberrant epigenetic events that promote malignancy	[190]
Apigenin (Purchased)	Skin epidermal JB6 P+ cells	- Reversed the hypermethylated status of the 15 CpG sites in the Nrf2 promoter in a dose-dependent manner - Enhanced the nuclear translocation of Nrf2 and increased the mRNA and protein expression of Nrf2 and the Nrf2 downstream target gene (NQO1) - Reduced the expression of the DNMT1, DNMT3a, and DNMT3b epigenetic proteins as well as the expression of some HDACs (1–8) ⇒ Apigenin can restore the silenced status of Nrf2 in skin epidermal JB6 P+ cells by CpG demethylation coupled with attenuated DNMT and HDAC activity	[191]
Apigenin (Purchased)	MDA-MB-231 human breast cancer cells	- Inhibited cell proliferation - Induced cell cycle arrest at the G_2_/M phase - Suppressed the expression of cyclin A, cyclin B, and CDK1, which control the G_2_-to-M phase transition in the cell cycle - Upregulated the p21^WAF1/CIP1^ and increased the interaction of p21^WAF1/CIP1^ with proliferating cell nuclear antigen (PCNA), which inhibits cell cycle progression - Inhibited the HDAC activity - Induced the histone H3 acetylation - Increased the acetylation of histone H3 in the p21^WAF1/CIP1^ promoter region, resulting in the increase in p21^WAF1/CIP1^ transcription - Delayed the tumor growth (in vivo) ⇒ Apigenin can be used in breast cancer prevention and treatment through epigenetic regulation	[192]
Baicalein (Purchased)	MCF7 breast cancer cells	- Inhibited the DNMT activity - No effect on the methylation pattern or the expression of *RASSF1A*, *GSTP1* or *HIN-1* in MCF7 cells - No effect on the global methylation of histone H3 - No effect on DNMT1 transcription or on DNMT1 protein level ⇒ Baicalein is unlikely to be effective epigenetic modulators	[137]
Casticin (Purchased)	MGC803 gastric cancer cells	- Increased *RECK* (reversion-inducing-cysteine-rich protein with Kazal motifs) protein expression and mRNA levels - Decreased *RECK* promoter methylation levels (31%), global DNA methylation levels (39%), and nuclear methylation activity (71.6%) - Downregulated the mRNA levels and protein expression of DNMT1 - Inhibited MGC803 cell proliferation - Reduced the DNA-binding activity of Sp1 ⇒ Casticin inhibits the proliferation of gastric cancer MGC803 cells by upregulating RECK gene expression and reducing intracellular methylation levels	[193]
Casticin (Not reported)	MHCC97H, SK-Hep-1, and L02 hepatic cancer cells	- Reduced the viabilities of HCC cells but not L02 cells - Inhibited the stemness characteristics in HCC cells - Repressed DNMT1 activity and expression - Increased *miR-148a-3p* ⇒ Casticin could inhibit stemness characteristics in HCC cells by interruption of the reciprocal negative regulation between DNMT1 and *miR-148a-3p*	[194]
Chrysin (Purchased)	Human prostate cancer LNCaP and DU145 cells and transformed human prostate epithelial RWPE-1 cells	- Intercalation as the dominant binding mode, with specific binding to a GC-rich sequence in the DNA duplex - Chrysin is tethered at both ends inside the catalytic pocket of DNMT and EZH2 by means of hydrogen bonding (virtual screening) - Decreased the DNMT enzyme activity - Reversed the hypermethylation of cytosine bases in the DNA - Prevented the cytosine methylation in the GC- rich promoter sequence incubated with the M.SssI enzyme - Decreased the HMT activity - Decreased the EZH2 protein expression and trimethylation of H3K27 ⇒ The chrysin anticancer effect is mediated by altering epigenetic processes involved in the development of cancer	[189]
Diosmin (Purchased)	Human breast cancer cells MCF-7, MDA-MB-231 and SK-BR-3	- Induced the G2/M cell cycle arrest - Induced the elevation in p53, p21, and p27 levels and stress-induced premature senescence in breast cancer cells - Promoted the apoptosis in breast cancer cells - Stimulated oxidative and nitrosative stress, DNA damage and changes in global DNA methylation patterns - Affected the methylation parameters in breast cancer cell lines (global DNA methylation and the levels of methyltransferases) - Induced autophagy in breast cancer cells ⇒Diosmin-induced premature senescence and cytotoxic autophagy leading to apoptotic cell death may be considered as an attractive anticancer strategy	[195]
Diosmin (Purchased)	Human prostate carcinoma cells (DU145)	- Inhibited the cell proliferation (10–30%) at the highest concentration (250 μM) - Induced apoptotic and necrotic cell death (250 μM) - Induced the DNA double strand breaks (DSBs) and micronuclei production - Increased the frequency of p53 binding protein (53BP1) foci ⇒ Diosmin may induce DNA and chromosomal damage in DU145 prostate cancer cells, which, in turn, may provoke apoptotic cell death and may have implications for diosmin-based anticancer therapy	[182]
Isovitexin (Purchased)	Human OS U2OS and MG63 cell lines	- Repressed the survival, induced apoptosis and decreased the level of *CD133*, *CD44*, *ABCG2*, and *ALDH1* mRNA in the spheres derived from U2OS and MG63 cells - Reduced the sphere formation rate of U2OS-SC and MG63-SC - Inhibited the tumor growth and reduced tumor size of U2OS-SC xenografts in nude mice - Reduced the DNMT1 activity and expression, increased miR-34a, and decreased Bcl-2 levels ⇒ Isovitexin-mediated epigenetic regulation involves the DNMT1/miR-34a/Bcl-2 axis and causes the suppression of stemness and induction of apoptosis in the spheres derived from OS cells	[196]
Luteolin (Purchased)	Human lung cancer cells LNM35	- Increased the sub-G1 (apoptotic) fraction of cells through caspase-3 and -7 dependent pathways - Inhibited the invasive potential of LNM35, MCF-7/6, and MDA-MB231- 1833 cancer cells - Inhibited the HDAC activity - Potentiated the cytotoxicity of cisplatin in LNM35 cells - Decreased the growth of LNM35 tumor xenografts in athymic mice ⇒ Luteolin, in combination with standard anticancer drugs such as cisplatin, may be a promising HDAC inhibitor for the treatment of lung cancer	[197]
Luteolin (Not reported)	Human breast cancer BT474, MCF-7 and MDA-MB-231 cells	- Upregulated the expression of opioid binding protein/cell adhesion molecule (OPCML) in breast cancer cells - Activated the OPCML by reducing intracellular methylation levels - Downregulated the intracellular methylation levels by decreasing Sp1 and NF-κB activities - Affected the expression of DNMT1 and *OPCML* by downregulating Sp1 activity - Inhibited the proliferation and induced the apoptosis of BT474 and MCF-7 cells ⇒ Luteolin inhibits the growth of breast cancer cells by decreasing methylation and upregulating the expression of the *OPCML* gene	[198]
Luteolin (Purchased)	Human HT-29 colon cancer and SNU-407 cells	- Increased the expression of apoptosis-related proteins and antioxidant enzymes - Inhibited the expression of DNMTs - Increased the expression and activity of ten-eleven translocation (TET) DNA demethylases - Decreased the methylation of the Nrf2 promoter region, which corresponded to the increase mRNA expression of Nrf2 - Increased the TET1 binding to the Nrf2 promoter ⇒ The mechanism that underlies the anticancer effects of luteolin on colon cancer involves the upregulation of Nrf2 and its interaction with the tumor suppressor	[199]
Luteolin (Purchased)	Human colon cancer cell line BE	- Induced the cytotoxicity and cell cycle perturbation in a dose-dependent manner - Induced the apoptosis of BE colorectal cancer cells - Downregulated the DNMT1 expression and the epigenetic integrator ubiquitin-like containing UHRF1 - Induced an upregulation of a tumor suppressor gene: *p16^INK4A^* - Inhibited the calpain activity ⇒ Targeting calpain, UHRF1, and DNMT1 using luteolin could be an interesting way to prevent and/or treat colorectal cancers	[200]
Luteolin (Not reported)	KB cells and Hnscc cancer xenograft mouse	- Inhibited the p300 acetyltransferase with competitive binding to the acetyl CoA binding site - Reduced tumor growth corresponding to a decrease in histone acetylation - Induced the cell cycle arrest - Decreased cell migration - Altered the gene expression and miRNA profile, including upregulation of p53 induced miR-195/215, let7C - Downregulated the oncomiRNAs, such as miR-135a ⇒ Luteolin effects were observed at multiple levels: gene expression, miRNA expression and miRNA processing	[201]
Luteolin (Purchased)	Human colorectal cancer HCT116 cells	- Suppressed cell proliferation and cellular transformation of HCT116 and HT29 cells in a dose-dependent manner - Activated the Nrf2 and its target genes on both mRNA and protein levels - Decreased methylation of the Nrf2 promoter region - Increased the mRNA expression of Nrf2 - Decreased protein levels and enzyme activities of epigenetic modifying enzymes (DNMTs and HDACs) in HCT116 cells ⇒ Luteolin may exert its antitumor activity in part via epigenetic modifications of the Nrf2 gene, with subsequent induction of its downstream antioxidative stress pathway	[202]
Luteolin (Purchased)	Human prostate cancer LNCaP and DU145 cells and transformed human prostate epithelial RWPE-1 cells	- Intercalation as the dominant binding mode, with specific binding to a GC-rich sequence in the DNA duplex - Luteolin is tethered at both ends inside the catalytic pocket of DNMT and EZH2 by means of hydrogen bonding (virtual screening) - Decreased the DNMT enzyme activity - Reversed the hypermethylation of cytosine bases in the DNA - Prevented the cytosine methylation in the GC- rich promoter sequence incubated with the M.SssI enzyme - Decreased the HMT activity - Decreased the EZH2 protein expression and trimethylation of H3K27 ⇒ Luteolin anticancer effect is mediated by altering epigenetic processes involved in the development of cancer	[189]
Naringin (Purchased)	Human prostate carcinoma cells (DU145)	- Inhibited the cell proliferation (10–30%) at the highest concentration (250 μM) - Increased the level of DSBs (1.57-fold) at 150 μM - Increased the frequency of p53 binding protein (53BP1) foci - Decreased the level of 5-methyl-20-deoxycytidine (5mdC) ⇒ Naringin may induced epigenetic changes	[182]
Pectolinarigenin (Purchased)	143B, HOS and MG63 osteosarcoma	- Inhibited the STAT3 activity - Disturbed the STAT3/DNMT1/HDAC1 complex formation in the promoter region of SHP-1, which reversely mediates STAT3 signaling, leading to the upregulation of SHP-1 expression in osteosarcoma - Suppressed osteosarcoma cell proliferation - Induced apoptosis - Reduced the level of STAT3 downstream proteins cyclin D1, Survivin, B-cell lymphoma 2 (Bcl-2), B-cell lymphoma extra-large (Bcl-xl), and myeloid cell leukemia 1 (Mcl-1) - Inhibited adhesion, migration, invasion, and reverse EMT phenotype in osteosarcoma cells ⇒ Pectolinarigenin may be a candidate for osteosarcoma intervention linked to its STAT3 signaling inhibitory activity	[203]
**Flavonolignans**			
Silibinin (Purchased)	Human prostate cancer cell lines DU145 and PC3	- Suppressed PRC2 (Polycomb Repressive Complex 2) expression - Reduced the EZH2 (Enhancer of Zeste Homolog 2) expression while increasing trimethylation levels of Lys27 on H3 (H3K27me3) - Induced a modest concentration-dependent increase in DNMT activity - Induced a concentration-dependent decrease in HDAC1-2 expression levels ⇒ Silibinin induces epigenetic alterations in human prostate cancer cells	[204]
Silibinin (Purchased)	Human bladder cell lines RT4 and T24	- Inhibited the human UCC growth - Induced DNA damage - Induced low apoptosis rates in human UCC - Induced the FRAP/mTOR, FGFR3, DNMT1, AKT2, and miR100 modulation only in the wild-type TP53 human UCC ⇒ Silibinin is an antiproliferative compound, whose mechanism of action was related to the TP53 status	[205]
Silibinin (Purchased)	SW480 and SW620 cells colon adenocarcinoma	- No effect on the activity of HDACs - Inhibited the DNMT activity in both SW480 and SW620 cells - Induced cell death ⇒ Silibinin inhibited DNMT but not HDAC activity in colorectal SW480 and metastatic SW620 cells, and exerted synergistic effects with HDAC inhibitors on cancer cell death	[206]
Silibinin (Not reported)	Human NSCLC H1299 cells	- Decreased the HDAC activity in a dose-dependent manner - Decreased the protein levels of HDAC1, HDAC2, HDAC3, DNMT1, and DNMT3A - Downregulated the levels of DNMT3A, HDAC1, HDAC6, SET domain proteins (SETD1A, D4, D6), and lysine specific demethylases (KDM 5B, 5C, 4A) ⇒ Silibinin inhibits specific histone deacetylases, histone demethylases and DNA methyltransferases, which might cooperatively contribute to the anti-cancer efficacy of this non-toxic phytochemical	[207]
**Flavonols**			
Fisetin (Purchased)	Human breast cancer cell lines (MCF-7 and MDA-MB-231)	- Inhibited SssI DNMT- and DNMT1-mediated DNA methylation in a concentration-dependent manner ⇒ Fisetin inhibits DNA methylation through two mechanisms: direct inhibition of the DNMTs, and indirect inhibition of the enzymes through increased formation of SAH (a potent noncompetitive inhibitor of DNMTs)	[146]
Galangin (Not reported)	MCF7 breast cancer cells	- Inhibited the DNMT activity - No effect on the methylation pattern or the expression of *RASSF1A*, *GSTP1* or *HIN-1* in MCF7 cells ⇒ Galangin as a non-nucleoside agent is unlikely to be effective epigenetic modulator; nevertheless, a long-term exposure to it can lead to a potential effect	[137]
Galangin (Purchased)	Human neuroblastoma SH-SY5Y cells	- Reduced the *BACE1* at mRNA and protein levels - Decreased the acetylated H3 in the BACE1 promoter regions through the upregulation of endogenous HDAC1-mediated deacetylation, which is independent of DNA methylation status ⇒ Galangin proposes a neuroprotective mechanism of polyphenols against alzheimer’s disease through an epigenic modifications of AD-related genes	[208]
Kaempferol (Not reported)	Human-derived cell lines HepG2, Hep3B	- Inhibited the HDACs activity - Bound to human HDAC enzymes - Induced the hyperacetylation of histone complex H3 - Reduced cell viability and proliferation rate ⇒ Kaempferol could serve as a new lead structure to design and investigate new drugs with an enhanced inhibition activity	[209]
Kaempferol (Purchased)	Human GC cell lines (AGS, SNU-216, NCI-N87, SNU-638, and MKN-74)	- Induced an increased ubiquitination of DNMT3B - Promoted autophagy and cell death - Increased LC3-I to LC3-II conversion and the downregulation of p62 in GC - Induced the autophagic cell death via the activation of the IRE1-JNK-CHOP signaling, indicating ER stress response - Inhibited the G9a (HDAC/G9a axis) and activated autophagic cell death ⇒ Kaempferol activates the IRE1-JNK-CHOP signaling from cytosol to nucleus, and G9a inhibition activates autophagic cell death in GC cells	[210]
Kaempferol (Purchased)	T24 and 5637 bladder cancer cell lines	- Modulated the DNA methylation in bladder cancer - Inhibited the protein levels of DNMT3B without altering the expression of DNMT1 or DNMT3A - Induced a premature degradation of DNMT3B by inhibiting protein synthesis with cycloheximide (CHX) ⇒ Kaempferol may promote the degradation of DNMT3B in bladder cancer	[211]
Morin (Purchased)	Human ovarian cancer cell lines, A2780 and SKOV-3	- Inhibited the adhesive and migratory potential of both cell lines and the accumulation of G0/G1 phase A2780 cells - Induced the downregulation of genes considered as upregulated during EMT (epithelial to mesenchymal transition), and upregulation of some genes considered as downregulated during EMT in A2780 and SKOV-3 cells - Decreased the expression level of genes associated with adhesion - Increased genes downregulated during EMT ⇒ Morin induced a phenotypic changes due to some molecular changes in cells, eg., decrease in the expression level of genes associated with adhesion, and an increase in genes downregulated during EMT	[212]
Myricetin (Purchased)	Human breast cancer cell lines (MCF-7 and MDA-MB-231)	- Inhibited SssI DNMT- and DNMT1-mediated DNA methylation in a concentration-dependent manner ⇒ Myricetin inhibits DNA methylation through two mechanisms: direct inhibition of the DNMTs plus indirect inhibition of the enzymes through increased formation of SAH (a potent noncompetitive inhibitor of DNMTs)	[146]
Myricetin (Purchased)	MCF7 breast cancer cells	- Inhibited the DNMT activity - No effect on the methylation pattern or the expression of *RASSF1A*, *GSTP1* or *HIN-1* in MCF7 cells ⇒ Myricetin was not found to be effective epigenetic modulator	[137]
Quercetin (Purchased)	Human breast cancer cell lines (MCF-7 and MDA-MB-231)	- Inhibit SssI DNMT- and DNMT1-mediated DNA methylation in a concentration-dependent manner ⇒ Quercetin inhibits DNA methylation through two mechanisms: direct inhibition of the DNMTs and indirect inhibition of the enzymes through increased formation of SAH (a potent noncompetitive inhibitor of DNMTs)	[146]
Quercetin (Not reported)	Human colon cancer cell line RKO cells	- Inhibited the growth of RKO cells - Induced the demethylation of the *p16^INK4a^* gene promoter in RKO cells in a dose-dependent manner - Restored the transcription of *p16^INK4a^* mRNA - Increased the expression of p16*^INK4a^* protein ⇒ Quercetin has antitumor properties, probably via demethylation of the *p16^INK4a^* gene promoter	[213]
Quercetin (Not reported)	Human breast cancer cell lines MCF-7 and MDA-MB-231	- Inhibited the growth of MCF-7 and MDA-MB-231 cells in a dose-and time-dependent manner - Increased the levels of the miR-146a expression in MCF-7 and MDA-MB-231 in a dose-dependent manner - Stimulated the expression of bax and caspase-3 - Inhibited the expression of EGFR (epidermal growth factor receptor) via upregulation of the miR-146a expression - Inhibited the tumor growth in nude mouse ⇒ Quercetin inhibits cell proliferation in human breast cancer cells by upregulating miR-146a expression, and downregulating the expression of EGFR	[214]
Quercetin (Purchased)	Human osteosarcoma 143B cell line	- Inhibited the viability of 143B osteosarcoma cells - Enhanced the cisplatin sensitivity of 143B - Enhanced cisplatin sensitivity of 143B osteosarcoma cells by modulating miR-217-KRAS axis ⇒ Quercetin enhances the cisplatin sensitivity by modulating the miR- 217-KRAS axis	[215]
Quercetin (Purchased)	Human Pancreatic ductal adenocarcinoma PDA cell lines BxPc-3 and MIA-PaCa2 and human hTERT-HPNE immortalized pancreatic duct cells CRL-1097	- Inhibited viability, migration, expression of MMP-2 and -9, ALDH1 activity, colony, and spheroid formation and induced the apoptosis - Induced the expression of miR-let7-a ⇒ Quercetin inhibits PDA progression by induction of miR-let7-a and inhibition of K-ras	[165]
Quercetin (Purchased)	Human cervical cancer cells HeLa cells	- Reduced the DNMT and HDAC activity - Reduced the HMT-H3K9 activity - Modulated the expression of various chromatin modifiers and decreased the activity of DNMTs, HDACs, and HMTs in a dose-dependent manner - Downregulated the global DNA methylation levels in a dose- and time-dependent manner - Interacted with residues in the catalytic cavity of several DNMTs and HDACs (as a competitive inhibitor) ⇒ Quercetin could be a candidate for epigenetic-based anticancer therapy	[216]
Quercetin (Purchased)	MDA-MB-231, MDA-MB-468, T47D, BT-20, and MCF-7 breast cancer cells	- Enhanced the BRCA1 (breast cancer type 1 susceptibility protein) expression levels in TNBC (triple-negative breast cancer) - Inhibited the cell survival of TNBC cell lines - Enhanced the BRCA1 expression - Induced the histone acetylation of BRCA1 promoter - Inhibited the migratory ability of TNBC cell lines and regulated genes involved in tumor migration - Downregulated the T47D cells in BRCA1 ⇒ Quercetin may induce anticancer activity against TNBC cells by modulating tumor suppressor genes	[217]
Quercetin (Purchased)	Human leukemia HL-60 cells	- Induced the apoptosis of human leukemia HL-60 cells in a dose-dependent manner - Induced the Fas ligand (FasL) expression involving activation of the extracellular signal-regulated kinase (ERK) and Jun N-terminus kinase (JNK) signaling pathways - Increased the histone H3 acetylation which resulted in the promotion of the expression of FasL - Activated the HAT and inhibited the HDAC ⇒ Quercetin induces FasL-related apoptosis by transactivation through the activation of c-jun/AP-1 and the promotion of histone H3 acetylation in HL-60 cells	[218]
Quercetin (Purchased)	SNU719 cells, a gastric carcinoma cell line	- Induced the apoptosis of SNU719 cells - Eliminated DNMT1 and DNMT3A expressions - Affected the cell cycle progression of SNU719 - Induced the signal transductions to stimulate apoptosis, and induce EBV (Epstein-Barr virus) gene transcription - Enhanced the frequency of F promoter use ⇒ Quercetin induces apoptosis through epigenic regulations	[219]
Quercetin (Purchased)	Male ICR mice	- Inhibited the nickel-induced liver injury - Decreased the total DNMT activity and Nrf2 DNA methylation in the livers of mice exposed to nickel - Induced the Nrf2 nuclear translocation and heme oxygenase-1 (HO-1) activity ⇒ Quercetin activity is associated with its ability to modulate Nrf2/HO-1 and p38/STAT1/NF-κB signaling pathways	[220]
Quercetin (Purchased)	Male Syrian hamsters	- Reduced tumor incidence and tumor burden - Induced a significant tumor growth delay - Induced the cell cycle arrest and apoptosis and blocked invasion and angiogenesis - Inhibited the HDAC-1 and DNMT1 activity ⇒ The inhibition of HDAC-1 and DNMT1 by quercetin could be behind its anticancer properties	[221]
Quercetin (Purchased)	*Mouse* intestinal epithelial cell *line* (*MODE*-*K*)	- Inhibited the TNF-induced interferon-γ-inducible protein 10 (IP-10) and macrophage inflammatory protein 2 (MIP-2) expression - No inhibition of NF-κB RelA phosphorylation and NF-κB reporter gene activity - Inhibited the TNF-induced NF-κB and cofactor recruitment to the IP-10 and MIP-2 gene promoters - Inhibited the IP-10 and MIP-2 gene expression in primary ileal IEC from inflamed TNFDARE/WT mice ⇒ The anti-inflammatory effect of quercetin in epithelial cells is mediated through the inhibition of cofactor recruitment at the chromatin of proinflammatory genes	[222]
Quercetin (Not reported)	Human prostate cancer PC3 and LNCaP cell lines	- Inhibited DNMT, causing global hypomethylation, restoring AR (Androgen Receptor) mRNA and protein levels and causing apoptosis via mitochondrial depolarization of PC3 and DU145 - Increased the AR mRNA levels 1.5-2.0 fold in PC3 and DU145 cells - Increased the AR mRNA levels by approximately 3-fold in both the cell lines - Induced the apoptosis in a significant number of cells - Induced apoptosis through mitochondrial depolarization in 34% PC3 cells and 28% DU145 cells in 48 h - Induced the expression of AR in androgen refractory PC3 and DU145-gene reporter assay - Increased the AR protein levels in PC3 and DU145 cells and remained unchanged after transfection of these cells with scrambled ARsiRNA ⇒ Quercetin has potential for use in chemoprevention of androgen resistance in prostate cancer.	[223]
Quercetin (Purchased)	Human esophageal 9706 cancer cell	- Suppressed the human esophageal 9706 cancer cell growth in a dose-dependent manner - Inhibited the Eca9706 cell proliferation - Downregulated the reverse expressions of global DNMT1, NF-κBp65, HDAC1, and Cyclin D1 - Upregulated the expressions of caspase-3 and *p16^INK4α^* - Displayed a reverse effect targeting both altered DNA methylation and histone acetylation, acting as HDAC inhibitor mediated via epigenetic-NF-κB cascade signaling ⇒ Quercetin displays a reverse effect targeting both altered DNA methylation and histone acetylation, acting as histone deacetylase inhibitor mediated via epigenetic-NF-κB cascade signaling	[224]
**Isoflavones**			
Biochanin A (Not reported)	Esophageal KYSE 510 and KYSE 150 cell lines	- Inhibited DNMT activity - Reactivated RARβ - Inhibited cancer cell growth ⇒ Biochanin A reactivates methylation-silenced genes, partially through a direct inhibition of DNA methyltransferase, which may contribute to its chemopreventive activity	[225]
Biochanin A (Purchased)	Daphnia magna Straus (clone K6)	⇒ The isoflavones biochanin A do not induce an effect on overall D. magna DNA methylation at exposure concentrations for which effects on reproduction were observed	[226]
Daidzein (Purchased)	Breast cancer cell lines, MCF-7 and MDA-MB 231	- Decreased trimethylation at H3K9 - Decreased the H3K27me3 marks - Lost methylation at K4 of H3 in MCF7 and MDA-MB 231 cell lines - Increased acetylation at H4K8 and H3K4 ⇒ Daidzein tends to modify transcription through the demethylation and acetylation of histones in breast cancer cell lines.	[227]
Daidzein (Purchased)	Prostate cell lines (PC-3, DU-145, LNCaP	- Demethylated the CpG island in the promoter of *GSTP1* in PC-3 cells - Demethylated the *GSTP1* promoter in the LNCaP line - Demethylated the CpG island of the promoter of *EPHB2* in the PC-3 line - Demethylated the CpG islands of the promoter of *RASSF1A* in the LNCaP line - Increased the nuclear expression of *GSTP1* in line DU-145 - Increased the expression of *EPHB2* in the cytoplasm of DU-145 cells ⇒ Epigenetic modifications of DNA, such as the promoter CpG island demethylation of tumor suppressor genes, might be related to the protective effect of daidzein on prostate cancer	[228]
Daidzein (Not reported)	Esophageal KYSE 510 and KYSE 150 cell lines	- Inhibited DNMT activity - Reactivated RARβ - Inhibited cancer cell growth ⇒ Daidzein reactivates methylation-silenced genes, partially through a direct inhibition of DNA methyltransferase, which may contribute to its chemopreventive activity	[225]
Equol (Purchased)	Breast cancer cell lines, MCF-7 and MDA-MB 231	- Decreased trimethylation at H3K9 - Decreased the H3K27me3 marks - Lost methylation at K4 of H3 in MCF7 and MDA-MB 231 cell lines - Increased acetylation at H4K8 and H3K4 ⇒ Equol tends to modify transcription through the demethylation and acetylation of histones in breast cancer cell lines.	[227]
Genistein (Not reported)	Esophageal KYSE 510 and KYSE 150 cell lines	- Reversed DNA hypermethylation - Reactivated *RARβ*, *p16^INK4a^*, and *MGMT* in KYSE 510 cells - Reversed DNA hypermethylation and reactivate RARβ in KYSE 150 cells and prostate cancer LNCaP and PC3 cells - Inhibited cell growth - Enhanced (in combination with trichostatin, SFN or 5-aza-dCyd) gene reactivation and inhibition of cell growth - Inhibited DNMT1 activity in a dose dependent manner, showing substrate- and methyl donor-dependent inhibition - Inhibited HDAC activity (13.2%) ⇒ Genistein reactivates methylation-silenced genes, partially through a direct inhibition of DNA methyltransferase, which may contribute to its chemopreventive activity	[225]
Genistein (Purchased)	breast cancer cell lines, MCF-7 and MDA-MB 231	- Decreased trimethylation at H3K9 - Decreased the H3K27me3 marks - Lost methylation at K4 of H3 in MCF7 and MDA-MB 231 cell lines - Increased acetylation at H4K8 and H3K4 ⇒ Genistein tends to modify transcription through the demethylation and acetylation of histones in breast cancer cell lines	[227]
Genistein (Purchased)	Prostate cell lines (PC-3, DU-145, LNCaP	- Partial demethylation of the CpG island in the promoter of *GSTP1* in all cell lines - Demethylated the CpG island of the promoter of *EPHB2* in the PC-3 line - Demethylated the CpG islands of the promoter of *RASSF1A* in the LNCaP line - Increased the nuclear expression of *GSTP1* in line DU-145 - Increased the expression of *EPHB2* in the nucleus of line PC-3 and the cytoplasm of DU-145 cells ⇒ Epigenetic modifications of DNA, such as the promoter CpG island demethylation of tumor suppressor genes, might be related to the protective effect of genistein on prostate cancer	[228]

#### 5.1.6. Biflavonoids

Bioflavonoids are a class of flavonoids, which consist of flavonoid dimers formed by the covalent bond (C-C or C-O-C) between two monoflavonoids. They are also secondary metabolites but with a more restricted presence in plants, serving as chemotaxonomic markers for several species. An array of biological activities, largely overlapping with those of flavonoids, has been reported for bioflavonoids and biflavonoid-enriched preparations from plants. Among them, antioxidant, antiproliferative, or anti-inflammatory activities appear prominent, suggesting their potential for pharmacological application in the prevention or treatment of atherosclerosis and associated vascular diseases [229].

#### 5.1.7. Amentoflavone

Amentoflavone is a biflavonoid (bis-apigenin coupled at 8 and 3’ positions, or 3′,8′′-biapigenin) found in plants such as *Ginkgo biloba*, *Chamaecyparis obtusa*, *Hypericum perforatum*, and *Xerophyta plicata*. Since it is an active regulator of the enzymes CYP3A4 and CYP2C9, which are responsible for the digestion of certain drugs in the body, amentoflavone can interfere with certain medicines [230].

Zhaohui et al. [144] were interested by the amentoflavone from *Selaginella tamariscina*, a widely known Chinese herbal medicinal plant with antineoplastic proprieties. They conducted detailed research into malignant glioma, a very aggressive and invasive primary brain tumor that frequently occurs in adults, using various experimental approaches to show that miR-124-3p expression was significantly downregulated in glioma tissue compared to normal brain tissue following amentoflavone treatment. It has also been reported to decrease cell viability and induce apoptosis in both glioma cell lines in a dose-dependent manner and inhibit glycolysis in glioma cells by upregulating miR-124-3p. Furthermore, amentoflavone upregulated miR-124-3p by repressing DNMT1 through Sp1, which in turn was caused by the activation of the ROS/AMPK signaling pathway by amentoflavone.

#### 5.1.8. Flavans

Flavans are abundant in nature and are the product of a double reduction in a flavanone. Many natural flavans are lipid-soluble and can be found in fruit peel as well as the cutin of leaf surfaces. A variety of flavans are phytoalexins, which give plant tissues resistance to fungi or insects. Flavans are present in higher quantities in immature fruits than in mature ones. The essential flavonoid skeleton is found in flavans, a vast group of naturally occurring compounds [231]. Isolation of a new prenylflavan. Kazinol Q from the Chinese crude medication *Broussonetia kazinoki*, has been identified [232].

#### 5.1.9. Kazinol Q

In their search for DNMT inhibitors from Formosan plants, Weng et al. [145] were interested in kazinol Q. It was shown that this molecule could act as a DNMT1 inhibitor (IC_50_ = 7 mM). Kazinol Q anticancer activity was tested against different cell lines through different mechanisms. Against MDA-MB-231 (resistant breast cancer), its activity was mediated by the activation of the genes responsible for the silenced expression of DNA methylation. Against LNCap (prostate) and MCF7 (breast) cell lines, kazinol Q induced apoptosis. Further molecular docking studies also suggest that the inhibition of DNMT activity is possibly mediated by competition with cytosine binding.

This naturally occurring compound exhibits several desirable features for drug development, including chemical stability and increased hydrophobicity, and might have therapeutic relevance for cancer treatment.

#### 5.1.10. Flavanols

Chocolate, cocoa powder, grapes, and teas contain significant amounts of flavanols, specifically monomeric flavanols (catechin, epicatechin, epigallocatechin, gallocatechin, and their gallate derivatives) and their polymerization products (proanthocyanidine) [233]; they are well-known antioxidant molecules that can contribute to other biological activities and affect cell signaling pathways [234].

#### 5.1.11. Catechin, Epicatechin, and (-)-epigallocatechin-3-O-gallate

The actions of some tea catechins on DNA methylation catalyzed by prokaryotic SssI DNMT and human DNMT1 were investigated by Lee et al. [146]. SssI DNMT- and DNMT1-mediated DNA methylation has been shown to be inhibited by each of the tea polyphenols [catechin, epicatechin, and (-)-epigallocatechin-3-O-gallate (EGCG)] in a concentration-dependent manner, EGCG being the most active inhibitor. As epicatechin was used as a model inhibitor, kinetic testing revealed that this catechol-containing dietary polyphenol prevented enzymatic DNA methylation in vitro by increasing the formation of S-adenosyl-L-homocysteine (an active, non-competitive inhibitor of (DNMTs) during catechol-O-methyltransferase-mediated methylation of this dietary catechol. The inhibitory power of EGCG on DNA methylation was mediated by DNMT, on the other hand, was independent of its methylation and was primarily attributed to its clear inhibition of DNMT. The modeling data on the precise molecular mode of EGCG’s inhibitory interaction with human DNMT1 agrees well with their experimental findings.

#### 5.1.12. (-)-epigallocatechin-3-O-gallate

**Figure 5:**
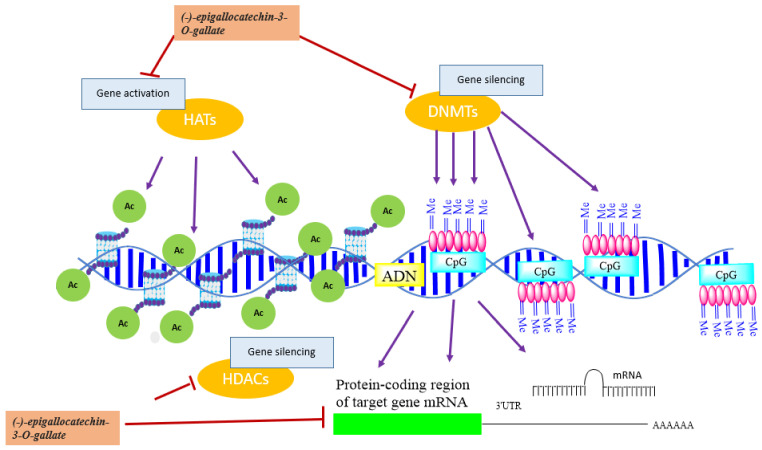
Targeted checkpoint of (-)-epigallocatechin-3-O-gallate in cancer disease. *EGCG exhibits important anticancer properties via different inhibitory actions of epigenetic enzymes including DNMT, HAT, HDAC, and miRNA.* Abbreviations: DNMT: DNA methyltransferase; HAT: histone acetyltransferase; HDAC: histone deacetylase; miRNA: micro-RNA.

According to Zhang et al. [171], EGCG could resensitize NSCLC cells to cisplatin (DDP) by demethylating candidate genes. In A549/DDP cells, EGCG + DDP treatment significantly induced proliferation inhibition, cell cycle arrest in G_1_, increased apoptosis, inhibition of DNMT and HDAC activity (Figure 5), a reversal of hypermethylated status, and a downregulation of the expression of GAS1, TIMP4, and ICAM1. In addition, in vivo pre-treatment with EGCG followed by DDP resulted in considerable tumor suppression. Methylation levels of GAS1, TIMP4, ICAM1, and WISP2 were reduced, and their expression levels were elevated in the EGCG-treated groups, but only the combinatorial treatment group inhibited growth. All of these findings indicate that pretreatment with EGCG resensitizes cells to DDP, as well as demethylation and the restoration of candidate gene expression [171].

Mittal et al. [147] investigated a cream-based formulation for topical treatment using hydrophilic cream as a vehicle for EGCG, based on previous studies that indicated a possible anti-photocarcinogenic action of ()-Epigallocatechin-3-gallate (EGCG). In an SKH-1 hairless mouse model, researchers found that treatment with EGCG (approximately 1 mg/cm^2^ skin surface) by a hydrophilic cream offered exceptionally good protection against photocarcinogenesis in terms of incidence, multiplicity and size of tumors. EGCG also inhibited the malignant transformation of UVB-induced papillomas to carcinomas by inhibiting the global DNA hypomethylation pattern caused by UVB. Long-term treatment with EGCG did not appear to cause toxicity in animals with respect to skin appearance, lean body mass, total bone mineral content, or total bone mineral density, but it did cause a decrease in fat mass when examined by dual-energy X-ray absorptiometry [147].

Chuang et al. [148] in their study tried to compare the well-characterized nucleoside analogue methylation inhibitor 5-aza-2V-deoxycytidine (5-Aza-CdR) with three non-nucleoside demethylating agents: ()-epigallocatechin-3-gallate, hydralazine, and procainamide. The results of this study indicate that 5-Aza-CdR is much more effective in DNA methylation inhibition as well as in reactivating genes, compared to non-nucleoside inhibitors.

The objective of the research of Fang et al. [149] was to determine the impact of DNA hypermethylation on the loss of O-6-methylguanine-DNA methyltransferase (MGMT) expression throughout the progression of ESCC and to evaluate its reactivation in cell lines. Their findings suggest that dietary polyphenols (-)-epigallocatechin-3-gallate and genistein reactivate the MGMT gene, implying that MGMT gene hypermethylation is an important mechanism for MGMT gene silencing in the development of ESCC and that this epigenetic event may be prevented or reversed by these polyphenols to prevent carcinogenesis [149].

Chen et al. [168] performed genome-wide methylation and mRNA expression screen in the CAL-27 cell line with and without EGCG (100 M) treatment to gain a better understanding of the mechanisms underlying the effect of EGCG on DNA methylation and its chemopreventive action in oral squamous cell carcinoma (OSCC). Following treatment with EGCG, a total of 761 differentially methylated gene loci were identified. When comparing gene expression profiles in OSCC samples to controls, 184 transcripts showed a significant difference (*p* < 0.05) and a fold change difference >2. The main pathways involved in this study were metabolic, MAPK, wnt, and cell cycle pathways, according to gene ontology analysis of differentially methylated loci and functional annotation of differentially expressed genes. This study also indicated that EGCG could affect the methylation status and gene expression in the CAL-27 cell line. Changes in various critical signaling pathways might also disclose EGCG’s anticancer action.

In HeLa cells, Khan et al. [169] investigated the impact of EGCG, as an epigenetic modulator, based on growing data supporting the importance of bioactive dietary compounds in preventing carcinogenesis by targeting epigenetic changes such as DNA methylation. In this research, the enzymatic activity of DNMT and HDAC was found to be significantly reduced in HeLa cells treated with EGCG (Figure 5). However, DNMT3B expression was dramatically reduced in a time-dependent manner, although HDAC1 expression did not alter appreciably [169]. The suppression of DNMT3B and HDAC1 activity by EGCG was further validated by molecular modeling results. Furthermore, due to significant changes in the methylation of promoter regions of known tumor-suppressor genes (TSGs), exposure to EGCG over time resulted in the reactivation of known TSGs in HeLa cells. Overall, the authors’ findings show that EGCG might have a major influence on the development of new epigenetic-based therapies [169].

Tyagi et al. [170] conducted a study to assess the therapeutic efficacy of a combination of non-toxic, low dose of 5-aza 2′ dC with EGCG on growth inhibition of breast cancer cells, taking into account the limited results in the treatment of breast cancer using DNA demethylating agent 5-aza-2′-deoxycytidine (5-aza 2′dC) and its toxicity towards normal cells. Research found that co-treatment with 5-aza 2′ dC and EGCG inhibited the proliferation of breast cancer cells much more than individual therapies, with no detectable toxicity in MCF-10A cells [170]. In combination-treated cells, changes in DNA methylation and alterations in histones were also more pronounced. The results of this work revealed that epigenetic mechanisms are involving in the potentiation of growth inhibition of breast cancer cells by a combination therapy of 5-aza 2′ dC and EGCG, at least in part [170].

EGCG from *Camellia sinensis* has been shown to have a variety of anti-cancer effects; this is why Borutinskaitė et al. [172] chose to investigate its ability to reduce APL cell growth and induce apoptosis. The RT-qPCR method revealed that EGCG triggered anti- cancer epigenetic alterations, including downregulation of epigenetic regulators DNMT1, HDAC1, HDAC2, and G9a. The downregulation of G9a was validated by a lower quantity of H3K9me2 following treatment with EGCG. The key components of the Polycomb repressive complex 2 (PRC2) were also shown to be downregulated at the gene and protein levels. EGCG treatment increased hyperacetylated H4 and acetylated H3K14 histones binding to the promoter regions of p27, PCAF, C/EBPa, and C/EBPE, but decreased binding to PRC2 core component genes EZH2, SUZ12, and EED, according to chromatin immunoprecipitation (ChIP) assay. These findings show that EGCG, as a cell growth inhibitor and epigenetic modulator, might be beneficial in the treatment of APL [172].

In their study, Lewis et al. [173] examined the effects of two epigenetic-modifying compounds (suberoylanilide hydroxamic acid (SAHA) and EGCG) and their combination on growth potential indicators in different cell lines of TNBC. In addition to estrogen receptor alpha (ER), these chemical compounds influenced the expression of the oncogenic miR-221/222 and the tumor suppressors p27 and PTEN. E-cadherin expression was higher, whereas N-cadherin was lower, suggesting a more epithelial phenotype. Furthermore, the therapies reduced the activity of DNMTs, and there was a considerable enrichment of AcH3 inside the promoters of p27 and PTEN, suggesting that epigenetic processes are involved in the aforementioned modifications. These results confirm the importance of SAHA and EGCG in suppressing breast cancer cell growth and proliferation.

Yamada et al. [150], based on a study by Fang et al., who found that the green tea polyphenol constituents—catechins—inhibit DNMT1, suppress DNA methylation, and re-express the mRNA and protein of four genes in various human cancer cell lines. The authors also tested the effect of EGCG supplied to mice in order to observe its effect on DNA methylation in the promoter region of the estrogen receptor gene of wild type and knockout mice [150]. The methylation rate in the EGCG-administered animals decreased by 4% in the wild-type mice and 5% in the knock-out mice. According to this research, EGCG may have an inhibitory impact on epigenetic alterations linked to carcinogenesis or aging. The ratio of concentrations causing in vitro cytotoxicity to the optimal EGCG dosage has also been shown to be approximately five times greater than that of zebularine, although the optimal EGCG dosage is 100 times lower. These findings suggest that EGCG, or green tea, may be a promising option for cancer prevention, anti-aging, or cancer treatment with minimal side effects.

Stresemann et al. [2] carried out a comparative investigation of the six most widely known DNMT inhibitors at the time; 5-azacytidine (5-aza-CR), 5-aza-2V-deoxycytidine (5-aza-CdR), zebularine, procaine, EGCG, and RG108. In human cancer cell lines, 5-aza-CR, 5-aza-CdR, zebularine, and EGCG were reported to have considerable cytotoxicity. 5-aza-CdR and EGCG were also proven to be genotoxic by the induction of micronuclei. Furthermore, 5-aza-CR, 5-aza-CdR, zebularine, and RG108 demethylated genomic DNA in a concentration-dependent manner, but procaine and EGCG had no impact.

Berletch et al. [151] investigated the inhibitory effects of EGCG on hTERT (the essential catalytic subunit for the proper function of telomerase, expressed in approximately 90% of all cancers) in MCF-7 breast cancer cells and HL60 promyelocytic leukemia cells. In vitro, EGCG treatment reduced cellular proliferation and triggered apoptosis in MCF-7 and HL60 cells. However, the reduction in hTERT mRNA expression was proved only inMCF-7 cells [151]. Furthermore, epigenetic changes appeared to play an important role in downregulating hTERT gene expression in MCF-7 cells. EGCG treatment of MCF-7 cells reduced hTERT promoter methylation and blocked histoneH3 Lys9 acetylation in a time-dependent manner. Further study revealed an increase in hTERT repressor E2F-1 binding to the promoter in combination with demethylation. These data suggest that EGCG is effective in inducing cell death in MCF-7 and HL60 cancer cell lines via various mechanisms, including antioxidant effects and epigenetic modification [151].

Kato et al. [152] conducted a study to investigate the effect of EGCG on the methylation status of the RECK gene (tumor suppressor gene that negatively regulates matrix metalloproteinases (MMPs) and inhibits tumor invasion, angiogenesis, and metastasis) and cancer invasion in OSCC cell lines. Their findings show that EGCG therapy of oral cancer cells partly reverses the hypermethylation status of the RECK gene and greatly increases RECK mRNA expression. After treatment with EGCG, the levels of MMP-2 and MMP-9 were likewise reduced in these cells [152]. In a three-dimensional collagen invasion model, EGCG dramatically reduced cancer cell invasive ability by lowering the number of invasive foci (Po0.0001) and invasion depth (Po0.005). These findings support the idea that EGCG suppresses cell invasion via numerous mechanisms, including a demethylation impact on MMP inhibitors like RECK [152].

Balasubramanian et al. [153] were interested in learning more about the influence of (2)-epigallocatechin-3-gallate on the function of two important PcG proteins regulators, Bmi-1 and enhancer of zeste homolog 2(Ezh2). Both Bmi-1 and Ezh2 levels were lowered in SCC-13 cells after treatment with EGCG, which was related to decreased cell survival. A global reduction in histone H3 lysine 27 trimethylation, a hallmark of PRC2 complex function, was associated with decreased survival. This change in PcG protein expression was linked to decreased expression of key proteins that promote cell cycle progression [cyclin-dependent kinase (cdk)1, cdk2, cdk4, cyclin D1, cyclin E, cyclin A, and cyclin B1] and increased expression of proteins that inhibit cell cycle progression [cdk1, cdk2, cdk4, cyclin D1, and cyclin (p21 and p27)]. The increase in caspase 9, 8, and 3 cleavages, as well as the increase in poly(adenosine diphosphate ribose) polymerase (PARP) cleavage, indicated that apoptosis was intensified. Treatment with EGCG also increases Bax and suppresses Bcl-xL expression. These EGCG-dependent alterations are reversed by vector-mediated increased Bmi-1 expression. According to the findings, green tea polyphenols decrease skin tumor cell survival via affecting PcG-mediated epigenetic regulatory pathways [153].

Using aberrant promoter methylation of Wnt inhibitory factor-1 (WIF-1) as a model for epigenetic silencing in human cancers, Gao et al. [154] investigated the ability of EGCG to influence promoter demethylation and reactivation, as it has been shown to directly reactivate several methylation-silenced genes. In the H460 and A549 cell lines, EGCG administration resulted in promoter demethylation of WIF-1 and restoration of WIF-1 expression. EGCG significantly reduced cytosolic catenin protein levels and hindered Tcf/Lef reporter activity, indicating that EGCG might be used to reverse WIF-1 promoter methylation in the future [154].

In their study, Medina-Franco et al. [155] attempted to provide experimental evidence that two of the most well-known natural products associated with DNA methylation inhibition, EGCG and curcumin, have little or no pharmacologically relevant inhibitory activity against DNMTs.

The researchers used a verified homology model of DNMT1’s catalytic domain to perform a virtual screening of a huge library of natural compounds. The virtual screening used three docking programs and a multistep docking technique to focus on a track as a fraction of natural compounds docked with DNMT1 [155]. The lead-like fraction was characterized in terms of chemical space coverage and scaffold composition before docking. All three docking tools found consensus hits with strong projected docking affinity for DNMT1. In a prior investigation, one hit was shown to have DNMT1 inhibitory action. The virtual screening hits were found in the biologically relevant chemical space of medicines, and they might be one-of-a-kind natural DNMT inhibitors [155].

Meeran et al. [156] found that treatments with EGCG and a new prodrug of EGCG (pro-EGCG or pEGCG) suppressed the growth of human breast cancer MCF-7 and MDA-MB-231 cells, but not normal control MCF10A cells, in a dose- and time-dependent manner. In ER-positive MCF-7 and ER-negative MDA-MB-231 cells, both EGCG and pro-EGCG inhibited the transcription of hTERT, the catalytic subunit of telomerase, through epigenetic processes. Downregulation of hTERT expression was discovered to be caused by hypomethylation and deacetylation of the hTERT promoter, which was mediated at least in part by suppression of DNMT and HAT activities, respectively. Furthermore, they discovered that EGCG and pEGCG may change the chromatin structure of the hTERT promoter by lowering the levels of acetyl-H3, acetyl-H3K9, and acetyl-H4. Both EGCG and pEGCG caused chromatin changes that made it easier for hTERT repressors such MAD1 and E2F-1 to bind to the hTERT regulatory region. In ER-positive and ER-negative breast cancer cells, siRNA depletion of E2F-1 and MAD1 restored the pEGCG downregulation of hTERT expression and related cellular death in a distinct way. Through epigenetic control of telomerase, utilizing pro-EGCG, a more stable version of EGCG, as a potential chemopreventive agent might bring new insights into breast cancer prevention.

Various data suggested that dietary phytochemicals may alter cancer risk in cells by modifying epigenetic processes. Indeed, Nandakumar et al. [157] conducted their study to see whether tea catechins, specifically EGCG, would modify epigenetic events to regulate DNA methylation-silenced tumor suppressor genes in skin cancer cells (human epidermoid carcinoma A431 cells). According to the findings, EGCG administration reduced global DNA methylation levels in A431 cells in a dose-dependent manner [157]. The following were all reduced by ECGC treatment: 5-methylcytosine levels, DNMT activity, mRNA, and protein levels of DNMT1, DNMT3a, and DNMT3b. EGCG reduced HDAC activity and enhanced acetylated lysine 9 and 14 on histone H3 (H3-Lys 9 and 14), as well as acetylated lysine 5, 12, and 16 on histone H4, but decreased methylation H3-Lys 9. The mRNA and proteins of the silenced tumor suppressor genes *p16^INK4a^* and Cip1/p21 were also re-expressed after EGCG therapy [157]. This research adds to our understanding of EGCG’s epigenetic mode of action, which might aid in the chemoprevention of skin cancer and have substantial implications for epigenetic treatment [157].

To better understand the particular toxicity of EGCG towards cancer cells, Min et al. [158] carried out research to characterize the process of carcinogenesis and to discover excellent cancer chemotherapeutic drugs. They compared the effects of EGCG-induced ROS on hTERT-mediated apoptosis in normal and cancer cells. The amounts of ROS generation by EGCG differed across cell types; ROS, particularly hydrogen peroxide, were strongly produced in cancer cells but not in normal cells. Furthermore, greater ROS levels inhibited hTERT expression by the CCCTC-binding factor (CTCF) to the core promoter region of hTERT, suppressing hTERT expression. CTCF binding was epigenetically regulated by the demethylation of the previously hypermethylated CTCF site, which was caused by DNMT1 downregulation [158]. On the other hand, normal cells did not show any hTERT downregulation. The findings of this research revealed that EGCG-induced cancer cell death is mediated by the cancer-specific production of ROS and epigenetic modification of the expression of apoptosis-related genes such as hTERT [158].

In earlier epidemiological studies, green tea and cruciferous vegetables were related to a decreased risk of ovarian cancer. Accordingly, Chen et al. [160] hypothesized that determining the effects and processes of key components of green tea (EGCG) and cruciferous vegetables (sulforaphane, SFN) on ovarian cancer cells could provide crucial information for developing novel treatments for the disease. In their study, EGCG or SFN were used to treat both paclitaxel-sensitive (SKOV3-ip1) and paclitaxel-resistant (SKOV3TR-ip2) ovarian cancer cell lines separately or in combination, and they discovered that SFN inhibits cell viability of both ovarian cancer cell lines in a time- and dose-dependent manner and that EGCG potentiates the inhibitory effect of SFN on ovarian cancer cells [160]. SFN can arrest ovarian cancer cells in the G_2_/M phase, whereas EGCG and SFN co-treatment may arrest cells in both the G_2_/M and S phases, according to cell cycle studies. After six days of therapy, a combination of EGCG and SFN enhances apoptosis in paclitaxel-resistant SKOV3TR-ip2 cells while decreasing the expression of hTERT, the key regulatory component of telomerase. SFN may also reduce the protein levels of Bcl-2 (an anti-apoptosis gene) in both cell types, according to Western blotting. After six days of treatment with SFN, cleaved PARP is upregulated, and this is much more prominent for combined therapy, suggesting apoptotic induction [160]. Furthermore, after six days of therapy with SFN alone, phosphorylated H2AX is upregulated, and EGCG may enhance this effect.

Since epigenetic gene silencing involving DNMTs and HDACs plays an important role in colon cancer progression, Moseley et al. [161] investigated the role of EGCG in combination with other DNMT and HDAC inhibitors in this type of cancer, which at the time was the second leading cause of cancer death in the United States. According to this study, when methylation-sensitive HCT 116 human colon cancer cells were treated with EGCG, HDAC, and DNMT, protein production decreased, but remained rather stable in the HT-29 cell line. The fact that DNMT3A and HDAC3 are degraded in methylation-sensitive colon cancer cells in part via blocking their connection with the E3 ubiquitin ligase UHRF1 could explain this decrease in expression. The results of this research support the development of a methylation-sensitive colon cancer-targeted treatment that might incorporate EGCG in conjunction with other DNMT and HDAC inhibitors [161].

Mirza et al. [162] hypothesized that natural substances alter methylation-mediated gene silencing by modulating the expression of DNMTs and their associated proteins in breast cancer cell lines. Increased DNMT transcript expression, including DNMT1, DNMT3a, and DNMT3b, in breast cancer tissues, suggests that DNMTs are involved in breast carcinogenesis [162]. According to quantitative RT-PCR analysis, treatment with natural substances, such as EGCG, genistein, withaferin A, curcumin, resveratrol, and guggulsterone resulted in a substantial reduction in the transcript levels of all investigated DNMTs. Moreover, the expression of epigenetic modulators (DNMT1, HDAC1, and MeCP2) was importantly reduced by these natural substances. The natural substances EGCG, genistein, withaferin A, curcumin, resveratrol, and guggulsterone have the ability to reverse epigenetic alterations. Furthermore, because of their low toxicity, these natural chemicals are attractive candidates for breast cancer chemoprevention. However, future mechanistic studies to elucidate how these chemicals regulate gene expression are required.

According to some studies, food and environmental factors have a direct impact on epigenetic pathways linked to cancer development. Indeed, in the human colon carcinoma cell line HT29, Groh et al. [163] investigated the impact of various polyphenols on the suppression of HDAC activity and the disruption of the HDAC complex, since they were identified as a powerful method for cancer treatment and chemoprevention [163]. HDAC activity was inhibited in intact HT29 cells by the polyphenols; EGCG and genistein (GEN), as well as by two oxidative methyleugenol (ME) metabolites. In addition, following incubation with EGCG and GEN, a substantial drop in HDAC1 protein level was detected, but the examined ME metabolites did not affect HDAC1 protein status. These dietary components were found to have potential HDAC-inhibitory characteristics, which contributed to epigenetic modifications in colon carcinoma cells, and should be included in future risk/benefit analyses of polyphenols and alkenylbenzenes.

Several investigations showed that the combination of agents, such as nucleoside analogues with dietary phytochemicals, may boost the therapeutic efficacy in cancer cell epigenetic reprogramming. Indeed, Lubecka et al. [174] investigated the effects of combining ClF (at IC_50_ concentration) with EGCG (tea catechin) or genistein (soy phytoestrogen) at physiological concentrations on breast cancer cell growth, apoptosis, and epigenetic regulation of retinoic acid receptor beta (RARB) transcriptional activity in MCF7 and MDA-MB-231 cells. Combinatorial exposures inhibited breast cancer cell growth and induced death, followed by RARB hypomethylation and numerous increases in RARB, PTEN, and CDKN1A transcript levels. These findings showed that ClF-based combinations with polyphenols may accelerate cancer cell death and reactivate DNA methylation-silenced tumor suppressor genes in breast cancer cells with varying levels of invasiveness [174].

Pal et al. [175] tested the anticancer activity of EGCG (a primary component of green tea polyphenol), eugenol (an active component of clove), and amarogentin (an active component of chirata plant) in the Hela cell line, either alone or in a combination. Consequently, EGCG combined with eugenol–amrogentin inhibited cell proliferation and colony formation more effectively than therapies alone. Apoptosis induction was also greater following treatment with EGCG in combination with eugenol–amrogentin than following treatment with one of the drugs alone. At the G_1_/S phase of the cell cycle, these drugs inhibited proliferation by downregulating cyclinD1 and upregulating cell cycle inhibitors LIMD1, RBSP3, and p16. As a consequence of the lower expression of DNMT1, treatment with these drugs may cause promoter hypomethylation of LimD1 and P16 genes (DNMT1). In the Hela cell line, the research found that EGCG combined with eugenol–amarogentin had a superior chemotherapeutic effect. This action might be related to epigenetic alteration, namely DNA hypomethylation caused by DNMT1 downregulation [175].

According to Deb et al. [177], green tea polyphenols (GTP)/EGCG mediate epigenetic reactivation of TIMP3, which plays a vital role in reducing invasiveness and cancer development. TIMP3 mRNA and protein expression were elevated in human prostate cancer DUPRO and LNCaP cells after treatment with 10 g/mL GTP and 20 M EGCG. The reduction in the expression of both enhancers of zeste homolog 2 (EZH2) and its catalytic product trimethylation of histone H3 at lysine 27 (H3K27me3) repressive marks at the TIMP3 promoter, as well as an increase in histone H3K9/18 acetylation, was linked to TIMP3 transcriptional activation. In prostate cancer cells, GTP/EGCG therapy significantly reduced class I HDAC activity/expression, as well as EZH2 and H3K27me3 levels. MMP2/MMP9 gelatinolytic activity was likewise decreased by EGCG/GTP exposure, as was the capacity for invasion and migration in these cells. Independent of DNA methylation, the silencing of EZH2 and class I HDACs significantly boosted TIMP3 expression [177]. Furthermore, clinical investigations including patients undergoing prostatectomy who consumed 800 mg EGCG (Polyphenon E) for six weeks and controls of the same grade show that plasma level of TIMP3 were increased. In GTP-supplemented prostate tissue, there was a significant decrease in class I HDAC activity/expression, as well as EZH2 and H3K27me3 levels. Therefore, TIMP3 activation, as a crucial epigenetic process controlled by green tea to restore MMP/TIMP balance, reduced prostate cancer growth [177].

Epigenetic alterations, according to Sheng et al. [178], are essential pathways that contribute to cancer development. According to the evidence, the most abundant catechin in green tea, EGCG, may inhibit carcinogenesis by targeting epigenetic changes. The aim of their research was to observe how EGCG affects SCUBE2 methylation in breast cancer cells. The results show that EGCG has a strong inhibitory impact on cell viability in a dose- and time-dependent manner, and that it has greater effects than other catechins. EGCG treatment increased SCUBE2 gene expression and E-cadherin expression, and reduced vimentin expression, resulting in the considerable inhibition of cell migration and invasion [178]. On SCUBE2 knock-down cells, the inhibitory impact of EGCG was significantly reduced. Further research revealed that EGCG reduced DNMT expression and activity, resulting in a substantial drop in SCUBE2 methylation status. In conclusion, their research revealed, for the first time, that EGCG therapy may reverse SCUBE2 methylation, thus inhibiting breast cancer growth. These findings point to EGCG’s epigenetic significance and its potential use in breast cancer treatment [178].

The goal of the Ciesielski et al. [179] investigation was to verify how EGCG affects the histone posttranslational modification machinery and chromatin remodeling in microvascular (HMEC-1) and vein (HUVECs) human endothelial cells. The authors investigated the activity (fluorometric assay kit) and gene expression (qPCR) of the enzymes that alter the human epigenome, as well as the methylation and acetylation status of histones (Western blotting). EGCG enhances histone acetylation (H3K9/14ac, H3ac) and the methylation of both active (H3K4me3) and repressive (H3K9me3) chromatin marks, according to these results [179]. In both cellular and cell-free models, catechin was discovered to be an HDAC inhibitor. EGCG also alters chromatin architecture by lowering the expression of HP1 and HP1 heterochromatin binding proteins. EGCG increases chromatin relaxation in human endothelial cells and has wide epigenetic potential, altering the expression and activity of epigenome modulators such as HDAC5 and 7, p300, CREBP, LSD1, and KMT2A [179].

In their work, Steed et al. [180] used SAHA, an HDAC inhibitor, alone or in combination with EGCG, to treat TNBC. The compounds were able to reduce cIAP2 expression while enhancing pro-apoptotic caspase 7 expression. There were also alterations in histone modifications, suggesting that epigenetic processes are involved in the changes in cIAP2 expression. Apoptosis increased as a consequence of these alterations. TNBC cell migration over a fibronectin (FN) matrix was likewise inhibited by SAHA and EGCG. TNBC’s metastatic potential is reduced by SAHA and EGCG, which induce the apoptotic pathway [180].

In their work, Wu et al. [164] examined the effects of EGCG alone or in combination with trichostatin A (TSA) on *p16^INK4a^* gene expression and proliferation in human malignant lymphoma CA46 cells. The CA46 cell proliferation was suppressed by both EGCG and TSA alone; however, the combination therapy (6 g/mL EGCG and 15 ng/mL TSA) substantially decreased CA46 cell proliferation from 24 to 96 h (all P0.001). When compared to the other groups, cells treated with 24 g/mL EGCG or the combination therapy (6 g/mL EGCG plus 15 ng/mL TSA) displayed decreased proliferative indices. The combination of EGCG and TSA reduced *p16^INK4a^* gene methylation while increasing *p16^INK4a^* mRNA and protein expression. Thus, EGCG and TSA work together to reactivate the *p16^INK4a^* gene, in part by lowering promoter methylation, which may reduce CA46 cell proliferation [164].

Utilizing PDA cells (pancreatic ductal cells), Appari et al. [165] examined the efficacy of catechins on stem cells and miRNA signaling. They were found to inhibit colony formation, with EGCG shown to be less effective than the other tested catechins. Green tea catechins (GTCs) decreased viability, cell migration, MMP-2 and MMP-9 expression, ALDH1 activity, and colony and spheroid formation, and triggered apoptosis in a dose-dependent manner. MiR-let7-a expression was elevated selectively in cancer cells but not in normal cells, and it was linked to K-ras inhibition. According to these findings, GTC inhibits PDA growth by inducing miR-let7-a and inhibiting K-ras.

Deb et al. [166] show that green tea polyphenols (GTP) and its main ingredient, EGCG, promote epigenetic regulation of TIMP-3 levels and decrease the invasiveness and gelatinolytic activity of MMP-2 and MMP-9 in breast cancer cells. Both TIMP-3 mRNA and protein levels are considerably increased in MCF-7 and MDA-MB-231 breast cancer cells after 72 h of treatment with 20 mM EGCG and 10 mg/mL GTP. The TIMP-3 suppression in breast cancer cells is mediated by epigenetic silencing mechanisms, including the increased activity of the enhancer of zeste homolog 2 (EZH2) and class I HDACs, which is independent of promoter DNA hypermethylation [166]. Both GTP and EGCG treatment of breast cancer cells drastically lowered the levels of EZH2 and class I HDAC proteins. TIMP-3 transcriptional activity was also linked to a reduction in EZH2 localization and H3K27 trimethylation enrichment at the TIMP-3 promoter, as well as an increase in histone H3K9/18 acetylation. The TIMP-3 induction is a critical epigenetic process controlled by GTPs in restoring the MMP:TIMP balance and delaying breast cancer development and invasion, according to their results [166].

Saldanha et al. [167] investigated the role of the combinatorial effects of EGCG, a predominant polyphenol in green tea, and sodium butyrate (NaB), a dietary microbial fermentation product of fiber, in the regulation of survivin, which is an overexpressed anti-apoptotic protein in colon cancer cells. Their research revealed that the combo therapy caused apoptosis and cell cycle arrest in RKO, HCT-116, and HT-29 colorectal cancer cells for the first time. In all three cell lines, this was shown to be mediated by a reduction in HDAC1, DNMT1, survivin, and HDAC activity [167]. For combinatorial therapy, RKO and HCT-116 cells showed a G_2_/M arrest, whereas HT-29 colorectal cancer cells showed a G_1_ arrest. In RKO colorectal cancer (CRC) cells, further investigation of the molecular processes showed a p53-dependent activation of p21 and an increase in NF-κ B-p65. The combo therapy caused an increase in double-strand breaks as measured by gamma-H2A histone family member X (-H2AX) protein levels and production of histone H3 hyperacetylation. Furthermore, there was a reduction in global CpG methylation [167]. These data demonstrate that combinatorial EGCG and NaB are effective in inducing apoptosis, cell cycle arrest, and DNA damage in CRC cells at low and physiologically attainable doses [167].

#### 5.1.13. Flavanones

Flavanones are a minority group in food, but they are abundant in tomatoes and oranges, as well as in some aromatic plants like mint [235]. In grapefruit, the dominant aglycone is naringenin, in grapes, hesperetin, and lemons, eriodictyol. Flavanones are mostly found in the fruit’s solid sections, especially the white spongy component, albedo, and the membranes that separate the fruit’s segments. They have received a great deal of attention because of their potential health benefits [235].

#### 5.1.14. Hesperetin

Hesperetin is the 4′-methoxy derivative of the flavanone eriodictyol. Hesperidin, hesperetin’s 7-O-glycoside, is a naturally occurring flavanon-glycoside and the primary flavonoid in lemons and sweet oranges. It is a powerful antioxidant, anti-inflammatory, and anticancer agent [236].

In their study, Hermawan et al. [181] tried to reveal the molecular mechanism that could be behind the role of hesperetin against chemoresistance. Molecular docking was used as an approach, and hesperetin results were compared with those of erbB receptor inhibitors. According to the study’s findings, both hesperetin and erbB receptor inhibitors target the same mRNA expression, suggesting the ability to inhibit ABL1, MLH1, and DNMT38.

#### 5.1.15. Hesperidin

Citrus fruits contain hesperidin, a flavanone glycoside. Hesperetin is its aglycone form. Its name is taken from the term “hesperidium,” which refers to citrus fruit. Hesperidin was discovered in the white inner layer of citrus peels. It is thought to be involved in plant protection. It has a variety of pharmacological functions, including antioxidant and anti-inflammatory properties. Nevertheless, the effective chemopreventive activity of hesperidin has been shown in pre-clinical cancer models in vitro and in vivo [237].

To investigate whether the cytotoxicity and genotoxicity of flavonoids may lead to cancer cell death, Lewinska et al. [182], in their study, selected the flavonoid hesperidin to determine its activity toward the DU145 prostate cancer cells. Hesperidin had a varied effect on cancer cell counts and proliferation. Hesperidin enhanced intracellular, total ROS, and superoxide production. This molecule was also found to induce double-strand breaks of DNA and micronuclei. Genotoxicity was discovered in DU145 cells, which resulted in pro-apoptotic activity. As a DNA hypomethylating agent, it may alter the epigenome of cancer cells, affecting gene expression patterns.

Fernández-Bedmar et al. [183] used an in vitro model to examine the methylation patterns induced by hesperidin in the HL60 cell line to investigate its chemopreventive effects in epigenetic cancer therapies. To confirm its therapeutic effectiveness, a similar in vivo pilot experiment was conducted utilizing a rat diethyl nitrosamine-induced hepatocarcinogenesis model. Hesperidin was found to be cytotoxic and exhibited a significant hypomethylating effect on the LINE-1 and ALUM2 sequences. Hesperidin did not affect the rat body and liver weight while reducing the diethylnitrosamine-induced nodules. Hesperidin could be proposed as a candidate molecule in chemoprevention for epigenetic therapy purposes.

#### 5.1.16. Naringenin

Naringenin is one of the most common flavonoids found in plants. It is abundant in citrus fruits such as orange, mandarin, and grapefruit [238]. Previous investigations showed that naringenin has a wide range of pharmacological effects, including antioxidant, anti-diabetic, and anti-inflammatory properties. It has anticancer properties since it induces apoptosis and cell cycle arrest in several cancer cell lines (MDA-MB-231, HepG2, E0771, PC3, and LNCaP). Naringenin also plays a significant function as an anti-angiogenic chemopreventive agent [239].

Yan et al. [184] explored the protective effects of naringenin on the kidney as well as its effects on let-7a/TGFBR1 signaling in diabetic nephropathy rats and mesangial cells under high glucose conditions, demonstrating that let-7a and its related pathway-TGF-*β*1/smad signaling formed a negative feedback to inhibit the deposition of ECM by targeting TGFBR1 in DN [184]. Additionally, naringenin might repress glomerular mesangial cells proliferation and ECM accumulation through let-7a/TGFBR1 signaling in DN, and let-7a may be a potential new target for the protection of naringenin against diabetic nephropathy [184].

Naringenin’s effect on the expression levels of miR-17-3p, miR-25-5p, and relative mRNA targets (related to the antioxidant and anti-inflammatory activities) was studied by Curti et al. [185]. Based on the results, it was found that naringenin could exert its antioxidant activity through epigenetic regulation (downregulation of miR-17-3p and miR-25-5p) determined by PCR on Caco-2 cells.

#### 5.1.17. Flavanonols

One of the most unexploited classes of flavonoids is that containing a flavan-3-one moiety, with a structure characterized by a 2-phenyl-3,4-dihydro-2H-1-benzopyran bearing a hydroxyl group and a ketone at the carbon C_2_ and C_3_, respectively. The main anticancer and epigenetic potential of this family was assessed through one of its typical molecules, taxfolin.

#### 5.1.18. Taxifolin

Taxifolin (3,5,7,3,4-pentahydroxy flavanone or dihydroquercetin) is a flavonoid commonly found in some fruit, onion, milk thistle, and conifers such as *Pseudotsuga taxifolia*, *Taxus chinensis*, *Cedrus deodara*, and *Pinus roxburghii* [240]. Taxifolin has long been used in clinical practice to manage cardiovascular and cerebrovascular disorders [241]. Besides the multiple pharmacological activities of taxifolin, the anticancer and chemopreventive effects were of great interest, and they were mainly attributed to its ability to modulate antioxidant response pathway proteins and inflammation in tumor micro-environments [242].

For the epigenetic potential of this molecule, Kuang et al. [186] performed an in vitro study to investigate the potential inhibitory effect of taxifolin on the neoplastic transformation of JB6 P+ cells, and to determine the underlying epigenetic mechanisms. Taxifolin was found to inhibit the TPA-induced colony formation of JB6 P+ cells and upregulate the mRNA and protein levels of Nrf2 and its downstream genes heme oxygenase-1 (HO-1) and NAD(P)H quinone oxidoreductase 1 (NQO1). It induces an antioxidant response element to (ARE)-luciferase activity in HepG2-C8 cells. Taxifolin treatment also lowers the methylation frequency of the first 15 CpGs sites in the Nrf2 promoter, according to bisulphite genomic sequencing, and blocks the development of DNMT and HDAC proteins using Western blotting analysis. These results combined indicate that taxifolin can protect against skin cancer by stimulating Nrf2 via an epigenetic pathway [186].

#### 5.1.19. Flavones

Flavones are flavonoids with a 2-phenyl-1-benzopyran-4-one skeleton. While flavones are most commonly found in herbs such as parsley and celery, they are also found in cereal grains and are the only flavonoid class found in almost all edible cereal species, including maize, wheat, rye, barley, oats, sorghum, and millets [243]. The flavones in cereals are particularly noteworthy because they have structural features (e.g., C2–C3 double bond) correlating with biological activity that increases significantly in the prevention of inflammation-linked diseases and cancer [244]. 3,6-dihydroxyflavone (3,6-DHF), apigenin, baicalein, casticin, chrysin, diosmin, isovitexin, luteolin, naringin, and pectolinarigenin are the flavones selected and studied for their epigenetic potential.

#### 5.1.20. 3,6-dihydroxyflavone (3,6-DHF)

3,6-dihydroxyflavone (3,6-DHF) is a well-known chemopreventive drug that has been used to treat a variety of malignancies, including breast cancer. 3,6-DHF has also been shown to have anti-allergic properties and to be a neuroprotective chemical in the treatment of stroke [245].

In their previous study, Peng et al. [187] identified 3,6-DHF as a promising chemopreventive agent and discovered that it significantly upregulates miR-34a and downregulates miR-21 in breast carcinogenesis, but the upstream and downstream events of the anticancer mechanism remain unknown. Scientists in this investigation found that cotreatment with 3,6-DHF substantially suppressed carcinogen-induced breast carcinogenic transformation in human breast epithelial MCF10A cells. The results of this research indicated a considerable downregulation of miR-34a and overexpression of miR-21 in breast carcinogenesis, which may be alleviated by treatment with 3,6-DHF. The studied flavone suppresses the hypermethylation of the miR-34a promoter, according to methylation-specific PCR detections. Furthermore, the study revealed that 3,6-DHF is a potent DNMT1 inhibitor, docking to the protein’s putative cytosine pocket and reducing DNMT activity in a dose-dependent manner. Furthermore, a ChIP-qPCR study of histone modifications revealed that 3,6-DHF therapy significantly reduced H3K9-14ac on the miR-21 promoter. In addition, according to the same research, 3,6-DHF inhibited the PI3K/Akt/mTOR signaling pathway in breast carcinogenesis in vitro and in vivo. The effects of 3,6-DHF on Notch-1 and PTEN were greatly decreased by inhibiting miR-34a or overexpressing miR-21, which diminished the suppression of 3,6-DHF on PI3K/Akt/mTOR. 3,6-DHF reduced the PI3K/Akt/mTOR signaling pathway in breast carcinogenesis by upregulating miR-34a via DNMT1 inhibition and hypermethylation while downregulating miR-21 through modifying histone modification [187].

The mechanism by which 3,6-dihydroxyflavone remarkably enhances miR-34a in breast carcinogenesis was investigated by Peng et al. [188]. Using MCF10A, MDA-MB-231 cells, and methylation-specific PCR assays, it was found that 3,6-dihydroxyflavone upregulates miR-34a by increasing TET1 expression (by inhibiting DNMT1 and DNA hypermethylation).

#### 5.1.21. Apigenin

Apigenin (4′,5,7-trihydroxyflavone), present in several plants, is a flavone-class natural product that serves as an aglycone for many natural glycosides. It is a yellow crystalline solid which has been used to color wool [246]. Its anticancer activity is mediated via apoptosis and cell death in lung epithelial cancer cells (A549) and causes mitochondrial death by inhibiting the expression of VEGF and HIF-1 through the pI3K/AKT/p70S6K1 and HDM2/p53 pathways [247].

To study its epigenetic regulation potential, Kanwal et al. [189] investigated the effect of apigenin in inhibiting the methyltransferase activity of DNA and histone proteins on human prostate cancer LNCaP and DU145 cells and transformed human prostate epithelial RWPE-1 cells. Epigenetic studies performed with apigenin exhibited a decrease in DNMT enzyme activity and a reversal of the hypermethylation of cytosine bases in the DNA and prevented cytosine methylation in the GCrich promoter sequence incubated with the M.SssI enzyme [189]. Furthermore, a marked decrease in HMT activity and a decrease in EZH2 protein expression and H3K27 trimethylation were noted in histones isolated from cancer cells treated with apigenin. This suggests that this molecule can alter DNMT and HMT activities and hence the methylation of DNA and histone proteins that regulate epigenetic modifications, thus providing a significant anticancer effect by altering epigenetic processes involved in the development of cancer [189].

According to a study conducted by Pandey et al. [190], apigenin-mediated growth inhibitory responses in prostate cancer cells are attributable to inhibition of class I HDACs. Treatment of PC-3 and 22Rv1 cells with apigenin (20–40 M) inhibited the activity of HDAC enzymes, in particular HDAC1 and HDAC3. This inhibition resulted in widespread acetylation of histones H3 and H4, as well as hyperacetylation of histone H3 on the *p21^waf1^* promoter. Following apigenin exposure, a similar rise in *p21^waf1^* and Bax protein and mRNA expression was observed, which is compatible with the use of the HDAC inhibitor trichostatin A. Both cancer cells experienced cell cycle arrest and apoptosis as a result of the downstream processes [190]. Oral intake of apigenin at doses of 20 and 50 g/day over eight weeks resulted in a significant decrease in tumor development, HDAC activity, and HDAC1 and HDAC3 protein expression in PC-3 xenografts in athymic nude mice. In comparison to the control group, apigenin-fed mice showed an increase in *p21^waf1^* expression. Furthermore, apigenin consumption resulted in a considerable reduction in bcl2 expression while simultaneously increasing bax and altering the bax/bcl2 ratio in favor of apoptosis. These findings show for the first time that apigenin inhibits class I HDACs, specifically HDAC1 and HDAC3, and that its exposure causes the reversal of aberrant epigenetic processes that promote cancer [190].

Paredes-Gonzalez et al. [191] conducted their study to investigate the demethylation effect of apigenin at 15 CpG sites in the Nrf2 promoter in mouse skin epidermal JB6 P+ cells and to assess the mRNA and protein expression of Nrf2 downstream gene, NQO1. Therefore, the molecule effectively regulated the hypermethylated condition of the 15 CpG sites in the Nrf2 promoter in a dose-dependent manner. Apigenin stimulated the production of mRNA and proteins and its downstream target gene, NQO1, as well as the nuclear translocation of Nrf2. It also reduced the expression of epigenetic modulators such as DNMT1, DNMT3a, and DNMT3b, as well as the expression of several HDACs (1–8). These findings revealed that apigenin, in combination with CpG demethylation and decreased DNMT and HDAC activity, can restore Nrf2 silence in skin epidermal JB6 P+ cells. Therefore, these results could lead to new therapeutic insights into the use of dietary phytochemicals to prevent skin cancer [191].

Tseng et al. [192] studied the biological processes underlying apigenin-induced cell cycle arrest in the MDA-MB-231 breast cancer cell line. Apigenin at the nonapoptotic inducing concentration decreased cell growth and triggered cell cycle arrest during the G_2_/M phase. It decreased the expression of cyclin A, cyclin B, and CDK1, which govern the G_2_ to M phase transition in the cell cycle. Apigenin also enhanced the association of *p21^WAF1/CIP1^* with proliferating cell nuclear antigen (PCNA), which suppresses cell cycle progression, and raised *p21^WAF1/CIP1^*. The molecule inhibited HDAC activity and increased the acetylation of histone H3. It also stimulated the acetylation of histone H3 in the *p21^WAF1/CIP1^* promoter region, leading to improved *p21^WAF1/CIP1^* transcription. Apigenin successfully slowed tumor development in a tumor xenograft model. There were also decreases in the amounts of cyclin A and cyclin B, as well as increases in the levels of *p21^WAF1/CIP1^* and acetylated histone H3 in these apigenin-treated tumors. For the first time, these results showed that apigenin may be used to prevent and cure breast cancer via epigenetic control [192].

#### 5.1.22. Baicalein

Baicalein (5,6,7-trihydroxyflavone) is a flavone that was discovered in the roots of *Scutellaria baicalensis*, *Scutellaria lateriflora*, *Oroxylum indicum* (Indian trumpetflower), and Thyme [248]. Baicalin aglycone has been shown to suppress some forms of lipoxygenases and may also act as an anti-inflammatory agent. It inhibits TRPC1 channel expression and hence has an antiproliferative impact on ET-1-induced proliferation of pulmonary artery smooth muscle cell proliferation [249].

Baicalein epigenetic regulation potential of gene expression in MCF7 breast cancer cells was investigated by Aluszczak et al. [137]. The anticancer activities of bacaleine suggest a possible effect as a non-nucleoside epigenetic modulator. Indeed, baicalein inhibited the DNMT activity and did not affect the methylation pattern or the expression of RASSF1A, GSTP1 or HIN1 in MCF7 cells. The global methylation of histone H3 was not affected either. The results of this study did not suggest that baicalein is an effective epigenetic modulator [137].

#### 5.1.23. Casticin

Casticin (3’, 5-dihydroxy-3, 4’, 6, 7-tetramethoxyflavone) is an active compound isolated from various plant roots, branches, leaves, fruits, and seeds. Its pharmacological effects are well established, and it has been used as an anti-hyperprolactinemia, anti-tumor, anti-inflammatory, neuroprotective, analgesic, and immunomodulatory agent. Casticin’s anticancer role has recently received a lot of attention, demonstrating its ability to target apoptosis, which is essential in cancer therapies [250].

In MGC803 gastric cancer cells, Yang et al. [193] studied the impact of casticin on RECK protein expression and mRNA levels. RECK protein expression and mRNA levels were increased following casticin treatment, while RECK promoter methylation, DNA methylation, and nuclear methylation were decreased. Casticin also caused a downregulation of the mRNA levels and DNMT1 protein expression. This study’s findings indicate that casticin has a potential antiproliferation activity of gastric cancer MGC803 by epigenetic regulation.

In another study, Li et al. [194] investigated the effect of casticin on the stemness characteristics of HCC cells and the potential mechanism. Casticin selectively reduced the viability of HCC cells but not L02 cells, repressed DNMT1 activity and expression, and increased miR-148a-3p, which indicates that the molecule activity is mediated by disrupting the reciprocal downregulation between DNMT1 and miR-148a-3p [194].

#### 5.1.24. Chrysin

Chrysin, also known as 5,7-dihydroxyflavone, is a flavone present in honey, propolis, passion flowers such as *Passiflora caerulea* and *Passiflora incarnata*, as well as *Oroxylum indicum*. It is derived from numerous species, including the blue passion flower (*Passiflora caerulea*) [251]. Chrysin has recently been shown to be a potent regulator of aromatase and human immunodeficiency virus activation in latent infection models. It has also been shown to have antioxidant and anti-inflammatory properties, as well as cancer chemopreventive action via apoptosis activation in a variety of human and rat cell types. In most cancer cells, chrysin has been shown to induce apoptosis and suppress proliferation, and is more active than other flavonoids studied on leukemia cells, where chrysin is likely to function through the activation of caspase and inactivation of Akt signaling [252].

Kanwal et al. [189] investigated the effect of chrysin in inhibiting the methyltransferase activity of DNA and histone proteins. This chemical inhibited DNMT enzyme activity, reversed the hypermethylation of cytosine bases in DNA, and prevented cytosine methylation in the GC-rich promoter region treated with M.SssI. This chemical also reduced HMT activity, EZH2 protein expression, and H3K27 trimethylation in histones extracted from cancer cells. Our findings imply that chrysin may change DNMT and HMT activity, as well as DNA and histone methylation, hence modifying the epigenetic mechanisms implicated in cancer formation.

#### 5.1.25. Diosmin

Diosmin (diosmetin 7-O-rutinoside), a flavone glycoside of diosmetin, is a phlebotonic non-prescription dietary supplement derived from citrus fruit peels for the prevention of hemorrhoids and recurrent venous diseases, mostly of the legs. It is well known to reduce venous sound by inhibiting catechol-O-degradation methyltransferase’s of norepinephrine [noradrenaline] [253].

In their first study, Lewinska et al. [182] investigated whether flavonoid-induced genotoxicity may also provoke cancer cell death. Cyto-and genotoxicity of diosmin in DU145 prostate cancer cell lines were studied. Diosmin decreased cancer cell number and proliferative activity in a different manner. Intracellular production of ROS and superoxide was increased after diosmin treatment. This compound stimulated DNA double stand breaks (DSBs) and micronuclei production. Diosmin was found to be the most potent genotoxic agent in DU145 cells, which in turn resulted in its pro-apoptotic activity. It was also found to be DNA hypomethylating agent capable of modulating the epigenome of cancer cells, leading to changes in gene expression patterns. Taken together, this dietary flavonoid glycoside was found to be active against DU145 cells by promoting genotoxic events and concomitant apoptotic cell death [182].

Following their research on diosmin epigenetic potential, Lewinska et al. [195] used various breast cancer cells (MCF-7, MDA-MB-231, and SK-BR-3) for this investigation. Using diverse approaches and assays, diosmin was found to cause G2/M cell cycle arrest, and increases in the levels of p53, p21, and p27. It also promotes apoptosis and stimulates oxidative and nitrosative stress, DNA damage, and changes in global DNA methylation patterns. Those results altogether suggest the use of diosmin as a promising candidate in cancer therapy [195].

#### 5.1.26. Isovitexin

Isovitexin (apigenin-6-C-glucoside), an isomer of vitexin, is found in plants such as bamboo, Passiflora, pigeon pea, mimosa, wheat leaves, rice hull of *Oryza sativa*, and others. Isovitexin has been shown to have antioxidant and anti-inflammatory properties. It promotes apoptotic cell death and autophagy in cancer cells through the upstream control of the caspases Bax, PARP, p-JNK, and MAPK and downstream regulation of the caspases Bcl-2 and ERK1/2 [254]. This study examined the effects of isovitexin on DNMT1 activation and miR-34a and Bcl-2 expression levels to explain the mechanism underlying isovitexin-mediated repression of proliferation and stemness [254]. In addition, the induction of apoptosis in the spheres derived from OS cells was investigated. The results indicated that isovitexin significantly repressed survival, induced apoptosis, and decreased the level of CD133, CD44, ABCG2, and ALDH1 mRNA in the spheres derived from U2OS (U2OS-SC) and MG63 cells (MG63-SC). Isovitexin further reduced the sphere formation rate of U2OS-SC and MG63-SC. Importantly, isovitexine inhibited tumor growth and reduced tumor size of U2OS-SC xenografts in nude mice, which was accompanied by decreased CD133 protein levels, elevated apoptotic index, downregulation of PCNA expression, reduced DNMT1 activity and expression, increased miR-34a and decreased Bcl-2 levels [254]. Bcl-2 has been identified to be a direct functional target of miR-34a. Furthermore, isovitexin exhibited a synergistic effect with 5-aza-2′-deoxycytidine, the miR-34a mimic or ABT-263 to repress cell survival, and induced apoptosis, downregulated CD133, CD44, ABCG2, and ALDH1 mRNA expression levels, and reduced sphere formation rates of U2OS-SC and MG63-SC cells [254].

#### 5.1.27. Luteolin 

Luteolin (3′,4′,5,7-tetrahydroxy flavone) is a flavone naturally present in glycosylated form in a variety of fruits and vegetables. Numerous studies confirmed that luteolin has antioxidant, anti-inflammatory, and neuroprotective properties. It is also widely tested for its anticancer properties in vivo and in vitro [255]. Many researchers were interested to discover the epigenetic potential of this flavone.

Indeed, Attoub et al. [197] investigated the impact of luteolin, on survival, migration, and invasion of cancer cells in vitro, and tumor growth in vivo. Luteolin was found to decrease the viability of lung (LNM35), colon (HT29), liver (HepG2), and breast (MCF7/6 and MDA-MB231-1833) cancer cells. Luteolin increased sub-G1, inhibited HDAC, and also decreased the growth of LNM35 tumor xenografts in athymic mice. These results suggest that luteolin is a potent anticancer molecule and can act as an HDAC inhibitor to treat various cancers [197].

In another study, Dong et al. [198] investigated the effect of the OPCML (opioid-binding protein/cell adhesion molecule) in breast cancer cells. Following the research outcome, it was found that luteolin effectively upregulated the expression of OPCML by reducing intracellular methylation levels, and downregulated intracellular methylation levels by decreasing Sp1 and NF-κB activities. The findings show that luteolin slows breast cancer cell proliferation via lowering methylation and increasing OPCML gene expression.

To reveal the molecular mechanism underlying the apoptotic effects of luteolin on human colon cancer mediated by the DNA demethylation of Nrf2 promoter and the interaction of Nrf2 and p53, Kang et al. [199] conducted a study. Luteolin increased apoptosis-related proteins and antioxidant enzyme expression, decreased DNMT expression, enhanced TET DNA demethylase expression and activity, and lowered methylation of the Nrf2 promoter area. This chemical also boosted TET1 binding to the Nrf2 promoter. These findings shed light on the mechanism through which luteolin inhibits colon cancer growth by increasing Nrf2 expression and its interaction with tumor suppressors [199].

The effects of luteolin on colorectal cancer cells were studied by Krifa et al. [200]. This chemical may cause apoptosis by activating PARP cleavage. They also looked at calpains’ role in luteolin’s proapoptotic actions. They found luteolin to be somewhat inhibitive of calpain. Luteolin was also found to increase p16INK4A, a tumor suppressor gene. This study also suggested that calpain regulates the epigenetic integrator UHRF1. Using luteolin to target calpain, UHRF1, and DNMT1 may help prevent or treat colorectal cancer.

Selvi et al. [201] reported a potential inhibitor of luteolin acetylation and found that it inhibits p300 acetyltransferase, dramatically reducing tumor growth within four weeks following a decrease in histone acetylation. Luteolin-treated cells resulted in cell cycle arrest and decreased cell migration, the upregulation of p53-induced miR-195/215 and let7C, and the downregulation of oncomiRNAs (miR-135a). The potential effects of luteolin have shown potential at several levels: in gene expression, miRNA expression, and miRNA processing [201].

Using colon cancer cells, Zuo et al. [202] in their study sought to investigate luteolin’s antitumor properties and its epigenetic potential. Luteolin suppressed cell proliferation and the cellular transformation of HCT116 and HT29 cells, decreased the methylation of the Nrf2 promoter region, and decreased the protein levels and enzymatic activities of DNMTs and HDACs. In summary, the study findings suggest that luteolin may exert its antitumor activity via epigenetic modifications [202].

Kanwal et al. [189] investigated the effect of luteolin in inhibiting the methyltransferase activity of DNA and histones. Epigenetic studies performed with luteolin exhibited a decrease in DNMT enzyme activity and a reversal of the hypermethylation of cytosine bases in the DNA and prevented cytosine methylation in the GCrich promoter sequence incubated with the M.SssI enzyme. Furthermore, a marked decrease in HMT activity and a decrease in EZH2 protein expression and H3K27 trimethylation were noted in histones isolated from luteolin-treated cancer cells. The results suggest that luteolin can alter DNMT and HMT activities and the methylation of DNA and histone proteins that regulate epigenetic modifications, thus providing a significant anticancer effect by altering epigenetic processes involved in the development of cancer [189].

#### 5.1.28. Naringin

Naringin, also known as 4′,5,7-trihydroxy flavanone 7-rhamnoglucoside, is a flavanone glycoside found in citrus fruits. Bharti et al. [256] found naringin in grapefruit flowers in 1857. The beneficial effects of naringin on metabolic syndrome are due not only to its high anti-oxidative and anti-inflammatory behavior, but also to naringin’s modification of molecular signaling pathways. Indeed, naringin regulates AMPK-, PPAR-, and CPT-1-mediated fat utilization and maintains mitochondrial activity in metabolic syndrome, obesity, and associated cardiovascular complications. Recent basic research suggests that it has anticancer and chemopreventive properties in a variety of cancers, including dental, breast, colon, liver, lung, and ovarian cancer [256]. Furthermore, naringin supplementation inhibited cell proliferation, induced apoptosis in tumor cells, and reduced movement and invasion of certain tumor cells [257].

Lewinska et al. [182] investigated whether flavonoid-induced genotoxicity can also cause cancer cell death. Cyto-and genotoxicity of Naringin in DU145 prostate cancer cell lines were investigated. Naringin decreased cancer cell number and proliferative activity in a different manner. Intracellular total ROS and superoxide production was augmented after naringin treatment. This molecule stimulated DNA double strand breaks (DSBs) and micronuclei production. Naringin was found to be a genotoxic agent in DU145 cells, which has proven its pro-apoptotic activity. It was also found to be DNA hypomethylating agents capable of modulating cancer cell epigenome, leading to changes in gene expression patterns [182].

#### 5.1.29. Pectolinarigenin

Pectolinarigenin (PEC), a natural flavonoid found in *Cirsium chanroenicum* and certain Citrus fruits, has anti-inflammatory, autophagy-induced cell death, and apoptosis-mediated anti-cancer properties [258]. To identify if this compound can inhibit STAT3 activity, Zhang et al. [203] conducted a study showing that pectolinarigenin inhibited inhibits and IL-6-induced STAT3 signaling, decreases the accumulation of STAT3 in the nucleus and blocks STAT3 DNA-binding activity in osteosarcoma cells. In this sense, they proposed a mechanism of action determined by a disruption of the formation of the STAT3/DNMT1/HDAC1 complex. Pectolinarigenin suppresses cancer cell proliferation, induces apoptosis, reduces the level of STAT3, inhibits migration and invasion, and preserves the epithelial–mesenchymal transition (EMT). The administration (intraperitoneal) of pectolinarigenin blocked STAT3 activation and impaired tumor growth and metastasis with superior pharmacodynamic properties. Overall, because of its STAT3 signaling inhibitory action, pectolinarigenin may be beneficial to treat osteosarcoma [203].

#### 5.1.30. Flavonolignans

This class of flavonoids was discovered in the standardized extract of milk thistle (*Silybum marianum*), silymarin. Since the 1970s, the flavonolignans present in silymarin have been recognized as having hepatoprotective properties [259]. Flavonolignans have been shown to have a variety of health benefits in several experiments undertaken in recent years. Flavonolignans block arachidonic acid synthesis and modulate several cell-signaling pathways, thereby reducing the development of pro-inflammatory mediators [259]. The main anticancer and epigenetic potential of this family was assessed through silibinin, one of its most active molecules [259].

#### 5.1.31. Silibinin

Silibinin, also known as silybin, is the main component of flavonolignans extracted from the seed of milk thistle plants (Silybum marianum), and has been shown to have growth inhibitory properties in cancer cells [260]. In colon cancer cells, silybin triggered cell cycle arrest and depletion of CDK2, CDK4, cyclin E, and cyclin D1 proteins, implying that Hsp90 may be a biochemical target for silybin [261].

Different studies have focused on the epigenetic potential of silibinin carried out on different cancer cells, namely human prostate cancer cell lines DU145 and PC3 [204], human bladder cell lines RT4 and T24 [205], colorectal adenocarcinoma SW480 and SW620 cell lines [206], and human NSCLC H1299 cells [207].

Anestopoulos et al. [204] hypothesized that silibinin could interfere with the epigenetic cell machinery and focused on the role of EZH2, which is frequently found deregulated in PCa. They discovered that this molecule reduced but did not fully suppress EZH2 expression in DU145 and PC3 human prostate carcinoma cell lines. This decline was correlated with a dose-dependent loss of EZH2 phosphorylation (at serine-21), to an increase in global histone H3 trimethylation at lysine-27 (H3K27me3), to the overall DNMT activity, and decreased expression of HDAC1-2. This study proposes that some of the silibinin’s anti-cancer effects may be mediated by epigenetic pathways regulated by altered EZH2 activity [204].

The study conducted by de Oliveira et al. [205] aimed to investigate the molecular events underlying the antiproliferative activity of silibinin in bladder cancer cell lines with the wild-type (RT4) and mutated (T24) TP53 gene. The results indicate that, despite the reduction in clone formation in both cell lines, the toxicogenomic effect of silibinin on FRAP/mTOR, AKT2, FGFR3, DNMT1 and miR100 was dependent on the TP53 status. Taken together, the data confirmed the role of silibinin as an antiproliferative compound, whose mechanism of action was related to the TP53 status [205].

Additionally, to determine whether silibinin alters HDAC and DNMT activity in colon cancer progression, Kauntz et al. [206] conducted a study that revealed that silibinin does not modify the activity of HDACs, but is able to significantly inhibit DNMT activity in both SW480 and SW620 cells. The clinically used HDAC inhibitor, SAHA, and the broad-spectrum HDAC inhibitor, trichostatin A, combined with silibinin, demonstrated that the synergistic effects on cell death induction may be related to its DNMT inhibitory properties. The data presented suggests that treatments combining silibinin and HDAC inhibitors may represent a promising approach, given the non-toxic nature of silibinin and the fact that HDAC inhibitors selectively target cancer cells [206].

Finally, Mateen et al. [207] assessed the effect of silibinin on major chromatin-modification enzymes to delineate their possible involvement in the observed silibinin effects. Their results show that silibinin inhibits specific HDACs, HDMs, and DNMTs, which might cooperatively contribute to the anticancer efficacy of this non-toxic phytochemical [207].

#### 5.1.32. Flavonols

Represented by a variety of molecules with multiple and confirmed bioactivity and health benefits, flavonols appear to be one the most interesting flavonoid subclasses. They are most often present in our food; onions, lettuce, asparagus, and certain varieties of berries are their primary food sources. In Europe, quercetin and its conjugates are the most consumed flavonols (about 22 mg/day) [262]. On the basis of their remarkable anticancer activity [263], fisetin, galangin, kaempferol, morin, myricetin, and quercetin were selected by different researchers to investigate their potential epigenetic regulation [263].

#### 5.1.33. Fisetin

Fisetin (3,7,3′,4′-tetrahydroxyflavone) is a flavonol widely found in fruits and vegetables, such as strawberries, apples, grapes, and cucumbers. It exhibits various activities, including neurotrophic, antioxidant, anti-inflammatory, and antiangiogenic effects [264]. Using human breast cancer cell lines (MCF-7 and MDA-MB-231), Lee et al. [146] investigated the modulatory impact of fisetin on DNA methylation catalyzed by prokaryotic SssI DNMT and human DNMT1. They found that fisetin inhibits SssI DNMT- and DNMT1-mediated DNA methylation in a concentration-dependent manner [146].

#### 5.1.34. Galangin

Galangin, a natural flavonoid present in *Alpinia officinarum* (lesser galangal), is an active component in galangal, a seasoning often used in conventional Chinese medicine. Galangin is popular and can be found in honey and propolis. Since the compound is non-toxic to humans but toxic to tumor cells, it can be used as an antineoplastic drug. Previous research has examined the antineoplastic effects of galangin, which tends to operate through a variety of mechanisms [265]. Paluszczak et al. [137] demonstrated the role of galangin on the activity and expression of DNMTs in the human breast cancer MCF7 cell line, as well as their impact on DNA and histone H3 methylation. The DNMT was inhibited by galangin but did not impact the methylation pattern or expression of RASSF1A, GSTP1, or HIN1. The overall methylation of histone H3 was also unaffected [137].

Another study examined the possible epigenetic mechanism of galangin, Zeng et al. [208] carried out research with the aim of determining an inhibitor of BACE1. They found that galangin had an impact on lowering Ab levels. Furthermore, galangin treatment resulted in a significant decrease in the levels of BACE1 mRNA and proteins [208]. They also examined whether epigenetic pathways, including histone acetylation and DNA methylation, were involved in the galangin-induced transcriptional modulation of BACE1. This molecule reduces acetylated H3 in the BACE1 promoter regions by upregulating endogenous HDAC1-mediated deacetylation, regardless of DNA methylation status. These combined results suggest a potential epigenetic action of galangin against human neuroblastoma SH-SY5Y cells [208].

#### 5.1.35. Kaempferol

Kaempferol (trihydroxy-2-(4-hydroxyphenyl)-4H-1-benzopyran-4-one) (3,5,7-trihydroxy-2-(4-hydroxyphenyl)-4H-1-benzopyran-4-one) is a flavonoid present in many culinary plants (e.g., cabbage, tea, broccoli, kale, beans, leek, pepper, and strawberries) as well as in medicinal plants (e.g., *Ginkgo biloba*, *Moringa oleifera*, *Sophora japonica*, *Tilia* spp, *Equisetum* spp, and propolis). Numerous preclinical experiments have shown that kaempferol and certain of its glycosides have a broad variety of pharmacological functions, including antioxidant, anti-inflammatory, antimicrobial, cardioprotective, antidiabetic, neuroprotective, anxiolytic, analgesic, and anticancer properties [266]. With regard to its potential epigenetic activity, several studies have been carried out [209,210,211].

Berger et al. [209] were interested in the possible HDACi activity of kaempferol and therefore carried out a systematic characterization, including an in silico docking study accompanied by in vitro screening assays. Furthermore, they investigated kaempferol in the light of hepatoma cells of human origin (HepG2 and Hep3B) and compared the reaction pattern of tumor cells to those of untransformed primary human hepatocytes, as well as in an early embryonic toxicity assay. Their findings indicate that for the first time, kaempferol was proven to have a distinct epigenetic function by inhibiting HDACs. It fits into the binding pocket of HDAC2, 4, 7, or 8 and thus binds to the zinc ion of the catalytic core, according to the in silico docking analysis. Moreover, in vitro profiling of all conserved human HDACs of classes I, II, and IV revealed that kaempferol inhibits all HDACs studied [209].

In another study, Kim et al. [210] revealed the biological activity and molecular mechanism involved in kaempferol-mediated treatment of GC cell lines. Kaempferol promoted autophagy and cell death, and increased LC3-I to LC3-II conversion and downregulation of p62 in the human GC cell lines (AGS, SNU-216, NCI-N87, SNU-638, and MKN-74) and also induced autophagic cell death via activation of IRE1-JNK-CHOP signaling, indicating an ER stress response. Kaempferol in this research induced epigenetic changes by inhibiting G9a (HDAC/G9a axis) and activating autophagic cell death [210].

Qiu et al. [211] in their research hypothesized that kaempferol could suppress DNMTs and control DNA methylation in bladder cancer due to its inhibitory effects on bladder cancer (T24 and 5637 bladder cancer cell lines). Kaempferol has been found to modulate DNA methylation in bladder cancer, causing 103 differential DNA methylation positions (dDMPs) aligned with chromosomes. They also found that kaempferol inhibits DNMT3B protein levels while having no effect on DNMT1 or DNMT3A expression [211]. On the other hand, kaempferol did not downregulate DNMT3B transcription. Interestingly, they discovered that kaempferol causes premature DNMT3B degradation by inhibiting protein synthesis with cycloheximide (CHX) and enhances DNMT3B ubiquitination by blocking the proteasome with MG132 [211].

#### 5.1.36. Morin

Morin (3,5,7,2′,4′-pentahydroxyflavone) is a common bioflavonoid isolated from Moraceae family members and is used in several plants, grapes, and wine. For decades, morin has been used as one of the largest yellow dyes for fur, cotton, and silk [134]. Morin exhibited enormous activity, in particular anticancer [267].

Nowak et al. [212] studied the effect of morine hydrate on alterations in proliferative, metastatic, and adherent capacity of human ovarian cancer cells (A2780 and SKOV-3) in the presence of decitabine, decitabine with trichostatin A. After treating all cell lines with morin hydrate, the authors observed a statistically significant reduction in adhesive and migratory capacity, as well as an accumulation of G_0_/G_1_ phase. In A2780 and SKOV-3 cells, they confirmed the effect of morin hydrate on downregulating genes thought to be upregulated and upregulating some genes thought to be downregulated during EMT. After morin hydrate therapy, unlike untreated control cells in both cell lines, the phenotypic differences correlated with molecular changes in cells, such as a drop in the expression level of genes associated with adhesion and a rise in downregulated genes during EMT. Indeed, morin could become an essential nutraceutical product useful in traditional anti-cancer therapy as well as in the prevention of carcinogenesis in the foreseeable future [212].

#### 5.1.37. Myricetin

Myricetin (3,5,7,3,4,5-hexahydroxyflavone cannabiscetin) is a natural flavonol found in a variety of fruits, spices, herbs, teas, red wines, and medicinal plants. Myricetin has antioxidant, anti-inflammatory, antiviral, and anticarcinogen properties, as well as the ability to induce apoptosis in multiple cancer cell lines [268]. Its epigenetic potential was reported by Paluszczak et al. [137] who studied its activity on MCF7 breast cancer cells. Myricetin inhibited DNMT but had no impact on the methylation pattern or RASSF1A, GSTP1, and HIN1 expression. The overall methylation of histone H3 was unaffected. The conclusion of this study showed that myricetin as a non-nucleoside agent is unlikely to be an effective epigenetic modulator [137].

#### 5.1.38. Quercetin

Quercetin, also known as sophoretin, meletin, xanthaurine, or quercetol, is an antioxidant widely dispersed in plant food sources. In human food, it is a polyphenolic bioflavonoid with antidiabetic, anticarcinogenic, anti-inflammatory, antiulcer, and antiviral effects [269]. It was one of the most exploited flavonoids in epigenetic research and many studies aimed to reveal its regulatory potential on several cancer cell lines [269].

Indeed, Lee et al. [146] studied the modulatory effects of several bioflavonoids including quercetin on DNA methylation catalyzed by prokaryotic SssI DNMT and human DNMT1 on human breast cancer cell lines (MCF-7 and MDA-MB-231). According to the results obtained, quercetin inhibited SssI DNMT- and DNMT1-mediated DNA methylation in a concentration-dependent manner [146]. This study suggests that quercetin inhibits DNA methylation through two mechanisms, namely direct inhibition of DNMT and indirect inhibition of enzymes by increased SAH formation (a potent non-competitive inhibitor of DNMTs) [146].

Based on early studies suggesting that quercetin could play a protective role in vitro and in vivo against colon cancer, Tan et al. [213] selected the human colon cancer cell line RKO to investigate the epigenetic potential of quercetin in vitro. RKO cells were treated with different concentrations of this molecule, capable of inhibiting RKO cell growth. Untreated RKO cells demonstrated almost complete methylation of the *p16^INK4a^* gene. The hypermethylation of this gene was successfully reversed after 120 h of treatment with quercetin. The *p16^INK4a^* gene expression was also restored in a concentration-dependent manner. The combined results suggest that quercetin has antitumor properties, probably via the demethylation of the *p16^INK4a^* gene promoter [213].

Tao et al. [214] studied the effect of quercetin on cell proliferation in human breast cancer cell lines MCF-7 and MDA-MB-231 with/without transfection of miR-146a mimic or anti-miR-146a [214]. Consequently, quercetin inhibited the proliferation of human breast cancer cells by upregulating miR-146a expression, inducing apoptosis through caspase-3 activation and mitochondrial-dependent pathways, and by inhibiting invasion via EGFR downregulation [214].

Zhang et al. [215] studied the efficacy of quercetin in combination with cisplatin in the treatment of osteosarcoma and the mechanisms that control it. The results showed that quercetin at concentrations of 10 μM or more inhibited 143B cell proliferation, while at 5 μM it increased 143B cell cisplatin sensitivity. After treatment with quercetin and/or cisplatin, miR-217 expression was upregulated, while its target KRAS was downregulated at mRNA and protein levels. MiR-217 knockdown resulted in a lack of cisplatin sensitivity, while miR-217 overexpression was reversed, meaning that quercetin modulated the miR-217-KRAS axis to control cisplatin sensitivity. Finally, by modulating the miR-217-KRAS axis, quercetin (5 μM) increased cisplatin sensitivity [215].

Appari et al. [165] investigated whether quercetin has anti-CSC efficacy and whether upregulation of miR-let-7 and downregulation of K-ras are involved. Their results showed that activation of apoptosis, inhibition of self-renewal ability, migration, and expression of the matrix metalloproteinases MMP-9 and MMP-2 were enhanced by quercetin, which also caused induction of miR-let-7 and inhibition of its target gene K-ras.

In their research, Kedhari Sundaram et al. [216] evaluated the potential of quercetin as a modulator of the epigenetic pathway for anticancer strategies. In a dose-dependent manner, quercetin modulated the expression of various chromatin modifiers and inhibited the function of DNMTs, HDACs, and HMTs. Depending on the results of molecular docking, quercetin can serve as a competitive inhibitor by interacting with residues in the catalytic cavity of many DNMTs and HDACs. It reduced global DNA methylation levels in a dose and time dependent manner. With the restoration of their expression, the studied TSGs demonstrated a strong dose-dependent decrease in promoter methylation [216]. This research contributes to a better understanding of quercetin’s mechanism of action, which will facilitate its development as a candidate for anticancer therapy based on epigenetics [216].

Another study conducted by Kundur et al. [217] examined the effects of quercetin on tumor suppressor gene re-expression in TNBC. Indeed, TNBC cell lines were exposed to quercetin, which increased BRCA1 expression, thus inhibiting cell survival and migration. Quercetin tended to trigger histone acetylation in the BRCA1 promoter [217]. Furthermore, quercetin therapy greatly reduced BRCA1 knockdown-induced cell viability and migration in ER+ cells. The findings also show that quercetin therapy induced anticancer activity in TNBC cells by modulating tumor suppressor genes [217].

To learn more about the fundamental mechanism of the apoptosis-inducing effects of quercetin on epigenetic control, Tseng et al. [218] examined DNA fragmentation, PARP, and procaspases, and revealed that quercetin could trigger apoptosis in human leukemia HL-60 cells in a dose-dependent manner. Quercetin activated caspase-8, causing Bid cleavage, Bax conformation transition, and cytochrome c release, which subsequently activated the extrinsic apoptosis cascade. Quercetin also activated the extracellular signal-regulated kinase (ERK) and Jun N-terminus kinase (JNK) signaling pathways, resulting in Fasligand (FasL) expression. Quercetin stimulated histone H3 acetylation, which resulted in the promotion of FasL expression in addition to activating c-Jun [218]. Quercetin can activate HAT and inhibit HADC, which leads to histone acetylation. However, only the activation impacted on HAT was linked to the ERK and JNK pathways. These findings showed that quercetin can induce FasL-related apoptosis in HL-60 cells by transactivation via activation of c-jun/AP-1 and promotion of histone H3 acetylation [218].

Lee et al. [219], performed a study to establish quercetin’s anti-EBVaGC (Epstein–Barr virus-associated gastric carcinoma) effects, finding that quercetin induces apoptosis in SNU719 cells, more completely suppresses the expressions DNMT1 and DNMT3A, and more strongly influences cell cycle progression of SNU719 [219].

Quercetin stimulates apoptosis and EBV gene transcription by inducing signal transductions. It increased the frequency with which the F promoter is used and reduces EBV latency. It also stimulated progeny development while reducing EBV infection. These findings suggest that quercetin could be a potential antiviral and antitumor candidate in the fight against EBV and human gastric carcinoma.

Liu et al. [220], in their study, investigated the effects of quercetin on hepatic DNA methylation and inflammation in nickel-exposed mice were studied by Liu et al. [220]. Quercetin administration significantly inhibits nickel-induced liver injury by decreasing the total DNMTs activity and DNA methylation level of the Nrf2 DNA in the livers of nickel-treated mice.

Priyadarsini et al. [221] discovered that the simultaneous administration of quercetin to DMBA-painted hamsters decreased tumor incidence and tumor burden, whereas post-treatment with quercetin delayed tumor development significantly. Quercetin also promoted cell cycle arrest and death and inhibited invasion and angiogenesis. The chemopreventive and therapeutic activities of quercetin were discovered to be correlated with the suppression of HDAC-1 and DNMT1.

Using the mouse small intestine epithelial cell line and the heterozygous TNFDARE/WT mice, Ruiz et al. [222] identified the molecular pathways by which quercetin suppresses TNF-induced proinflammatory gene expression. Quercetin inhibited Akt phosphorylation, macrophage inflammatory protein 2 (MIP-2) and TNF-α-induced interferon-γ-inducible protein 10 (IP-10) gene expression but did not inhibit TNF-α-induced RelA/I-kB phosphorylation and IkB degradation or TNFα-induced NF-κB transcriptional activity. Oral administration of quercetin to mice significantly inhibited IP-10 and MIP-2 gene expression in primary ileal epithelial cells but did not affect tissue pathology. The research found quercetin to be anti-inflammatory in epithelial cells via blocking cofactor recruitment at the chromatin level of pro-inflammatory genes [222].

Certain dietary phytocompounds such as quercetin and curcumin with reported DNMT-inhibitory activity were tested for their ability to re-express the AR in AR-negative CaP cell lines PC3 and DU145. Indeed, Sharma et al. [223] combined quercetin treatment with curcumin. This combinatorial treatment was much more effective than molecules alone in inhibiting DNMT, by inducing global hypomethylation, AR mRNA- and protein-level restoration, and apoptosis via the mitochondrial depolarization of PC3 and DU145. The functional AR protein expressed in cells treated with this combination [223].

Zheng et al. [224] also studied a single and a combined effect using quercetin and butyrate. Since the epigenetic alteration in tumor cells can be reversed by dietary polyphenol quercetin or butyrate with chemopreventive activity, suggesting that quercetin or butyrate can be used in chemoprevention and also as a therapeutic agent against tumors. In this study, quercetin or sodium butyrate suppressed human esophageal 9706 cancer cell growth in a dose-dependent manner, and quercetin combined with butyrate may inhibit Eca9706 cell proliferation more than that induced by these molecules separately [224]. The reverse expressions of global DNMT1, NF-κBp65, HDAC1, and Cyclin D1 were downregulated, while expressions of caspase-3 and *p16^INK4α^* were upregulated, compared to the control group; downregulated HDAC1-IR (-immunoreactivity) with nuclear translocation, and upregulated E-cadherin-IR demonstrated in immunocytochemistry treated with quercetin or butyrate, and quercetin + butyrate also displayed other negatively and positively modulated effects compared to the control group. Quercetin and butyrate, and in particular their combination, exhibited adverse effects targeting both altered DNA methylation and histone acetylation, acting as HDAC inhibitors mediated via epigenetic-NF-κB cascade signaling [224].

#### 5.1.39. Isoflavones

Isoflavones have structural similarity to estrogens, with hydroxyl groups at the C7 and C4 positions, just like estradiol. They can attach to estrogen receptors and are hence known as phytoestrogens. They have a bitter flavor and are poorly soluble in water. Isoflavones all exhibit antioxidant, anticancer, antimicrobial, anti-inflammatory, anti-osteoporotic, and estrogenic effects [270]. The anticancer effects of isoflavones have been shown to be attributable to their ability to arrest cancer cell proliferation, suppress enzyme systems associated with malignant activity, trigger apoptosis in cancer cells, and trigger cancer cell death without altering cell cycle distribution [270].

#### 5.1.40. Biochanin A

Biochanin A is present in red clover, cabbage, alfalfa, and many other herbal products. It has shown the potential to be an epidrug due to its multiple activities on various cancers such as lung cancer, prostate cancer, gastrointestinal tract cancer, pancreatic cancer, breast cancer, osteosarcoma, malignant melanoma, and tumors of the central nervous system [271].

The association of biochanin A with DNA hypermethylation was first reported by Fang et al. [225] in a study conducted on soy products describing how their oral administration inhibits tumorigenesis in different organs with different proposed mechanisms, they decided to study the effect of soy isoflavones and in particular of biochanin A. Biochanin A has been found to inhibit DNMT activity, reactivate RARb, and inhibit cancer cell growth. These results indicate that biochanin A reactivates methylation-silenced genes, and this via a direct inhibition of DNMT, which may contribute to the chemopreventive activity of dietary isoflavones.

Another study on the impact of this molecule on DNA methylation was carried out by Vandegehuchte et al. [226] using a different approach, based on the evaluation, over 21 days, of the induction of epigenetic effects in the crustacean *Daphnia Magna*, when exposed to biochanin A to study the impact on the overall DNA cytosine methylation, body length, and reproduction. Using a multi-generational experimental design and a DNA methylation analysis, the results demonstrated that biochanin A did not induce an effect on the overall DNA methylation of *D. Magna* at exposure concentrations [226].

#### 5.1.41. Daidzein

Daidzein (also known as 7-hydroxy-3-(4-hydroxyphenyl)-4H-chromen-4-one) is a naturally occurring compound present in soybeans and other legumes. It is a natural isoflavone extracted from *Pueraria Mirifica*, which belongs to the flavonoid family and to the category of biologically active secondary metabolites commonly formed during soybean development. Daidzein has been associated with various pharmacological functions, including anti-fibrotic, anti-diabetic, cholesterol-lowering, cardiovascular, and anti-carcinogenesis activity [272].

The objective of a study conducted by Dagdemir et al. was to analyze and understand the mechanisms by which phytoestrogens act on chromatin in breast cancer cell lines. In this study, two breast cancer cell lines (MCF-7 and MDA-MB 231) were treated with daidzein for 48 h in order to observe the effects of this bioactive molecule on H3 and H4 histones on H3K27me3, H3K9me3, H3K4me3, H4K8ac, and H3K4ac marks, on six selected genes (EZH2, BRCA1, ERa, ERb, SRC3, and P300). Their findings indicate that daidzein induced a decrease in trimethylated marks and an increase in acetylating marks studied on the various genes selected [227].

Vardi et al. [228], on soybean phytoestrogens, aimed to determine their effect on the methylation of promoter genes (the promoter regions of glutathione S transferase P1 (GSTP1), Ras association domain family 1 (RASSF1A), ephrin B2 (EPHB2), and breast cancer 1 (BRCA1) genes) in prostate tumors. All the promoters studied, except that of BRCA1, were strongly methylated without treatment. After treatment by daidzein, demethylation of GSTP1 and EPHB2 promoter regions was observed and an increase in their protein expression was demonstrated by immunohistochemistry. Epigenetic modifications of DNA, such as the promoter CpG island demethylation of tumor suppressor genes, might be related to the protective effect of daidzein against prostate cancer [228].

The effect of daidzein on DNA hypermethylation was conducted by Fang et al. [225] on esophageal cell lines (KYSE 510 and KYSE 150). Daidzein has been found to be less effective in inhibiting DNMT activity, reactivating RARb, and inhibiting cancer cell growth. These findings suggest that daidzein can reactivate methylation-silenced genes, possibly through a direct inhibition of DNMT, which may lead to chemopreventive function [225].

#### 5.1.42. Equol

In 1932, equol was isolated from the urine of a mare. The unexplained discovery of a high concentration of equol in rat urine and mammalian lignans, enterolactone, and enterodiol raised concerns in 1980. Equol was shown to significantly minimize hot flushes in postmenopausal women and prevent decrease in bone mineral density in middle-aged women, possibly helping to prevent the development of breast cancer [273].

The epigenic potential of Equol has been studied by Dagdemir et al. [227], whose objective was to analyze and understand the mechanisms by which this molecule acts on chromatin in breast cancer cell lines (MCF-7 and MDA-MB 231). After 48h of treatment, an effect on H3 and H4 histones on H3K27me3, H3K9me3, H3K4me3, H4K8ac, and H3K4ac marks, on selected genes (EZH2, BRCA1, ERa, ERb, SRC3, and P300) was observed. Therefore, equol caused a decrease in trimethylated marks and an improvement in acetylating marks in the genes tested.

#### 5.1.43. Genistein

Genistein is an isoflavone phytoestrogen that promotes cell division in tissue culture. It is a tyrosine kinase, histidine kinase, and topoisomerase receptor, and is considered an anticancer, antiproliferative, cardioprotective, and chemopreventive agent. However, enzyme inhibitory effects are likely to appear only at non-physiological concentrations. While at physiological concentrations, genistein stimulates the nuclear estrogen receptors ER-alpha and ER-beta and influences TGF-beta signaling pathways [274].

The impact of soy isoflavones, including genisten, on DNA hypermethylation has been studied by Fang et al. [225] who described how oral administration of this compound can inhibit tumorigenesis different organs by proposing the different possible mechanisms.

Genistein (2–20 µmol/L) reversed DNA hypermethylation and reactivated RARb, p16^INK4a^, and MGMT in KYSE 510 cells. It also inhibited cell growth at these concentrations. Reversal of DNA hypermethylation and reactivation of RARb by genistein were also observed in KYSE 150 cells and prostate cancer LNCaP and PC3 cells. Genistein (20–50 μmol/L) dose-dependently inhibited DNMT activity, showing substrate- and methyl donor-dependent inhibition. In combination with trichostatin A, SFN, or 5-aza-dCyd, genistein enhanced the reactivation of these genes and the inhibition of cell growth [225].

The aim of Dagdemir et al. [227] was to study and explain how phytoestrogens (genistein) affect chromatin in breast cancer cell lines. Using two breast cancer cell lines, MCF-7 and MDA-MB 231, genistein influenced H3 and H4 histones on H3K27me3, H3K9me3, H3K4me3, H4K8ac, and H3K4ac marks, on six genes (EZH2, BRCA1, ERa, ERb, SRC3, and P300). A decrease in trimethylated marks and an improvement in acetylating marks in the genes tested were recorded [227].

Vardi et al. [228], on soybean phytoestrogens, aimed to determine the effects on the methylation of promoter genes (the promoter regions of glutathione S transferase P1 (GSTP1), Ras association domain family 1 (RASSF1A), ephrin B2 (EPHB2), and breast cancer 1 (BRCA1) genes) in prostate tumors. All the promoters studied, except that of BRCA1, were strongly methylated without treatment. After genistein treatment, demethylation of GSTP1 and EPHB2 promoter regions was observed and an increase in their protein expression was demonstrated by immunohistochemistry. Epigenetic modifications of DNA, such as the promoter CpG island demethylation of tumor suppressor genes, might be related to the protective effect of genistein against prostate cancer as showed with other bioactive compounds [275].

### 5.2. Phenolic Acids Targeting Epigenetic Pathways in Cancer

Phenolic acids are a key class of dietary polyphenols present in a wide variety of plants. These secondary metabolites are biologically active and well documented for their medicinal values such as their antioxidant, antimicrobial, and anti-inflammatory properties [276,277,278,279]. Furthermore, phenolic acids including caffeic acid, gallic acid, protocatechuic acid, rosmarinic acid, sinaptic acid, and syringic are well known to exhibit enormous anticancer activities via different mechanisms such as the control of epigenetic events. In this context, the literature provides a wealth of information on the pathways involved in the protective effects of these bioactive compounds. The Data found on phenolic acids as epidrugs against cancer are presented in Table 2.

#### 5.2.1. Caffeic Acid

Caffeic acid is among the most studied compounds; it has potent anticancer activity across multiple targets. In a recent study, Eroglu et al. [280] evaluated the effect of phenethyl ester of caffeic acid isolated from propolis extract on the viability and proliferation of breast cancer cell lines. They showed that this compound reduced cell proliferation and induced apoptotic events via caspase 3/7 in the treated cells, highlighting that apoptosis-related genes were unmethylated. Nevertheless, the authors suggested that the combined therapy using caffeic acid with other compounds could enhance the activity and provide promising results. In the same context, Lee and Zhu [281] demonstrated that treatment of human breast cancer cells with commercial caffeic acid inhibited DNA methylation via methylation of the promoter region of the *RAR*β gene and its unmethylation status, but without any changes in the global DNA methylation, leading the authors to suggest that the observed changes may be gene-specific and were not sufficient to be detected by the experiment used [281].

Among other features of epigenetic regulation of gene expression, histone acetylation and deacetylation play an important role. Thus, HDACs represent an interesting target for cancer therapy. The purchased caffeic acid showed a promising inhibitory effect of HDACs equal to the reference compound, sodium butyrate, known by its high potential to inhibit this enzyme. This interesting finding was encouraging to analyze the structure-activity relationship of this compound, the double bond in the alkyl chain, the length of the alkyl chain, the phenolic hydroxyl (p-) group, and the 3,4-disubstitution, all have been presumed to be involved in the activity [282].

In addition to these in vitro assays, other authors have focused on the in vivo model to better understand the molecular mechanisms underlying the antitumor activity of caffeic acid. Indeed, Li et al. [283] elucidated a novel mechanism by which caffeic acid inhibits the properties of cancer stem cells in BALB/c mice. This mechanism required a direct relationship between microRNA-148a and SMAD2. In more detail, by inducing DNA methylation, the caffeic acid increased the expression of miR-148a which acted against the SMAD2-30UTR, leading to decreased expression of SMAD2 [283].

#### 5.2.2. Gallic Acid

Gallic acid has been shown to have protective effects against human cancers by targeting DNMTs. Recently, Weng et al. [284] revealed that incubation of lung cancer and pre-malignant oral cell lines with this acid for seven days induces changes in the methylome of cells, clears DNMT1 and reduces the expression level of this cytoplasmic enzyme, which could suggest that treatment with gallic acid has a strong inhibitory effect via decreasing protein stability and nuclear import. Kam et al. [285] confirmed the mechanism by which gallic acid targets DNA methylation. They showed that this compound reverses DNMT1 at the protein level, causes an accumulation of ubiquitinated protein aggregates and decreases the activities of chymotrypsin-like proteasome, which explains the protective effect of gallic acid on endothelial cell death [285,286].

#### 5.2.3. Other Phenolic Acids

Paluszczak et al. [137] evaluated the effect of a large list of polyphenols on DNA, histone H3 methylation and DNMT expression in the human breast cancer MCF7 cell line. The list included, among others, protocatechuic acid, rosmarinic acid, synaptic acid, and syringic acid. First, the results revealed that phytochemicals tested decrease the viability of MCF7 cells in a dose-dependent manner and inhibit the activity of DNMT. Indeed, rosmarinic acid was the most potent with a percentage inhibition up to 80%. At both concentrations of 20 and 40 µM, this phenolic acid significantly decreased the protein level of DNMT1 in the treated cells by 30% and 20%. While protocatechuic acid, synaptic acid, and syringic acid showed the same activities pattern with less potency compared to rosmarinic acid [137].

However, none of the studied phenolic acids exhibited an effect on the methylation process, the methylation of histone H3, and the expression of the genes including RASSF1A, GSTP1 or HIN1 in treated MCF7 cells.

**Table 2:** Phenolic acids as epidrugs.

Bioactive	Origin	Key Results	Refs.
Caffeic acid	Purchased	Inhibited the M.SssI DNMT-mediated DNA methylation (IC_50_ = 3.0 μM) Inhibited the human DNMT1-mediated DNA methylation (IC_50_ = 2.3 μM) Inhibited the DNA methylation predominantly through a non-competitive mechanism, largely due to the increased formation of S-adenosyl-_L_-homocysteine Inhibited the methylation of the promoter region of the *RAR*β gene	[281]
	Purchased	Moderately inhibited the HDAC activity (IC_50_ = 2.54 mM)	[282]
	Purchased	Attenuated the CSCs-like properties by the microRNA-148a (miR-148a)-mediated inhibition of transforming growth factor beta (TGFβ)-SMAD2 signaling pathway both in vitro and in vivo Enhanced the expression of miR-148a by inducing DNA methylation	[283]
	Not reported	Decreased the viability in MCF-7 and MDA-MB-231 cells, after 24 h of administration Decreased the cell growth in time depending on time in both cell lines No methylation effect on caspase-8 in MDA-MB-231 cell lines, but stimulation was carried out via the intrinsic pathway	[280]
Gallic acid	Purchased	Arrested the cytotoxicity in EAhy926 and HBEC-5i cells, dose-dependently, induced by the combination Reduced the formation of microparticles with anti-apoptotic effects Reversed the DNMT1 depletions at the protein level Inhibited the proteasome activities	[285]
	Purchased	Changed the methylome of lung cancer and pre-malignant oral cell lines Reduced both nuclear and cytoplasmic DNMT1 and DNMT3B within 1 week Exhibited a strong cytotoxicity against the lung cancer cell line H1299 Reactivated the growth arrest and DNA damage-inducible 45 (GADD45) signaling pathway Induced an anti-tumor pathway epigenetically reactivated by DNA demethylation in lung cancer cell	[284]
Rosmarinic acid	Purchased	Inhibited the DNMT activity (up to 88% inhibition) No effect on the methylation pattern or the expression of *RASSF1A*, *GSTP1* or *HIN-1* in MCF7 cells No effect on the global methylation of histone H3 Increased GSTP1 transcript level No effect on the expression of GSTP1 in MCF7 cells Increased DNMT1 transcript level Reduced the DNMT1 protein level in MCF7 cells	[137]
Protocatechuic acid	Purchased	Inhibited the DNMT activity No effect on the methylation pattern or the expression of *RASSF1A*, *GSTP1* or *HIN-1* in MCF7 cells No effect on the global methylation of histone H3 No effect on DNMT1 transcription or on DNMT1 protein level	[137]
Sinapic acid	Purchased	Inhibited the DNMT activity No effect on the methylation pattern or the expression of *RASSF1A*, *GSTP1* or *HIN-1* in MCF7 cells No effect on the global methylation of histone H3 No effect on DNMT1 transcription or on DNMT1 protein level	[137]
Syringic acid	Purchased	Inhibited the DNMT activity No effect on the methylation pattern or the expression of *RASSF1A*, *GSTP1* or *HIN-1* in MCF7 cells No effect on the global methylation of histone H3 No effect on DNMT1 transcription or on DNMT1 protein level	[137]

### 5.3. Stilbenes and Ketones as Targeting Epigenetic Pathways in Cancer

**Figure 6:**
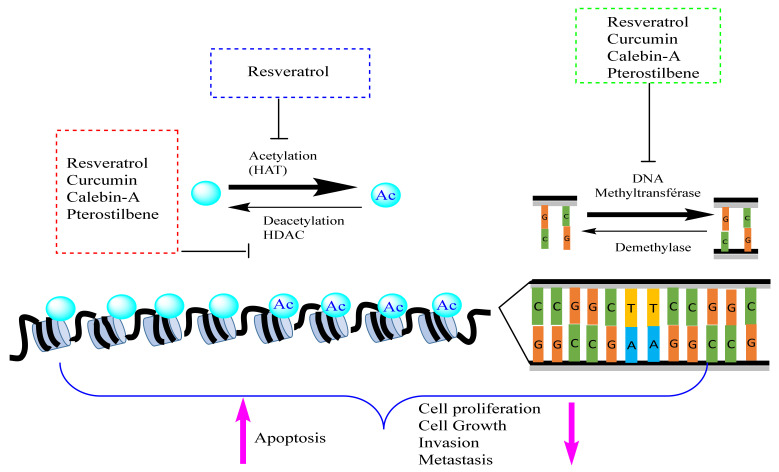
The main epigenetic action of stilbenes and ketones. *Stilbenes and ketones such as resveratrol and curcumin exhibit inhibitory actions on different epigenetic modifiers including HAT, HDAC, and DNA methyltransferase. Inhibition of epigenetic modifiers decreases cell proliferation, cell growth, invasion and metastasis, and induce apoptosis.* Abbreviations. HAT: Histone Acetyltransferase; HDAC: Histone Deacetylase.

Stilbenes and ketones are a large class of phenolic compounds comprising monomers and oligomers; the most studied is resveratrol (RSV), which has displayed many biological activities potentially beneficial to human health. Interestingly, other stilbenes also have beneficial activities such as pterostilbene and calebin-A. Some ketones such as curcumin have also shown remarkable anticancer effects by modulating the epigenomic network (Figure 6 and Table 3).

#### 5.3.1. Resveratrol

In addition to its multiple biological properties, namely antioxidant, anti-inflammatory, and neuroprotective activities [287], resveratrol has strong anticancer potential in vitro and in vivo against many types of cancers (Table 3). Wang et al. [288] conducted an in vitro and in vivo mutant model to assess the anticancer potential of RSV using mouse *Brca1* and *Brca1* wild-type cell lines. They revealed that RSV was able to activate Sirt1 and inhibit the expression of survivin leading to a greater inhibitory on *Brca1* mutant cancer cells than on *Brca1*-wild-type cancer cells. The efficacy of RSV on the *Brca1* gene using MCF-7 breast cancer cells was confirmed by Papoutsis et al. [289] where it was shown that this compound has modulated the recruitment of AhR and ERα towards the *Brca-1* promoter and antagonized the TCDD-induced histone modifications at this gene which led to an attenuation of the epigenetic changes via different mechanisms such as influencing the binding and interaction between the *Brca-1* gene of various histones and enzymes. Moreover, in breast cancer cells treated with RSV, the expression of *Brca1*, p53 and p21 was increased by enhancing the abundance of the activating histone marks, namely H3K927ac, in the proximal promoter region and reducing the enrichment of repressive histone marks, H4R3me2s and H3K27me3. In the same study, the authors highlighted other mechanisms by which RSV affected cells, including the decrease in the protein arginine methyltransferase 5 expression and the lysine deacetylase (KDAC) activity and the expression of KDAC1-3, in addition to the increased expression of lysine acetyltransferase KAT2A/3B and the global level of H3K9ac and H3K27ac marks [290].

Several studies have shown that RSV has a potential role in inhibiting DNMT activity using various cancer cells lines (Table 3) [291]. In the epithelial breast cancer MCF7 cell line, RSV has strongly inhibited the DNMT activity without influencing the global methylation of histone H3, the methylation or the expression of *RASSF1A*, *GSTP1*, or *HIN-1* in the treated cells [137]. RSV was also described to decrease the transcription levels of all the evaluated DNMTs, namely DNMT1, DNMT3a, and DNMT3b in addition to the reduction in the protein levels of DNMT1, HDAC1, and MeCP2 in human breast carcinoma cell lines MCF7 and MDA MB 231 [162]. For these same cells, this compound downregulated the DNMT [292]. Interestingly, this promising activity has been confirmed in vivo. Qin et al. [293] showed that RSV decreases DNMT3b in tumor tissue but increases its expression in normal tissues when testing the mammary tumor formation. Furthermore, in the same investigation, the authors noted that RSV influenced miRNA expression by increasing miR21, −129, −204, and −489 > twice in the tumor and decreasing the same miRs in normal tissue from 10 to 50%. Recently, it has been widely confirmed that RSV targets DNMT by inhibiting its total activity, the expression of its proteins or by modulating its gene [294,295,296,297,298,299,300].

There is ample evidence showing the ability of RSV to target the DNA and RNA, upregulate various genes, and induce apoptosis in different types of cancer. Tili et al. [301] showed that treatment of human SW480 colon cancer cells with RSV decreased the levels of several oncogenic microRNAs by acting on the genes encoding Dicer1 which is a cytoplasmic RNase III producing mature microRNAs from their immediate precursors and tumor-suppressor factors. Moreover, in this study, RSV was able to increase the levels of tumor-suppressor microRNA targeting TGFβ1 transcripts, the miR-663, and to decrease the transcriptional activity of SMADs as the main effectors of the canonical TGFβ pathway. In another investigation, it was reported that RSV upregulated the gene encoding the histone H2B ubiquitin ligase RNF20 that is a chromatin-modifying enzyme and putative tumor suppressor. In addition, this compound increased the all-tested histone markers and the expression of *p21* in MDA-MB-231 breast cancer cells, leading to growth inhibition. However, in MCF-7 cells, the RSV increased the H3K4me3 at all regions examined [302]. Using these cells, Stefanska et al. [303] demonstrated that RSV has methylated the *RARbeta2* promoter in the tested fragment by enhancing the action of 2CdA and F-ara-A towards the methylation and expression. In U87MG human glioma cells, RSV induced apoptosis and suppressed cell growth via tristetraprolin (TTP) activation. In more detail, TTP activation destabilized the urokinase plasminogen activator and urokinase plasminogen activator receptor mRNAs by binding to the ARE regions containing the 3′ untranslated regions of their mRNAs [304]. In addition, Chang et al. [305] described the inhibitory effect of RSV on mRNA and protein levels of buccal mucosal fibroblasts by blocking the expression of fibrogenic genes, downregulating ZEB1 in these lines by increasing the binding of histone H3 (H3K27me3) towards the ZEB1 promoter. RSV’s H3K27 target was confirmed by Izquierdo-Torres et al. [296] who showed that global acetylation of histone H3 and enrichment of histone mark H3K27Ac on the proximal promoter of the *ATP2A3* gene in MDA-MB-231 cells were increased due to the induction of *ATP2A3* upregulation by RSV via reduction in HDAC activity, the nuclear HDAC2 expression, and occupation of the *ATP2A3* promoter. Thus, the authors concluded that treatment of cells with RSV reduces expression of Methyl- DNA binding proteins MeCP2 and MBD2, DNMT activity and stimulates HAT activity. Furthermore, RVS enhanced promoter methylation and attenuated the protein expression in B16F10 melanoma cells by recruiting HDAC1 and DNMT3a to the promoter region of the focal adhesion kinase leading to the suppression of cell migration via epigenetic regulation [306].

It was widely observed that RSV can regulate both DNA methylation and histone acetylation by reactivating *PAX1* due to its effect on HDAC mediated as a result of the *UHRF1* downregulation in HeLa, SiHa, and Caski cell lines [307]. In the same context, the genome-wide analysis of DNA methylation of the breast cancer cell line MDA-MB-231 demonstrated the ability of RSV to restore the hypomethylated and hypermethylated status of key tumor suppressor and oncogenic genes and to increase the DNA hypomethylation [308]. In the human breast epithelial MCF10A cell line, RSV inhibited the cell growth via various mechanisms, including the decrease in DNA methylation in promoters of potential tumor suppressor genes, but its increase in regulatory regions of potential oncogenes, increased methylation levels of CpG sites in *MAML2* and *GLI2* enhancers compared to levels present in normal cells and decreased methylation of CpG sites in *SEMA3A* enhancer and the CpG island of *GLI2* leading to altered DNA methylation patterns in breast cancer cells. Therefore, treatment with RSV could prevent the invasive properties of breast cancer cells and their ability to anchorage-independent growth [309]. Recently, Fudhaili et al. [310] confirmed the effect of RSV on DNA by suppressing the DNMT2, thereby inducing the demethylation of the zinc finger protein 36 (*ZFP36*) promoter which increased expression of ZFP36 and decreased its mRNA levels target genes in A549 lung cancer cells.

The chemopreventive effect of RSV is not limited to the reported mechanisms, but also to the increase in ROS production which causes DNA damage with p-CHK1 upregulation and cellular senescence of cancer cells, reducing the mitochondrial membrane potential, a specific event of programmed cell death via apoptosis [311]. The DNA damage and the increase in apoptosis rates in the TP53 wild type cells and downregulation of AKT, mTOR, and SRC were recently confirmed in literature [299].

The context specificity of synergistic combinations provides many opportunities for therapeutic advances in natural products. To overcome the compensatory mechanisms of RVS with other compounds, Lee et al. [312] evaluated the combinatorial effect between RSV and tamoxifen in vitro and in vivo on the A2058 human melanoma, and human breast cancer cell lines (MCF7, MDA-MB231, MDA-MB468), as well as xenograft tumor models. The findings from this investigation revealed that RSV and tamoxifen more significantly reduce the viability of tumor cells and effectively block the growth of breast cancer cells compared to molecules used alone. The described mechanism of both compounds involves reduction in STAT3 acetylation, restoration of *ERα* expression, and sensitization of TNBC cells to antiestrogen therapy. In mice, the activity of the combination was accompanied by disruption of the STAT3- DNMT1 complex formation and demethylation of several tumor-suppressor gene promoters. However, the mixture of RSV and proanthocyanidins synergistically decreased cell viability and cell proliferation and synergistically reduced DNMT and HDAC activities in both cell lines MDA-MB-231 and MCF-7, while in MDA-MB-231 cells, RSV + proanthocyanidins synergistically induced apoptosis by upregulating Bax expression and downregulating Bcl-2 expression [313]. Besides, RSV and clofarabine collectively inhibited cell growth and induced caspase-3-dependent apoptosis in human erythroleukemic cell line K562. The measurement of the DNMT1 protein amounts indicated that the mixture downregulated DNMT1 and upregulated CDKN1A, with a concomitant decrease in DNMT1 protein level, while the methylation-sensitive restriction analysis allowed to note the induction of a concurrent methylation-mediated *RARB* and *PTEN* reactivation [300].

#### 5.3.2. Pterostilbene

Pterostilbene (PTS) is a well-studied analogue of resveratrol. Several investigations have proven that PTS exhibits effective anticancer properties through the inhibition of growth, downregulation of enzyme and protein expression in multiple cancer cell lines. Separately, PTS inhibited cell growth of the human breast epithelial MCF10A cell line and increased methylation at CpG#2 and/or CpG#1. Furthermore, in the same investigation, the authors revealed that PTS was able to downregulate *MAML2*, to induce alteration in DNA methylation, to decrease gene expression, and prevent the invasive properties of breast cancer cells and their ability to anchorage-independent growth [309]. Similar to these findings, Beetch et al. [298] confirmed the anticancer characteristics of PTS in addition to other mechanisms such as epigenetic activation of the tumor suppressor gene SEMA3A in breast cancer cells, decreased occupancy of DNMT3A and increased that of nuclear factor 1C within this gene promoter. Moreover, MCF10CA1a cells treated with PTS showed an increasing level of *SALL3* expression of 2.5-fold upon a 9-day treatment with 15 μm RSV.

The combination between PTS and RSV showed promising effects, which enhanced the anticancer profile of both compounds. In the study reported by Kala et al. [314] on the breast cancer cell lines (MDA-MB-157, HCC1806, and MCF10A), PTS and RSV have inhibited synergistically TNBC growth, downregulated the SIRT1 and the DNMT enzymes with no significant effects on DNMT enzyme expression in MCF10A control cells. In addition, this combined treatment has also decreased the γ-H2AX and telomerase expression leading to significant growth inhibition, apoptosis, and cell cycle arrest in the treated breast cancer cells [314].

#### 5.3.3. Calebin-A

Calbin-A is a natural analogue of curcuminoid. However, unlike curcumin, its anticancer activity has not been widely explored. Recently, Lee et al. [315] showed that calebin-A has anticancer potential in peripheral nerve sheath tumor cells by inhibiting the proliferation of MPNST and primary neurofibromas in a dose-dependent manner, by increasing the population in the G_0_/G_1_ phase, and by reducing the expression of phosphorylated-AKT, -ERK1/2, survivin, *hTERT*, acetyl H3 proteins, the promoter DNA copies of survivin (*BRIC5*), the *hTERT* genes, and the enzymatic activity of HAT without affecting that of HDAC [315].

#### 5.3.4. Curcumin

Similar to resveratrol, curcumin (Cur) has been widely reported to exhibit chemopreventive properties in numerous in vitro and in vivo assays. Different authors confirmed that Cur is an inhibitor molecule for histone modifiers that are versatile marks linked to human cancer development through various enzymatic machineries and modifications (Table 3). Khoo et al. [316] evaluated the effect of Cur on B-NHL cell line Raji and showed that Cur inhibits the proliferation through the downregulation of the expression levels of HDAC1, HDAC3, and HDAC8 proteins and upregulation of the Ac-histone H4 protein expression. In the same context, Chen et al. [317] confirmed the inhibition of Raji cells proliferation when incubated with Cur, by decreasing the amounts of p300, HDAC1, and HDAC3 and reversing the protection degradation of HDAC1 and p300 by MG-132. Indeed, the inhibition of HDAC activity by Cur was confirmed by several authors [282,318,319]. However, Kang et al. [320] showed that Cur does not display any effect on the HDAC activity in human hepatoma Hep3B cells, but was able to inhibit the HAT activity in vitro and in vivo and the histone acetylation in the absence or presence of the specific HDAC inhibitor, the trichostatin A, to decrease or enhance the ROS production, and to induce the histone hypoacetylation in vivo.

The potency of Cur on histones and proteins makes this compound an interesting apoptotic agent and motivates researchers to highlight the specific events by which Cur induce programmed cell death or arrest the cell growth of different cells. To evaluate the effect of natural Cur isolated from *Curcuma longa* on epigenetic marks, Balasubramanyam et al. [321] conducted numerous experiments, including apoptosis assay. The authors noted that Cur was able to inhibit both the p300/CREB-binding protein (CBP) HAT activity and the p300-mediated acetylation of p53 in vivo. In the human medulloblastoma cell lines (DAOY, D283 Med, and D341 Med), Cur also induced apoptosis and cell cycle arrest at the G_2_/M phase. In the same study, the tumor growth was reduced and the survival in the Smo/Smo transgenic medulloblastoma mouse model was enhanced [318]. Recently, it was extensively confirmed that Cur is an interesting example of an apoptotic agent for different types of cancer, including myeloma cell lines [320], Hepa-6 cells [322], and human gastric cancer cells [323].

Furthermore, based on Table 3, among the essential targets of Cur is the expression of miRNA and DNMT in different cells. Treatment of human pancreatic carcinoma cell line with Cur has been reported to altered miRNA expression, upregulated the miRNA-22, but downregulated miRNA-199a [324]. In colorectal cancer, Cur treatment has reduced miR-21 promoter activity and expression in a dose-dependent manner by inhibiting AP-1 binding to the promoter and induced the expression of the tumor suppressor programmed cell death protein 4, which is a target of miR-21. In addition, this treatment arrested Rko and HCT116 cells in G_2_/M phase with increasing concentrations, respectively, inhibiting the expression of miR-21 in primary tumors generated in vivo by Rko and HCT116 cells, tumors growth, invasion, and in vivo metastasis in the chicken-embryo-metastasis assay [325]. In bladder cancer cell lines, Cur induced tumor suppressor miRNA, miR-203, DNA hypermethylation of the miR-203 promoter, hypomethylation of the miR-203 promoter, upregulation of the miR-203 expression, and downregulation of miR-203 target genes Akt2 and Src [326]. In MCF-7 cells, Cur downregulated mRNA and DNMT1 protein levels and decreased the DNA methylation activity of nuclear extract [327] which was predicted for leukemia cell line in a molecular docking investigation [328,329], while the docking-based on virtual screening reported by Medina-Franco et al. [155] did not show DNA demethylation in three human cancer cell lines, which was confirmed in vitro in the study conducted by Link et al. [330] where Cur did not affect the global DNA methylation changes. Similar to RSV, the decrease in the transcript levels of several DNMTs and their regulation have been widely described in vitro and in vivo by targeting the expression of numerous enzymes and proteins [290,318,319,331,332,333,334,335,336,337].

Among other modes of action of Cur, the authors described its ability to downregulate EZH2 expression and to stimulate three major members of the MAPK pathway, JNK, ERK, and p38 kinase in breast cancer cell lines [338]. In addition, Cur affected the expression of several other enzymes and genes, namely 5 CpGs in the promoter region of the *Nrf2* gene in prostate cancer [339], the *DLEC1* promoter in HT29 cells [340], the *PAX1* promoter in cervical cancer [307], and the *GSTP1* promoter [341]. In a recent study, Cur was shown to suppress the proliferation of breast cancer cell lines and increase the mRNA and protein levels of the *Brca1* by reducing promoter methylation [342].

Based on the data discussed, Cur could be a promising chemopreventive agent. Thus, several authors have evaluated the synergistic effect between Cur and other compounds such as quercetin, decitabine, and 6-thioguanine encapsulated chitosan nanoparticles. Indeed, two studies have described the compensatory effect between Cur and quercetin. The results of these studies revealed that Cur + quercetin have inhibited the DNMT, causing global hypomethylation, restoring AR (Androgen Receptor) mRNA and protein levels, and inducing apoptosis via mitochondrial depolarization of PC3 and DU145. In addition, Cur + quercetin increased AR mRNA levels by approximately 3-fold in both cell lines [173]. The other study confirmed the promising synergistic effect between these compounds, highlighting that the combination enhanced the *Brca1* expression levels in TNBC and inhibited both the migratory ability of TNBC cell lines and the cell survival by inducing the histone acetylation of the *Brca1* promoter, and downregulation of T47D cells in the gene [217]. The combination of Cur and decitabine showed a different mechanism of action based on inhibiting the formation and migration of cancer cell colonies, the total activity of DNMT and the expression of the DNMT3a, these combination were also showed in other natural epidrugs [334,335,336,337,338,339,340,341,342,343,344]. Furthermore, Cur + decitabine regulated the tumor suppressor gene SFRP5 expression via Wnt/β-catenin signaling pathway in ovarian cell cancer line [345,346]. However, in breast cancer cell lines, this combination suppressed cell proliferation by increasing the mRNA and protein levels of *Brca1* due to decreased promoter methylation, the downregulation of both DNMT1 expression and miR-29b, and the upregulation of TET1 and DNMT3. In addition to a decrease in the mRNA and protein levels of SNCG (proto-oncogene γ synuclein) in the treated cells by inducing promoter methylation [347]. Regarding the nanoparticle’s formulation, Cur + 6-TG-CNPs affected cell viability, induced early apoptosis, arrested the G_2_/M phase, and influenced the DNA methylation activity [348].

**Table 3:** Stilbenes and ketones as epidrugs.

Bioactive	Origin	Key Results	Refs.
Resveratrol (RSV)	Not reported	Activated Sirt1 and inhibited the expression of Survivin, which caused a more profound inhibitory effect on *Brca1* mutant cancer cells than on *Brca1*-wild-type cancer cells both in vitro and in vivo	[349]
	Purchased	Inhibited the DNMT activity No effect on the methylation pattern or the expression of *RASSF1A*, *GSTP1* or *HIN-1* in MCF7 cells No effect on the global methylation of histone H3	[137]
	Purchased	Modulated the recruitment of the AhR and ERα to the BRCA-1 promoter Antagonized the TCDD (Tetrachlorodibenzo-p-dioxin)-induced histone modifications at the BRCA-1 gene Attenuated the epigenetic changes (reduced association at the BRCA-1 gene of AcH4 and AcH3K9; increased association of mMH3K9, DNMT1, and MBD2; and accumulation of DNA strand breaks)	[289]
	Purchased	Methylated, partially, the *RARbeta2* promoter in the tested fragment in MCF-7 cells Improved the action of 2CdA (2-chloro-2′-deoxyadenosine) and F-ara-A (9-beta-_D_-arabinosyl-2- fluoroadenine) on *RARbeta2* methylation and/or expression	[303]
	Purchased	Decreased the levels of several oncogenic microRNAs targeting genes encoding Dicer1, a cytoplasmic RNase III producing mature microRNAs from their immediate precursors, tumor-suppressor factors such as PDCD4 or PTEN, as well as key effectors of the TGFβ signaling pathway Increased the levels of miR-663, a tumor-suppressor microRNA targeting TGFβ1 transcripts Decreased the transcriptional activity of SMADs, the main effectors of the canonical TGFβ pathway	[301]
	Not reported	RSV + tamoxifen significantly reduced tumor cell viability more than either drug alone RSV + tamoxifen effectively blocked breast cancer growth Reduced the STAT3 acetylation, restored *ERα* expression and sensitized TNBC (triple-negative breast cancer) cells to antiestrogen therapy Inhibited the tumor growth in mice bearing A2058 human melanoma tumors, accompanied by disruption of STAT3- DNMT1 complex formation and demethylation of several tumor-suppressor gene promoters	[312]
	Purchased	Reduced the *PTEN* promoter methylation in MCF-7 cells Downregulated the DNMT and upregulated the p21 Improved the inhibitory effects of 2CdA and F-ara-A on *PTEN* methylation in MCF-7 cells	[292]
	Purchased	Upregulated the gene encoding the histone H2B ubiquitin ligase RNF20 (ring finger protein 20), a chromatin modifying enzyme and putative tumor suppressor, in MDA-MB-231 cells Increased all histone markers tested (H3K4me3, H3K9K14Ac, H4K16Ac, and H4tetraAc) at most analysed regions in MDA-MB-231 cells Increased only H3K4me3 at all regions examined in MCF-7 cells Inhibited MDA-MB-231 breast cancer cell growth in a time- and dose-dependent manner Increased *p21* expression in a dose-dependent manner in MDA-MB-231 cells Increased RNF20 expression during a 72-h period Increased the *p21* mRNA Increased the H3K4me3 in the promoter and 5’ coding region of *p21*	[302]
	Purchased	Decreased the transcript levels of all the DNMTs investigated (DNMT1, DNMT3a, and DNMT3b) Decreased the protein levels of DNMT1, HDAC1, and MeCP2	[162]
	Not reported	Decreased the DNMT3b in tumor tissue Increased the DNMT3b in normal tissue Increased miR21, −129, −204, and −489 >twofold in tumor and decreased the same miRs in normal tissue 10–50% Influenced the tumor vs. normal tissue DNMT3b and miRNA expression	[293]
	Purchased	RSV + PTS inhibited the growth of TNBCs RSV + PTS downregulated the SIRT1 (a type III HDAC) Res + PTS decreased the γ-H2AX and telomerase expression, which resulted in significant growth inhibition, apoptosis, and cell cycle arrest in HCC1806 and MDA-MB-157 breast cancer cells RSV + PTS downregulated the DNMT enzymes with no significant effects on DNMT enzyme expression in MCF10A control cells	[314]
	Purchased	Induced the glioma cell apoptosis through activation of tristetraprolin (TTP) Increased the TTP expression in U87MG human glioma cells TTP induced by RSV destabilized the urokinase plasminogen activator and urokinase plasminogen activator receptor mRNAs by binding to the ARE regions containing the 3′ untranslated regions of their mRNAs TTP induced by RSV suppressed cell growth and induced apoptosis in the human glioma cells	[304]
	Purchased	Induced an anti-myofibroblast activity in three fBMF lines Inhibited the expression of fibrogenic genes at the mRNA and protein levels in a dose- and time-dependent manner Downregulated the ZEB1 in fBMFs, which was mediated by epigenetic mechanisms, such as the upregulated expression of miR-200c and the enhancer of zeste homolog 2 (EZH2), as well as the trimethylated lysine 27 of histone H3 (H3K27me3) Increased the binding of H3K27me3 to the ZEB1 promoter	[305]
	Purchased	Caused significant reactivation of *PAX1* expression in Caski cells Induced a striking correlation between *PAX1* reactivation and Ubiquitin-like with PHD and RING finger domains 1 (*UHRF1*) downregulation in HeLa, SiHa, and Caski cell lines Reactivation of *PAX1* was due to its effect on HDAC mediated through downregulation of *UHRF1* which can regulate both DNA methylation and histone acetylation	[307]
	Purchased	Decreased gene promoter hypermethylation Increased DNA hypomethylation Restored the hypomethylated and hypermethylated status of key tumor suppressor genes and oncogenes, respectively	[308]
	Purchased	Inhibited the cell growth in a dose- and time dependent manner in both cell lines Decreased DNA methylation within promoters of potential tumor suppressor genes Increased DNA methylation within regulatory regions of potential oncogenes Increased the methylation levels (in breast cancer cells) of CpG sites within enhancers of *MAML2* and *GLI2* toward the levels present in normal cells Decreased the methylation of CpG sites in *SEMA3A* enhancer and in the CpG island of *GLI2* Downregulated the *MAML2* in both cancer cell lines Decreased the expression of the tested genes in MCF10CA1h cells Altered DNA methylation patterns in breast cancer cells Reduced invasive properties of breast cancer cells and their ability to anchorage-independent growth	[309]
	Not reported	Decreased the gene expression APOBEC3B and DNMT1 Increased the expression of TET1 in both of cell lines	[294]
	Not reported	Inhibited the total DNMT activity and DNMT3a protein expression Regulated the tumor suppressor gene SFRP5 expression Regulated the Wnt/β-catenin signaling pathway	[295]
	Not reported	Suppressed the cell migration through epigenetic regulation Recruited HDAC1 and DNMT3a to the promoter region of the focal adhesion kinase (FAK), a key factor involved in cell adhesion Enhanced the promoter methylation Attenuated the protein expression	[350]
	Purchased	RSV + GSPs synergistically decreased cell viability and cell proliferation in both cell lines RSV + GSPs synergistically induced apoptosis in MDA-MB-231 cells by upregulating Bax expression and downregulating Bcl-2 expression RSV + GSPs synergistically reduced DNMT and HDAC activities in MDA-MB-231 and MCF-7 cells	[351]
	Purchased	Inhibited the proliferation of cancer cell lines Induced the senescence along with increase in SA-β-gal activity and regulation of senescence-associated molecular markers p38MAPK, p27, p21, Rb and p-Rb protein Induced a mitochondrial dysfunction with reduction in mitochondrial membrane potential Downregulation of MT-ND1, MT-ND6 and ATPase8 in transcript level and downregulation of PGC-1α in protein level Decreased DNMT1 and increased DLC1 expression Induced cancer cellular senescence through DLC1 in a ROS-dependent manner Increased ROS production to induce DNA damage with p-CHK1 upregulation and result in cancer cellular senescence	[311]
	Purchased	Decreased the cell proliferation and induced the DNA damage in all cell lines Reduced the number of colonies in all cell lines Increased the rates of apoptosis in the TP53 wild type cells and downregulation of AKT, mTOR, and SRC Modulated the DNMT1 gene Induced a cell cycle arrest at S phase with PLK1 downregulation in the TP53 mutated cells	[299]
	Purchased	Inhibited the cell growth in a dose- and time-dependent manner in both breast cancer cell lines Attenuated the invasive capacity and anchorage independent growth by 7 μm Activated the tumor suppressor gene *SEMA3A* epigenetically in breast cancer cells Decreased the DNMT3A occupancy within *SEMA3A* promoter Increased the nuclear factor 1C occupancy at *SEMA3A* promoter Increased the *SALL3* expression by 1.5-fold upon 9-day treatment of MCF10CA1a cells with 7 μm PTS	[298]
	Purchased	Increased the expression of *BRCA1*, p53, and p21 Decreased the expression of protein arginine methyltransferase 5 (PRMT5) and enhancer of Zeste homolog 2 (EZH2) in both breast cancer cells Decreased the lysine deacetylase (KDAC) activity and expression of KDAC1-3 Increased the expression of lysine acetyltransferase KAT2A/3B Increased the global level of H3K9ac and H3K27ac marks Reduced the enrichment of repressive histone marks (H4R3me2s and H3K27me3) Increased the abundance of activating histone marks (H3K9/27ac) within the proximal promoter region of *BRCA1*, p53, and p21	[290]
	Purchased	Increased the *ZFP36* (Zinc finger protein 36) expression and decreased the mRNA levels of *ZFP36* target genes in A549 lung cancer cells Suppressed the expression of DNA (cytosine-5)-methyltransferase 1 and induced the demethylation of the *ZFP36* promoter	[310]
	Not reported	RSV-induced *ATP2A3* upregulation correlated with about 50% of reduced HDAC activity and reduced nuclear HDAC2 expression and occupancy on *ATP2A3* promoter, increasing the global acetylation of histone H3 and the enrichment of histone mark H3K27Ac on the proximal promoter of the *ATP2A3* gene in MDA-MB-231 cells Boosted the HAT activity Reduced the DNMT activity Reduced the expression of Methyl- DNA binding proteins MeCP2 and MBD2	[296]
	Purchased	RSV + clofarabine inhibited the cell growth and induced caspase-3-dependent apoptosis RSV + clofarabine downregulated the DNMT1 and upregulated the CDKN1A, with a concomitant enhanced decrease in DNMT1 protein level RSV + clofarabine induced a concurrent methylation-mediated *RARB* and *PTEN* reactivation	[300]
	Purchased	Increased the CRABP2 expression and RA sensitivity of THJ-11T and UW228-2 cells Partially (3/5) demethylated five CpG methylation sites at the CRABP2 promoter region (of both cell lines) Reduced DNMT1, DNMT3A, and DNMT3B in UW228-2 cells and reduced DNMT1 and DNMT3A in THJ-11T cells in a time-related fashion	[297]
Curcumin (Cur)	*Curcuma longa*	Inhibited the p300/CREB-binding protein (CBP) HAT activity Inhibited the p300-mediated acetylation of p53 in vivo Repressed specifically the p300/CBP HAT activity-dependent transcriptional activation from chromatin but not a DNA template Inhibited the acetylation of HIV-Tat protein in vitro by p300 as well as proliferation of the virus	[321]
	Purchased	Inhibited the histone acetylation in the absence or presence of trichostatin A (the specific HDAC inhibitor) No effect on the in vitro activity of HDAC Inhibited the HAT activity both in vivo and in vitro Diminished or enhanced the ROS generation at low or high concentrations, respectively Induced the histone hypoacetylation in vivo	[320]
	Purchased	Inhibited the proliferation of B-NHL cell line Raji cells with a 36-h IC_50_ value of 24.1 ± 2.0 μmol/L Induced the Raji cell apoptosis Downregulated the expression levels of HDAC1, HDAC3, and HDAC8 proteins Upregulated the Ac-histone H4 protein expression	[316]
	Purchased	Inhibited the proliferation potency on Raji cells in vitro (IC_50_ = 25 μmol/L) Decreased the amounts of p300, HDAC1, and HDAC3 in a dose-dependent manner Reversed the protection degradation of HDAC1 and p300 by MG-132 Prevented the degradation of IκBα Inhibited the nuclear translocation of the NF-κB (nuclear factor kappa B)/p65 subunit and the expression of Notch 1	[317]
	Not reported	Altered the miRNA expression in human pancreatic cells, upregulating miRNA-22 and downregulating miRNA-199a The upregulation of miRNA-22 expression suppressed expression of target genes SP1 transcription factor (SP1) and estrogen receptor 1 (ESR1), while inhibiting miRNA-22 with antisense enhanced SP1 and ESR1 expression	[297]
	Purchased	Inhibited the HDAC activity (IC_50_ = 115 μM)	[282]
	Not reported	Covalently blocked the catalytic thiolate of C1226 of DNMT1 to exert the inhibitory effect Inhibited the activity of M. SssI (IC_50_ =3 0 nM) Induced a global DNA hypomethylation in a leukemia cell line	[328]
	Purchased	Downregulated the expression of EZH2 (enhancer of zeste homolog 2) in a dose- and time- dependent manner Decreased the proliferation in the MDA-MB-435 cell Accumulated the cells in the G1 phase of the cell cycle Stimulated three major members of the mitogen-activated protein kinase (MAPK) pathway: c-Jun NH2-terminal kinase (JNK), extracellular signal-regulated kinase (ERK), and p38 kinase	[338]
	Not reported	A small (15–20%) decrease in methylation No detectable DNA demethylation in three human cancer cell lines	[155]
	Not reported	Inhibited the PDE1–5 (phosphodiesterase) activities (IC_50_ ≅ 10^−5^ M) Decreased the PDE1 and PDE4 activities Increased the intracellular cGMP levels in a dose-dependent manner Inhibited cell proliferation and cell cycle progression by accumulating cells in the S- and G2/M-phases with enhanced expressions of cyclin- dependent kinase inhibitors Decreased the expressions of PDE1A, cyclin A, and the epigenetic integrator UHRF1 and DNMT1	[331]
	Purchased	Reversed the methylation status of the first 5 CpGs in the promoter region of the Nrf2 gene Reduced the anti-mecyt antibody binding to the first 5 CpGs of the Nrf2 promoter	[339]
	Purchased	Induced the apoptosis and cell cycle arrest at the G2/M phase in medulloblastoma cells Reduced the HDAC4 expression and activity and increased tubulin acetylation Reduced tumor growth and significantly increased survival in the Smo/Smo transgenic medulloblastoma mouse model	[318]
	Not reported	Disturbed the Sp1/NF-κB complex on DNMT1, DNMT3a, and DNMT3b promoter thereby decreasing the transactivation activity of these complexes and downregulating their mRNA and protein expression levels in AML cells in vitro and in vivo Induced a cell cycle arrest in the S-phase Increased SubG1 cell fraction	[352]
	Purchased	Reduced miR-21 promoter activity and expression in a dose-dependent manner by inhibiting AP-1 binding to the promoter Induced the expression of the tumor suppressor Pdcd4 (programmed cell death protein 4), which is a target of miR-21 Arrested the Rko and HCT116 cells in the G2/M phase with increasing concentrations Inhibited the tumor growth, invasion and in vivo metastasis in the chicken-embryo-metastasis assay Inhibited the miR-21 expression in primary tumors generated in vivo by Rko and HCT116 cells	[325]
	Not reported	Induced a tumor- suppressive miRNA, miR-203, in bladder cancer Induced a DNA hypermethylation of the miR-203 promoter Induced a hypomethylation of the miR-203 promoter and upregulated the miR-203 expression Induced a downregulation of miR-203 target genes Akt2 and Src	[326]
	Purchased	Demethylated the first 14 CpG sites of the CpG island of the Neurog1 gene Restored the expression of this cancer-related CpG-methylation epigenome marker gene Possessed limited effects on the expression of epigenetic modifying proteins MBD2, MeCP2, DNMT1, and DNMT3a Decreased the MeCP2 binding to the promoter of Neurog1 Induced different effects on the protein expression of HDACs Increased the expression of HDAC1, 4, 5, and 8 but decreased the HDAC3 Decreased the total HDAC activity Decreased the enrichment of H3K27me3 at the Neurog1 promoter region as well as at the global level	[326]
	Purchased	Enhanced the mRNA and protein levels of ras-association domain family protein 1A (RASSF1A), 1 hypermethylation-silenced TSG, and decreased its promoter methylation in breast cancer cells Decreased the DNA methylation activity of nuclear extract Downregulated the mRNA and protein levels of DNMT1 in MCF-7 cells	[327]
	Purchased	Inhibited the cell viability and proliferation in CRC cell lines Induced the demethylation of specific CpG loci in CRC cells No induction of global DNA methylation changes Validated the methylation changes at several randomly selected loci including *KM-HN-1*, *PTPRO*, *WT1*, and *GATA4* Supported the DNA methylation alterations by corresponding changes in gene expression at both up- and downregulated genes in various CRC cell lines	[330]
	Purchased	Decreased the transcript levels of all the DNMTs investigated (DNMT1, DNMT3a, and DNMT3b) Decreased the protein levels of DNMT1, HDAC1, and MeCP2	[162]
	Purchased	Downregulated the DNMT1 expression in AML cells in vitro and ex vivo Inhibited the expression of p65 and Sp1 and their association with the DNMT1 promoter Reactivated the epigenetically silenced *p15^INK4B^* Increased the proportion of cells in SubG1 phase of the cell cycle, induced the caspase cleavage, and inhibited the AML cell growth in vitro Inhibited the AML cell growth and downregulated the DNMT1 expression in vivo	[332]
	Purchased	Decreased the cell number and viability Increased the apoptotic events and superoxide level Induced toxic effects when even as low as 1 μM concentration Induced a decrease in AgNOR protein pools, which may be mediated by global DNA hypermethylation	[353]
	Purchased	Inhibited the cell proliferation and increased the apoptosis rate through the upregulation of PTEN associated with a decreased DNA methylation level Reduced the DNMT3b in vivo and in vitro Increased the MiR-29b in activated HSCs Targeted the DNMT3b	[333]
	Purchased	Suppressed anchorage-independent growth of HT29 cells Decreased the methylation of the *DLEC1* promoter in HT29 cells Increased the transcription of *DLEC1* in HT29 cells Altered the expression of epigenetic modifying enzymes in HT29 cells	[340]
	Purchased	Increased the expression of RARβ at the mRNA and protein levels in tested cancer cells Decreased the RARβ promoter methylation in lung cancer A549 and H460 cells Downregulated the mRNA levels of DNMT3b Exhibited protective effect against weight loss, because of tumor burden, in a lung cancer xenograft node mice model Repressed the tumor growth Increased the RARβ mRNA and decreased the DNMT3b mRNA in vitro	[334]
	Purchased	Cur dependent overexpression of miR-29a and miR-185 can downregulate the expression of DNMT1, 3A, and 3B, and subsequently overexpresses MEG3 Induced the DNA hypomethylation and the reexpression of silenced tumor suppressor genes in hepatocellular cancer	[335]
	Purchased	Caused significant reactivation of *PAX1* expression in HeLa and SiHa cells Induced a striking correlation between *PAX1* reactivation and Ubiquitin-like with PHD and RING finger domains 1 (*UHRF1*) downregulation in HeLa, SiHa, and Caski cell lines Reactivation of *PAX1* was due to its effect on HDAC mediated through downregulation of *UHRF1* which can regulate both DNA methylation and histone acetylation	[307]
	Not reported	Induced the DLC1 expression by demethylation of DLC1 promoter in MDA-MB- 361 cells Inhibited the proliferation of MDA-MB-361 cells via DLC1 Inhibited the DNMT1 expression Inhibited the Sp1 expression to regulate DNMT1 and DLC1 Inhibited the breast cancer growth in vivo	[354]
	Not reported	Cur + quercetin inhibited DNMT, causing global hypomethylation, restoring AR (Androgen Receptor) mRNA and protein levels and causing apoptosis via mitochondrial depolarization of PC3 and DU145 Increased the AR mRNA levels 1.5–2.0 fold in PC3 and DU145 cells Cur + quercetin increased the AR mRNA levels by approximately 3-fold in both the cell lines Induced the apoptosis in a significant number of cells Cur + quercetin induced apoptosis through mitochondrial depolarization in 34% PC3 cells and 28% DU145 cells in 48 h Cur + quercetin induced the expression of AR in androgen refractory PC3 and DU145-gene reporter assay Cur + quercetin increased the AR protein levels in PC3 and DU145 cells and remained unchanged after transfection of these cells with scrambled ARsiRNA	[223]
	Purchased	Increased the expression of TCF21 (transcription factor 21) Downregulated the expression of DNMT1	[355]
	Not reported	Reversed the liver injury in vivo and in vitro, possibly through downregulation of DNMT1, α-SMA, and Col1α1 and by demethylation of the key genes	[356]
	Purchased	Reduced the viability of the MCF-7 cells in a dose-dependent manner (IC_50_ = 20 μM) Inhibited the growth with increased amount of cell deaths at higher concentration of 20 and 30 μM Induced the DNA damage in MCF-7 cells Reversed the hypermethylation Reactivated the glutathione S-transferase pi 1 (GSTP1) protein expression in MCF-7 cells at 10 μM Induced a complete reversal of GSTP1 promoter hypermethylation and caused a re-expression of GSTP1	[341]
	Not reported	Restored the miR-143 and miR-145 expression in prostate cancer cell lines Reduced the methylation of CpG dinucleotides in miR-143 promoter Reduced the expression of DNMT1 and DNMT3B Restored the miR-143 and miR-145 expression via hypomethylation Enhanced the radiation-induced cancer cell growth inhibition and apoptosis Reduced the radiation-induced autophagy in PC3 and DU145 cells	[295]
	Purchased	Cur + decitabine inhibited the cancer cell colony formation and migration, the total DNMT activity, and DNMT3a protein expression Cur + decitabine regulated the tumor suppressor gene SFRP5 expression through Wnt/β-catenin signaling pathway	[345]
	Purchased	Inhibited the apoptotic effects, and reduced the expression of DNMT1 The relative expression of DNMT1 gene was 0.7 to 0.3	[337]
	Purchased	Affected the proliferation of all four cancer cell lines in time- and dose-dependent manner after 24 h Increased the relative expression of p21 mRNA as well as p21 protein in all four cell lines after 24 h Altered the DNMT expression and induced global DNA hypomethylation Reduced the methylation specific amplification with an increase in corresponding unmethylation specific amplification Increased the KLF4 and p53 expression Increased the p21 proximal promoter occupancy by KLF4	[290]
	Purchased	Suppressed the proliferation of myeloma cells Induced the apoptosis in myeloma cells Inhibited the expression of mTOR (mammalian target of rapamycin) in NCI-H929 cells Induced a hypermethylation of the mTOR promoter region Upregulated the expression of DNMT3	[306]
	Purchased	Cur + quercetin enhanced the *BRCA1* (breast cancer type 1 susceptibility protein) expression levels in TNBC (triple-negative breast cancer) Cur + quercetin inhibited the cell survival of TNBC cell lines Cur + quercetin enhanced the *BRCA1* expression Cur + quercetin induced the histone acetylation of *BRCA1* promoter Cur + quercetin inhibited the migratory ability of TNBC cell lines and regulated genes involved in tumor migration Cur + quercetin downregulated the T47D cells in *BRCA1*	[217]
	Purchased	Cur (20 μM) + DAC (5 μM) inhibited the cancer cell colony formation, the migration through EMT (epithelial–mesenchymal transition) process regulation, the total DNMT activity, especially in DNMT3a protein expression Cur (20 μM) + DAC (5 μM) regulated the tumor suppressor gene SFRP5 expression involved in the Wnt/β-catenin signaling pathway	[346]
	Purchased	Suppressed the proliferation of HCC-38, UACC-3199, and T47D cell lines Increased the mRNA and protein levels of *BRCA1* in HCC-38 and UACC-3199 cells by reducing promoter methylation Cur + DAC induced the demethylation of the -379 CpG site in the *BRCA1* promoter in the HCC-38 cells Downregulated the expression of DNMT1 and upregulated the TET1 and DNMT3 in HCC-38 cells Downregulated the miR-29b in HCC-38 cells Decreased the mRNA and protein levels of SNCG (proto-oncogene γ synuclein) in T47D and HCC-38 cells by inducing promoter methylation Downregulated the expression of DNMT1 and TET1 and upregulated the DNMT3 in T47D cells Upregulated the miR-29b in T47D cells	[347]
	Purchased	The IC_50_ value for MCF-7 were 15.73 μM The IC_50_ value for PA-1 were 12.89 μM Cur (IC_25_) + 6-TG-CNPs (6-thioguanine encapsulated chitosan nanoparticles) (IC_25_) on PA-1 and MCF-7 showed % cell viability of 43.67 ± 0.02 and 49.77 ± 0.05, respectively Possessed in vitro cytotoxicity potential in terms of % cell viability, early apoptosis, G2/M phase arrest, and DNA demethylation activity on PA-1 cells	[348]
	Purchased	Indicated a dose- and time-dependent significant antiproliferative effects (IC_50_~5 μM) Indicated significant apoptotic effects in all different periods Increased significantly the *ERα* gene expression quantity	[322]
	Purchased	Inhibited the proliferation, colony formation, and migration of hGCCs in a dose- and time-dependent fashion Increased ROS levels and triggered mitochondrial damage, DNA damage, and apoptosis of hGCCs Cur-induced DNA demethylation of hGCCs was mediated by the damaged DNA repair-p53-p21/GADD45A-cyclin/CDK-Rb/E2F-DNMT1 axis	[323]
Calebin-A	Not reported	Inhibited the proliferation of MPNST and primary neurofibroma cells in a dose-dependent manner. Increased the population in the G0/G1 phase but decreased in G2/M phase Reduced the expression of phosphorylated-AKT, -ERK1/2, survivin, *hTERT*, and acetyl H3 proteins Reduced the promoter DNA copies of survivin (*BRIC5*) and *hTERT* genes Reduced the enzyme activity of HAT, but not that of HDAC	[315]
Pterostilbene (PTS)	Purchased	RSV + PTS inhibited the growth of TNBCs RSV + PTS downregulated the SIRT1 (a type III HDAC) RSV + PTS decreased the γ-H2AX and telomerase expression, which resulted in significant growth inhibition, apoptosis, and cell cycle arrest in HCC1806 and MDA-MB-157 breast cancer cells RSV + PTS downregulated the DNMT enzymes with no significant effects on DNMT enzyme expression in MCF10A control cells	[314]
	Purchased	Inhibited the cell growth in a dose- and time dependent manner in both cell lines Increased the methylation at CpG#2 and/or CpG#1 in both cancer cells Downregulated the *MAML2* in both cancer cell lines Decreased the expression of the tested genes in MCF10CA1h cells Altered DNA methylation patterns in breast cancer cells Reduced invasive properties of breast cancer cells and their ability to anchorage-independent growth	[309]
	Purchased	Inhibited the cell growth in a dose- and time-dependent manner in both breast cancer cell lines Attenuated the invasive capacity and anchorage independent growth by 15 μm Identifyed 364 hypomethylated CpG sites at day 4 of RSV treatment compared to 990 hypomethylated CpG sites at day 9 of treatment Activated the tumor suppressor gene *SEMA3A* epigenetically in breast cancer cells Decreased the DNMT3A occupancy within *SEMA3A* promoter Increased the nuclear factor 1C occupancy at *SEMA3A* promoter Increased the *SALL3* expression by 2.5-fold upon 9-day treatment of MCF10CA1a cells with 15 μm RSV	[298]

## 6. Conclusions and Perspectives

In this work, the involvement of epigenetic pathways in cancer development and its promotion were discussed. The different epigenetic regulations play a promoting role in the induction of genetic instability and tumor transformation. All of the epigenetic regulators make it possible to maintain cellular memory and the disturbances affecting these pathways predispose via the various actions to memory loss and cancerization of normal cells. Epigenetic changes are reversible and their inhibitions and/or their activations are possible using pharmacological drugs. It was showed in this review that natural substances, in particular flavonoids and phenolic acids, exert actions on epigenetic modifiers such as DNMT, HDAC, and HMT. Indeed, these epi-drugs have proven to be highly effective against various cancers in vitro and in vivo. The pharmacodynamic action of anticancer epidrugs showed specificity towards major epigenetic targets. However, the pharmacokinetic pathways of these substances are not well studied and should be investigated to verify and validate their bioavailability and metabolism. Moreover, the toxicity of these epidrugs must also be investigated to confirm their safety. The study showed that the clinical development of natural epidrugs could reveal bioactive substances in chemotherapy with a specific epigenetic action. Some of these molecules could also be applied against specific targets in chemoprevention.

## Data Availability

Not applicable.

## Abbreviations

2-HG	2-HydroxyGlutarate
5-caC	5-Carboxylcytosine
5-fC	5-Formylcytosine
5-hmC	5-HydroxymethylCytosine
5-mC	5-MethylCytosine
ACF	ATP-utilizing Chromatin Assembly and Remodeling Factor
ALKBH5	α-Ketoglutarate-dependent dioxygenase alkB Homolog 5
AML	Acute Myeloid Leukemia
APC	Adenomatous Polyposis Coli
Arg	Arginine
ARID1A	AT-Rich Interaction Domain 1A
ATG	Autophagy Related
BAF	BRG1/BRM Associated Factor
Bcl-2	B-Cell Lymphoma 2
BET	Bromodomain and Extraterminal Domain
BNIP3	BCL2 Interacting Protein 3
BPTF	Bromodomain PHD Finger Transcription Factor
BRCA	Breast Cancer
Brg1	Brahma-Related Gene 1
CA9	Carbonic Anhydrase 9
CCND2	Cyclin D2
CCT4	Chaperonin-Containing tailless complex polypeptide, subunit 4
CDC37	Cell Division Cycle 37
CDH1	Cadherin 1
CDH3	Cadherin 3
CDKN2A	Cyclin Dependent Kinase Inhibitor 2A
CERF	CECR2-Containing Remodeling Factor
CGIs	CpG Islands
CHD	Chromodomain-Helicase-DNA-binding protein
CHRAC	Chromatin Accessibility Complex
CML	Chronic Myeloid Leukemia
COX2	Cyclooxygenase 2
CRC	Colorectal Cancer
CTHRC1	Collagen Triple Helix Repeat Containing 1
DAMP	Damage-associated molecular pattern
DAPK	Death associated protein kinase 1
DGGR8	DiGeorge syndrome critical region gene 8
DLBCL	Diffuse Large B-Cell Lymphoma
DMRs	Differentially Methylated Regions
DNA	Deoxyribonucleic Acid
DNMT	DNA MethylTransferases
DOT1L	Disruptor of Telomeric silencing 1-Like
EGFR	Epidermal Growth Factor Receptor
EMT	Epithelial-Mesenchymal Transition
ER	Estrogen Receptor
ERCC1	Excision Repair Cross-Complementation Group 1
ERK	Extracellular signal-Regulated Kinase
ERV	Endogenous Retro-Viruses
ESCC	Esophageal Squamous Cell Carcinoma
EZH2	Enhancer of Zeste Homolog 2
FHIT	Fragile Histidine Triad diadenosine triphosphatase
FTO	Fat-mass and obesity associated protein
GDHO	Global DNA Hypomethylation
GNAT	GCN5-related N-acetyl transferases
GSTP1	Glutathione S-Transferase P1
H	Histone
HAT	Histone Acetyl Transferase
HATi	HAT inhibitor
HCAs	Heterocyclic Amines
HCC	Human hepatocellular Cancer
HDAC	Histone Deacetylases
HDM	Histone Demethylases
HIF	Hypoxia-Inducible Factor
HK2	Hexokinase 2 Enzyme
hMLH1	Human MutL Homolog 1
HMMR	Hyualuronan-Mediated Motility Receptor
hMSH2	Human MutS Homolog 2
HMT	Histone Methyltransferases
HNRNP	Heterogeneous Nuclear Ribo-Nucleo-Proteins
HNSCC	Head and Neck Squamous Cell Carcinoma
hOGG1	Human 8-Oxoguanine DNA N-Glycosylase 1
HOTAIR	Hox Transcript Antisense RNA
HOXA5	Homeobox A5
hOXC	Human homeobox C
HRAS	Harvey Rat Sarcoma viral oncogene homolog
HSP90	Heat Shock Protein 90
HSPGs	Heparan Sulfate ProteoGlycans
hTERT	Human Telomerase Reverse Transcriptase
IDH1	Isocitrate Dehydrogenase NADP+1
IGF2BPs	Insulin-like Growth Factor 2 mRNA-Binding Proteins
IL10	Interleukin 10
IL11	Interleukin-11
INO80	Inositol requiring 80
ISWI	Imitation switch
K	Lysines
KDM	Lysine-Specific Demethylase
KG	Ketoglutarate
KMT	Lysine-specific Methyltransferase
let-7a	Lethal-7a
lincRNA	Large Intergenic Non-Coding RNA
LINES	Long interspersed transposable elements
lncRNA	Long non coding RNA
LSD	Lysine-Specific Demethylases
Lys	Lysine
m^6^A: N^6^	Methyladenosine
MALAT1	Metastasis-Associated Lung Adenocarcinoma Transcript 1
MAPK	Mitogen-Activated Protein Kinase
MBD	Methyl Binding Protein
MBPs	Methyl-CpG Binding Proteins
MCRS1	Microspherule protein 1
MDR1	Multi-Drug Resistance 1
MECP	Methyl CpG binding Protein 2
MEF2	Myocyte Enhancer Factor 2
METTL	Methyltransferase-Like Protein
MGMT	O6-Methylguanine-DNA Methyltransferase
miRNA	Micro RNA
mRNAs	Messenger RNAs
MTA	Metastasis-Associated Gene
MTOR	Mammalian Target of Rapamycin
MyoD	Myogenic Activator
NCR	Natural Cytotoxicity Receptor
ncRNAs	Non-coding RNAs
NF-κB	Nuclear Factor Kappa light chain enhancer of activated B cells
NK	Natural Killer
NoRC	Nucleolar-Remodeling Complex
NSCLC	Non-Small-Cell Lung Cancer
NuRD	Nucleosome-Remodeling Deacetylase
NURF	Nucleolar-Remodeling Factor
PAX2	Paired box 2
PDCD4	Programmed Cell Death 4
PI3K	Phosphoinositide 3-Kinase
piRNA	PIWI-interacting RNA
PRC2	Polycomb Repressive Complex 2
PTEN	Phosphatase and Tensin Homolog
PTM	Post-Transcriptional Modifications
R	Arginine
RB gene	Retinoblastoma gene
RB1	RB transcriptional corepressor 1
RBBP	Retinoblastoma Binding Protein
RBM15	RNA-Binding Motif protein 15
RBM15B	RNA-Binding Motif protein 15B
RISC	RNA Induced Silencing Complex
RNA	Ribonucleic Acid
ROD1	Regulator of Differentiation 1
ROS	Reactive Oxygen Species
rRNAs	Ribosomal RNAs
RSF	Remodeling and Spacing Factor
RUVBL1	RuvB Like AAA ATPase 1
RUVBL2	RuvB Like AAA ATPase 2
S	Serine
SAM	S-Adenosyl-Methionine
Ser	Serine
SERPINE2	Serpin family E member 2
SINES	Short Interspersed Transposable Elements
SIRT	Sirtuin
SNF2H	Sucrose Non-Fermenting 2 Homologue
SNF2L	Sucrose Non-Fermenting 2 Like
sno-RNA	Small Nucleolar RNA
STAT3	Signal Transducer and Activator of Transcription 3
SWI/SNF	Switching defective/sucrose nonfermenting
TDG	Thymine-DNA glycosylase
TE	Transposable elements
TET	Ten eleven translocation
TET2	TET methylcytosine dioxygenase 2
TGF-β1	Transformting growth factor beta 1
Thr	Threonine
TLS	Translocated in liposarcoma
TOP2A	Topoisomerase II α
tRNAs	Transfer RNAs
TSS	Transcription Start Site
T-UCR	Transcribed Ultraconcerved Regions
TUG1	Taurine-Upregulated Gene 1
Tyr: T	Tyrosine
Tyr	Tyrosine
UHRF1	Ubiquitin like with PHD and Ring Finger domains 1
UTR	Untranslated region
VEGF	Vascular Endothelial Growth Factor
VHL	Von Hippel-Lindau syndrome
VIRMA	Vir-like m^6^A Methyltransferase Associated
WNT	Wingless
WRN	Werner syndrome protein
WTAP	Wilms Tumor 1-Associated Protein
WWOX	WW Domain Containing Oxidoreductase
Xi	Inactive X chromosome
XIC	X Chromosome Inactivation
*Xic*	X-Inactivation Center
XRCC1	X-Ray Repair Cross-Complementing 1
YTH	YT521-B Homology
YTHDF2	YTH N6-Methyladenosine RNA binding protein 2
YY1	Yin Yang 1
ZC3H3	Zinc finger CCCH domain-containing protein 13
